# The bromodomain inhibitor OTX015 (MK-8628) exerts anti-tumor activity in triple-negative breast cancer models as single agent and in combination with everolimus

**DOI:** 10.18632/oncotarget.13814

**Published:** 2016-12-07

**Authors:** Ramiro Vázquez, María E Riveiro, Lucile Astorgues-Xerri, Elodie Odore, Keyvan Rezai, Eugenio Erba, Nicolò Panini, Andrea Rinaldi, Ivo Kwee, Luca Beltrame, Mohamed Bekradda, Esteban Cvitkovic, Francesco Bertoni, Roberta Frapolli, Maurizio D'Incalci

**Affiliations:** ^1^ Laboratory of Anti-tumor Pharmacology, IRCCS-Istituto di Ricerche Farmacologiche Mario Negri, Milan, Italy; ^2^ Oncology Therapeutic Development, Clichy, France; ^3^ Radiopharmacology Department, Curie Institute-René Huguenin Hospital, Saint Cloud, France; ^4^ Institute of Oncology Research (IOR), Bellinzona, Switzerland; ^5^ Dalle Molle Institute for Artificial Intelligence (IDSIA), Manno, Switzerland; ^6^ Swiss Institute of Bioinformatics (SIB), Lausanne, Switzerland; ^7^ Oncoethix GmbH (formerly Oncoethix SA), Merck Sharp and Dohme Corp., Switzerland; ^8^ Oncology Institute of Southern Switzerland (IOSI), Bellinzona, Switzerland

**Keywords:** OTX015 (MK-8628), bromodomain inhibitor, triple-negative breast cancer, everolimus

## Abstract

Triple-negative breast cancer (TNBC) is an aggressive and heterogeneous subgroup of breast tumors clinically defined by the lack of estrogen, progesterone and HER2 receptors, limiting the use of the targeted therapies employed in other breast malignancies. Recent evidence indicates that *c-MYC* is a key driver of TNBC. The BET-bromodomain inhibitor OTX015 (MK-8628) has potent antiproliferative activity accompanied by *c-MYC* down-regulation in several tumor types, and has demonstrated synergism with the mTOR inhibitor everolimus in different models. The aim of this study was to evaluate the anti-tumor activity of OTX015 as single agent and in combination with everolimus in TNBC models. OTX015 was assayed in three human TNBC-derived cell lines, HCC1937, MDA-MB-231 and MDA-MB-468, all showing antiproliferative activity after 72 h (GI_50_ = 75–650 nM). This was accompanied by cell cycle arrest and decreased expression of cancer stem cells markers. However, c-MYC protein and mRNA levels were only down-regulated in MDA-MB-468 cells. Gene set enrichment analysis showed up-regulation of genes involved in epigenetic control of transcription, chromatin and the cell cycle, and down-regulation of stemness-related genes. *In vitro*, combination with everolimus was additive in HCC1937 and MDA-MB-231 cells, but antagonistic in MDA-MB-468 cells. In MDA-MB-231 murine xenografts, tumor mass was significantly (*p* < 0.05) reduced by OTX015 with respect to vehicle-treated animals (best T/C = 40.7%). Although everolimus alone was not active, the combination was more effective than OTX015 alone (best T/C = 20.7%). This work supports current clinical trials with OTX015 in TNBC (NCT02259114).

## INTRODUCTION

Breast cancer comprises a group of neoplasms characterized by different morphologies, biological behaviors, forms of presentation and clinical evolution. Around 60 to 80% of these malignancies express hormone receptors (for estrogens and/or progesterone). The human epidermal growth factor receptor 2 (HER2) is also overexpressed in 20% of breast tumors, most of which are hormone receptor-negative [1]. These three receptors offer defined therapeutic targets, such as is the case with tamoxifen and trastuzumab [1, 2]. However, about 15% of breast cancers lack estrogen and progesterone receptors as well as HER2, representing an aggressive and heterogeneous subtype group known as triple-negative breast cancer (TNBC) [1]. The physiopathology of TNBC is poorly understood, thereby limiting the implementation of targeted therapies. Moreover, although some patients respond well to cytotoxic chemotherapies, relapses are frequent and resistance to available treatments occurs almost without exception in the metastatic setting [1, 3], highlighting the urgent need to develop alternative therapeutic strategies against TNBC [3, 4].

The signaling pathway involving PI3K, the Akt serine/threonine specific protein and the mammalian target of rapamycin (mTOR) is frequently deregulated in human breast cancers [5–7]. This is often due to loss of its suppressor, the PTEN phosphatase, as is commonly the case in TNBC [7–9]. Although inhibition of this pathway has been hailed as a promising therapeutic strategy for the treatment of TNBC, to date, no clinical results have shown the expected therapeutic effects [3, 10, 11].

Over the last few years, research into epigenetics regulation has furthered our understanding of cancer biology. The bromodomain and extra C-terminal domain (BET) protein family is comprised of four epigenetic readers (BRD2, BRD3, BRD4, and the testis-restricted BRDT) that recognize acetylated lysine residues of histones. The BET/histone binding interaction allows the recruitment and activation of RNA polymerases, inducing transcription of specific genes. Many proteins that use BETs for recruiting regulatory complexes have been implicated in cancer development. These include the oncogene *MYC* [12], commonly overexpressed in TNBC [4]. While efforts to target MYC have been largely unsuccessful, the novel triazolothienodiazepine BET inhibitor OTX015 (MK-8628) [13] has shown potent preclinical antitumor activity in hematologic malignancies [14, 15] as well as in several solid tumors [16–18], in association with down-regulated *MYC* expression. Moreover, OTX015 was the first BET inhibitor to have shown clinical activity, with three ongoing phase I clinical trials in hematologic malignancies (NCT01713582) and solid tumors (NCT02259114, NCT02296476) [19–21]. Recently, BET inhibitors have been shown to be active in TNBC pre-clinical models [22], and OTX015 demonstrated strong *in vitro* and *in vivo* synergy with the mTOR inhibitor everolimus (RAD001) in lymphoma models [14, 23]. The current study was designed to investigate the *in vitro* and *in vivo* antitumor activity of OTX015 in TNBC cell line models as a single agent and in combination with everolimus.

## RESULTS

### OTX015 antiproliferative activity in TNBC cell lines

**Figure 1:**
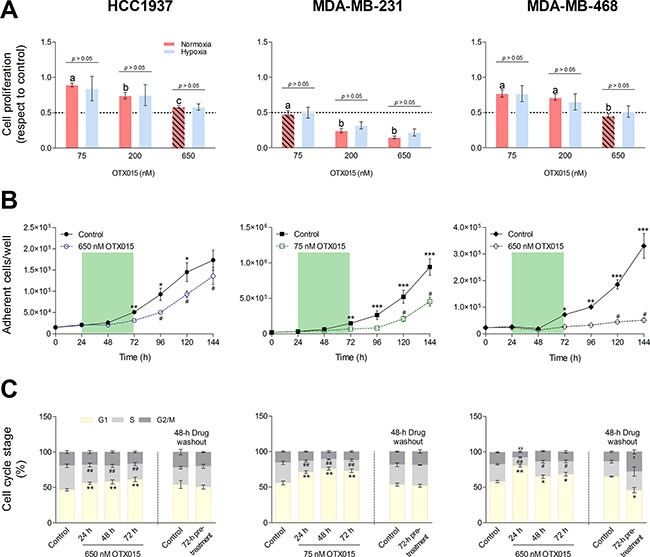
Characterization of OTX015 antiproliferative activity in three TNBC cell models (**A**) Cells were seeded under normoxic and hypoxic conditions and treated with OTX015 for 72 h. The antiproliferative effect was determined by cell counting. The diagonal-line pattern (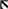) indicates OTX015 concentrations that decreased cell growth by 50% (GI_50_). For each treatment, the influence of hypoxia on antiproliferative activity was evaluated with the Student *t*-test. Significant differences in the OTX015 treatments were determined with a one-way ANOVA test (*p* < 0.01) followed by an SNK *a posteriori* test; substantial differences (*p* < 0.05) are indicated as letters above the bars. (**B**) Cell lines were treated with OTX015 for 72 h (indicated by the green area) and then cultured in drug-free medium. The *Y* axis represents the number of cells per well evaluated every 24 h after cell seeding (time 0). Significant differences in the number of control and treated cells at each time point were determined by two-way ANOVA test (*p* < 0.001) followed by a Bonferroni *a posteriori* test (*p < 0.05, **p < 0.01, ***p < 0.001). Significant differences at each time point after washout *versus* 72 h-treated cells were determined with one-way ANOVA test (*p* < 0.01) followed by an SNK *a posteriori* test (^#^*p* < 0.05). (**C**) Cells were treated with OTX015 for 24, 48 and 72 h and the latter cells were further cultured without OTX015 for 48 h (drug washout). Significant differences in the percentage of OTX015-treated cells in the G1, S and G2/M phases with respect to untreated controls were evaluated by a one-way ANOVA test (*p* < 0.01) followed by an SNK *a posteriori* test. After washout, differences in the percentage of cells in a given cell cycle phase between control and pretreated cells were determined with the Student *t*-test. Significant differences between treated cells and controls were reported for the G1 phase (*p < 0.05, **p < 0.01), S phase (^#^*p* < 0.05, ^##^*p* < 0.01) and G2/M phase (×*p* < 0.05, ××*p* < 0.01). Each dot or bar and vertical line represents the mean ± SEM, respectively (*n* ≥ 3).

Three TNBC-derived cell lines were selected: HCC1937, MDA-MB-231 and MDA-MB-468, which present different alterations in the *PTEN*, *BRCA1* and *TP53* genes [24] (Table 1). The effect of OTX015 on cell growth was determined by cell counting after 72 h of drug exposure. Although all three cell lines were sensitive, OTX015 was most active in MDA-MB-231 cells, with mean concentration that gives a half-maximal effect (EC_50_) and maximum effect (E_max_) values of 55.9 nM and 89%, respectively. On the other hand, for the HCC1937 and MDA-MB-468 cell lines the OTX015 E_max_ was ≤ 80% and the EC_50_ values were 261.5 and 303.8 nM, respectively (Table 1 and Supplementary Figure S1). As shown in Figure 1A, cell proliferation was nevertheless comparably reduced by 50% in all three cell lines when OTX015 was used at a concentration of 650 nM in HCC1937 and MDA-MB-468 cells and at 75 nM in the MDA-MB-231 cell line after 72 h of treatment. These concentrations were thus employed as pharmacologically equivalent concentrations that decreased cell growth by 50% (GI_50_) for further experiments.

**Table 1:** EC_50_ values after 72 h OTX015 exposure under normoxia and hypoxia in TNBC cell lines

	Normoxia EC_50_ (nM)	E_max_%	Hypoxia EC_50_ (nM)	E_max_%	*PTEN*	*BRCA1*	*TP53*
**HCC1937**	261.5 (38.1–1796)	70	246.9 (58.1–1049)	54	Homozygous deletion	Mutated	Mutated
**MDA-MB-231**	55.9 (45.3–69.0)	89	49.1 (24.8–96.9)	82	Wild type	Wild type	Mutated
**MDA-MB-468**	303.8 (91.5–1008)	80	230.0 (38.8–1363)	72	Homozygous deletion	Wild type	Wild type

Results are expressed as the mean concentration that gives half-maximal effect (EC_50_) with 95% confidence intervals. E_max_% indicates the maximum inhibitory response induced by OTX015 on cell proliferation (with respect to control untreated cells). In all cases, *n* ≥ 4.

The median oxygen partial pressure (*PO_2_*) in normal human breast tissue is approximately 65 mm Hg [25]. However, clinical studies have demonstrated that more than 50% of human breast cancers present a median *PO*_2_ of 2.5 mm Hg [25], a hypoxic state associated with drug resistance and anaerobic glycolysis [26]. Furthermore, OTX015-sensitive lymphoma cell lines showed increased expression of hypoxia-related genes [14]. We thus investigated the effect of hypoxia on the sensitivity of the three TNBC cell lines to OTX015. This showed that the antiproliferative effect of OTX015 was not altered by hypoxia (0.1% atmospheric O_2_) (Table 1 and Figure 1A), and subsequent studies were only performed under normoxic conditions.

To further characterize the activity of OTX015 on cell growth, cells were treated with GI_50_ concentrations for 72 h, then washed with PBS and re-incubated with OTX015-free medium. Cells were counted every 24 h over the 72-h period of OTX015 exposure as well as during the 72 h after drug washout. As shown in Figure 1B, OTX015-treated HCC1937 cells rapidly recovered their proliferative capacity once the drug was removed, showing comparable numbers (non-significant difference) to control cells 72 h after washout. Recovery of MDA-MB-231 cells was slower, showing residual OTX015 antiproliferative activity 24 h after washout. Nevertheless, cell proliferation increased (*p* < 0.05) 48 h after washout. The reversible antiproliferative effect of OTX015 in both cell lines was mirrored by changes in the cell cycle phases, as determined by flow cytometry. OTX015 induced an increase in the percentage of cells in the G1 phase after 24 h of treatment, maintained throughout the 72-h exposure (*p* < 0.01, Figure 1C). Of note, cell arrest was more marked in MDA-MB-231 cells compared to HCC1937. In both cases, this effect was accompanied by a significant reduction (*p* < 0.01) of cells in the S phase. However, after 48 h washout, both HCC1937 and MDA-MB-231 cell lines recovered the cell cycle pattern of control untreated cells (Figure 1C).

For MDA-MD-468 cells, on the other hand, a 72-h OTX015 treatment showed marked lasting inhibitory effects on their proliferative capacity (Figure 1B). Although the effect was reversible (72 h after washout the number of treated cells increased significantly, *p* < 0.05), growth was much slower compared to the two other cell lines. As for HCC1937 and MDA-MB-231 cells, OTX015 induced a substantial increase in the percentage of cells in the G1 phase after 24 h exposure with a marked decrease in the percentage of the S phase cells (*p* < 0.05; Figure 1C). Interestingly, after a 48-h drug washout there was a significant accumulation of MDA-MB-468 cells in the G2/M phase to the detriment of cells in the G1 phase (*p* < 0.05), explaining the slow recovery after OTX015 washout.

JQ1, an analog of OTX015, has been reported to induce senescence and apoptosis in SUM159 and MDA-MB-231 cells [22]. It is worth noting that 650 nM OTX015 induced morphologic changes in the MDA-MB-468 cell line, with gradual increases in cell size and the cytoplasm/nucleus ratio in a time-dependent manner (data not shown). These changes were only observed in this cell line, remaining even after 48 h of drug washout. However, neither a significant increase in senescent cells (as determined by the activity of β–galactosidase at pH 6) nor cytotoxic effects, including apoptosis, were seen in any of the three cell lines, either during OTX015 treatment or after dug washout (data not shown).

### Effect of OTX015 on c-MYC and BET-BRD protein expression

**Figure 2:**
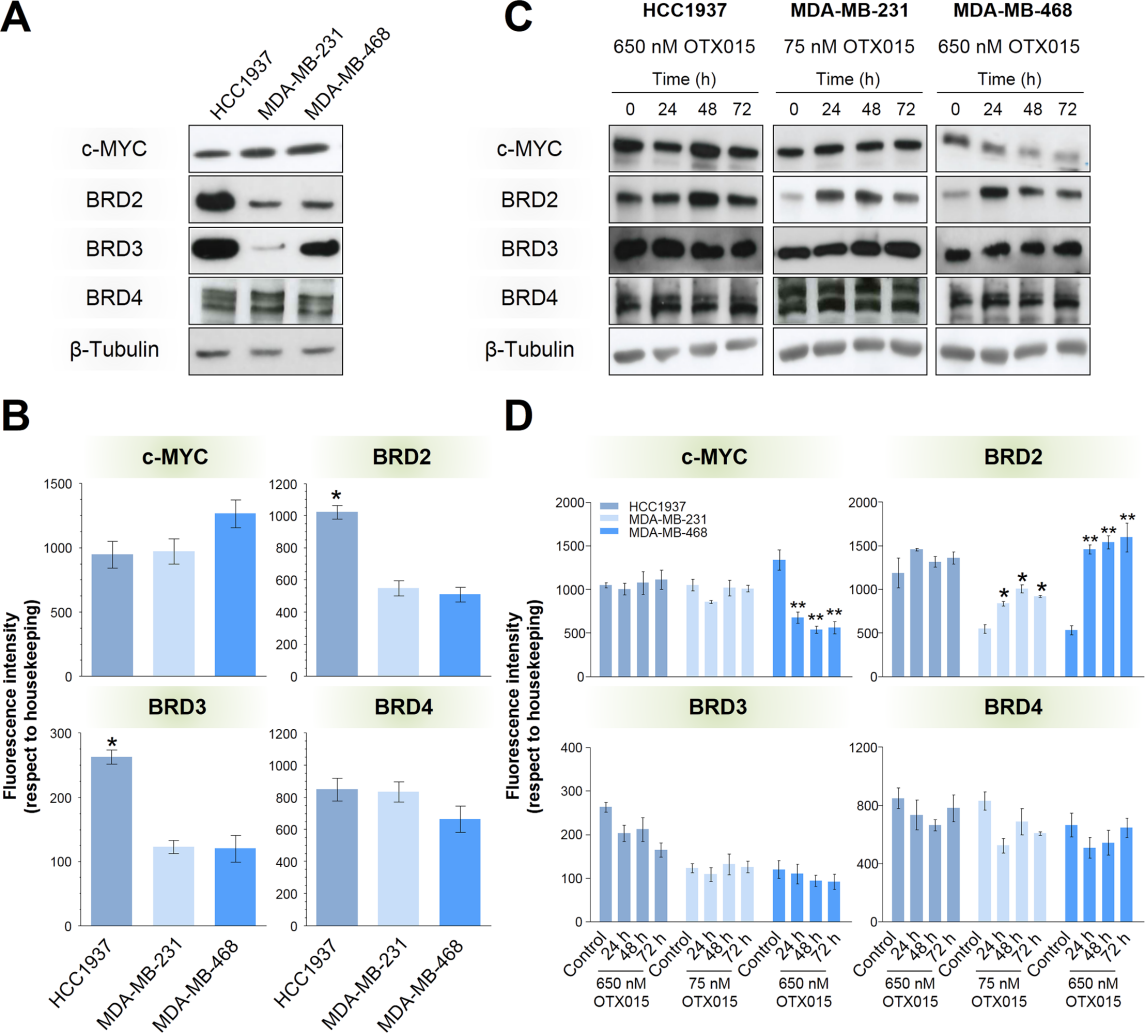
Baseline and post-OTX015 expression of BRD2/3/4 and c-MYC Basel expression of BRD2/3/4 and c-MYC in terms of protein (**A**) and mRNA (**B**) levels were evaluated in the three cell lines by Western blotting and qRT-PCR, respectively. Protein (**C**) and mRNA (**D**) levels were evaluated after 24-, 48- and 72-h OTX015 (650 nM in HCC1937 and MDA-MB-468 cells, and 75 nM in the MDA-MB-231 cell line). Significant differences in mRNA levels were determined by one-way ANOVA test (*p* < 0.01) followed by an SNK *a posteriori* test (*p < 0.05, **p < 0.01). Western blots are representative of at least three independent experiments. Bars and vertical lines represent the mean ± SEM, respectively (*n* = 3).

The three cell lines showed comparable c-MYC protein and mRNA levels, as indicated by Western blot and qRT-PCR experiments (Figure 2A and 2B, respectively). As OTX015 target proteins, basal expression of BRD2/3/4 was also evaluated. HCC1937 cells showed higher BRD2 mRNA (*p* < 0.05) and protein expression than the other two cell lines. In terms of BRD3, protein levels were higher in HCC1937 and MDA-MB-468 cell lines relative to MDA-MB-231 cells, while MDA-MB-231 and MDA-MB-468 cells showed comparable and lower mRNA levels than HCC1937 cells. Equivalent BRD4 protein and mRNA levels were detected in the three cell lines. Baseline expression of BRD2/3/4 and c-MYC did not correlate with sensitivity to OTX015 in these cell lines.

When treated with OTX015 GI_50_ concentrations, only MDA-MB-468 cells showed a significant and stable down-regulation in c-MYC protein and mRNA (*p* < 0.01) levels from 24 h of exposure (Figure 2C and 2D, respectively), suggesting the antiproliferative effects of OTX015 are c-MYC-independent in HCC1937 and MDA-MB-231 cells. BRD2 protein and mRNA were strongly up-regulated in MDA-MB-231 (*p* < 0.05) and MDA-MB-468 (*p* < 0.01) cells at 24 h, which was maintained with a gradual decrease in protein levels over 72 h. On the other hand, BRD3 and BRD4 expression remained relatively stable during OTX015 treatment in all three cell lines.

### Gene expression profiling (GEP) in OTX015-treated MDA-MB-231 and MDA-MB-468 cell lines

**Figure 3:**
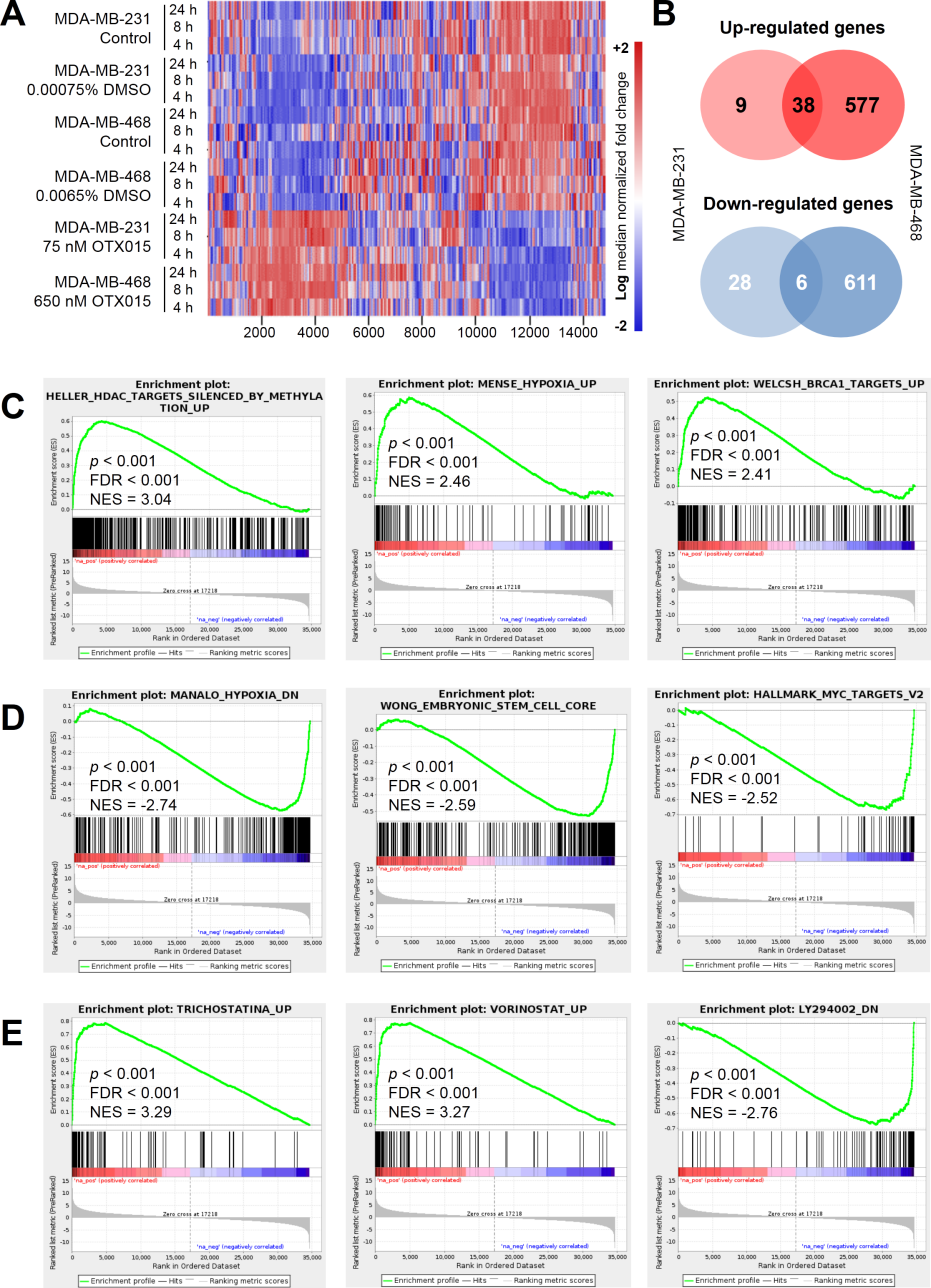
Gene expression profiling in OTX015-treated TNBC cell models (**A**) GEP heat maps in OTX015-treated MDA-MB-231 and MDA-MB-468 cell lines, compared to vehicle-treated control cells. Red and blue indicate up-regulated and down-regulated genes respectively. (**B**) Venn diagram of 81 and 1286 differentially expressed genes in MDA-MD-231 and MDA-MB-468 OTX015-treated cells, respectively. GSEA analyses showed common signatures enriched, using the complete MSigDB (**C**), up-regulated and (**D**), down-regulated) and CMAP databases (**E**) in OTX015-treated MDA-MB-231 and MDA-MB-468 cell lines. FDR, false discovery rate; NES, normalized enrichment score.

To assess the changes occurring in the transcriptome after OTX015 treatment, GEP was performed on MDA-MB-231 and MDA-MB-468 cell lines after 4-, 8- and 24-h GI_50_ exposure, compared to vehicle treatment (Figure 3A). The statistical tests identified distinct numbers of differentially expressed genes (DEGs) depending on the cell line, which is likely explained by considering that this phenomenon correlated with the GI_50_ concentrations used. Specifically, fewer changes were elicited by OTX015 treatment in MDA-MB-231 cells *vs*. vehicle (81 DEGs, of which 47 were up-regulated and 34 down-regulated; *q* < 0.05 and |LogFC| ≥ 0.5; Figure 3B and Supplementary Table S1). A much higher degree of change was observed in MDA-MB-468 cells, with 1286 DEGs (615 up-regulated, 671 down-regulated; *q* < 0.05 and |LogFC| ≥ 0.5 (full list in Supplementary Table S2). Interestingly, several of these DEGs are related to morphology and cytoskeleton remodeling, such as *TUBB3*, *TUBB2A* and *C*, *TUBB4Q*, *TUBB6*, *VAMP1* and *COL9A2*. On the other hand, only *TUBB3* and *TUBB4Q* were altered in MDA-MB-231 cells. This is coherent with the aforementioned morphological changes only observed in MDA-MB-468 cells. Other common up-regulated genes in both cell lines included histone clusters 1 and 2 (*HIST1H2BJ*, *HIST2H2BE*, etc.), *BRD2* (in line with Western blot and qRT-PCR results), and *CDKN1A* (p21^Cip/CDKN1A^ protein; Supplementary Table S3), which could explain the OTX015-mediated arrest of TNBC cells at the G1 stage of the cell cycle. Common down-regulated genes are listed in Supplementary Table S4.

Gene set enrichment analysis (GSEA) combining both MDA-MB-231 and MDA-MB-468 cell lines indicated a marked up-regulation (*p*-value and FDR < 0.01) of genes involved in epigenetic control of gene transcription and hypoxia as well as of genes regulating chromatin structure and the cell cycle (Figure 3C and Supplementary Table S5). Hypoxia-related genes were also found among the most down-regulated genes, together with those associated with stem cell-like phenotype and the set of genes modulated by MYC (Figure 3D and Supplementary Table S5). Of note, none of the hypoxia-associated genes were simultaneously affected in both (up-regulated and down-regulated) gene sets. In this context, pro-glycolytic genes such as *PFKFB3*, *PFKFB4* and *PGK1* were significantly enriched (*p*-value and FDR < 0.001). These changes were accompanied by weakened mitochondrial activity, as reflected by a marked reduction (*p*-value and FDR < 0.001) in the expression of *DLAT* (which codes for the E2 component of the pyruvate dehydrogenase complex) and genes with a key role in modulating mitochondrial protein levels (*i*.*e*. *MRPL46* and *MRPS12*). Furthermore, *c-MYC* was not a target gene among the 20 most enriched sets with a negative normalized enrichment score (NES).

OTX015 prevents BET protein-mediated recognition of acetylated lysine residues of histones, altering gene transcription patterns [12]. A GSEA using the CMAP database indicated a strong similarity (*p* and FDR < 0.001) between the GEP of OTX015 and the up-regulated and down-regulated signatures of histone deacetylase inhibitors tricostatin A (NES 3.2 and −3.0), vorinostat (NES 3.27 and −2.93), scriptaid (NES 3.13 and −3.30) and CP-690334-01 (NES 3.10 and −2.89) (Figure 3E and Supplementary Table S6). Interestingly, the fifth ranked drug in this analysis was the PI3K inhibitor LY294002 (NES 2.3 and −2.76), offering a rationale for a potential synergism between BET and PI3K/Akt/mTOR pathway inhibitors.

### OTX015 effects on cancer stem cell (CSC) markers

**Figure 4:**
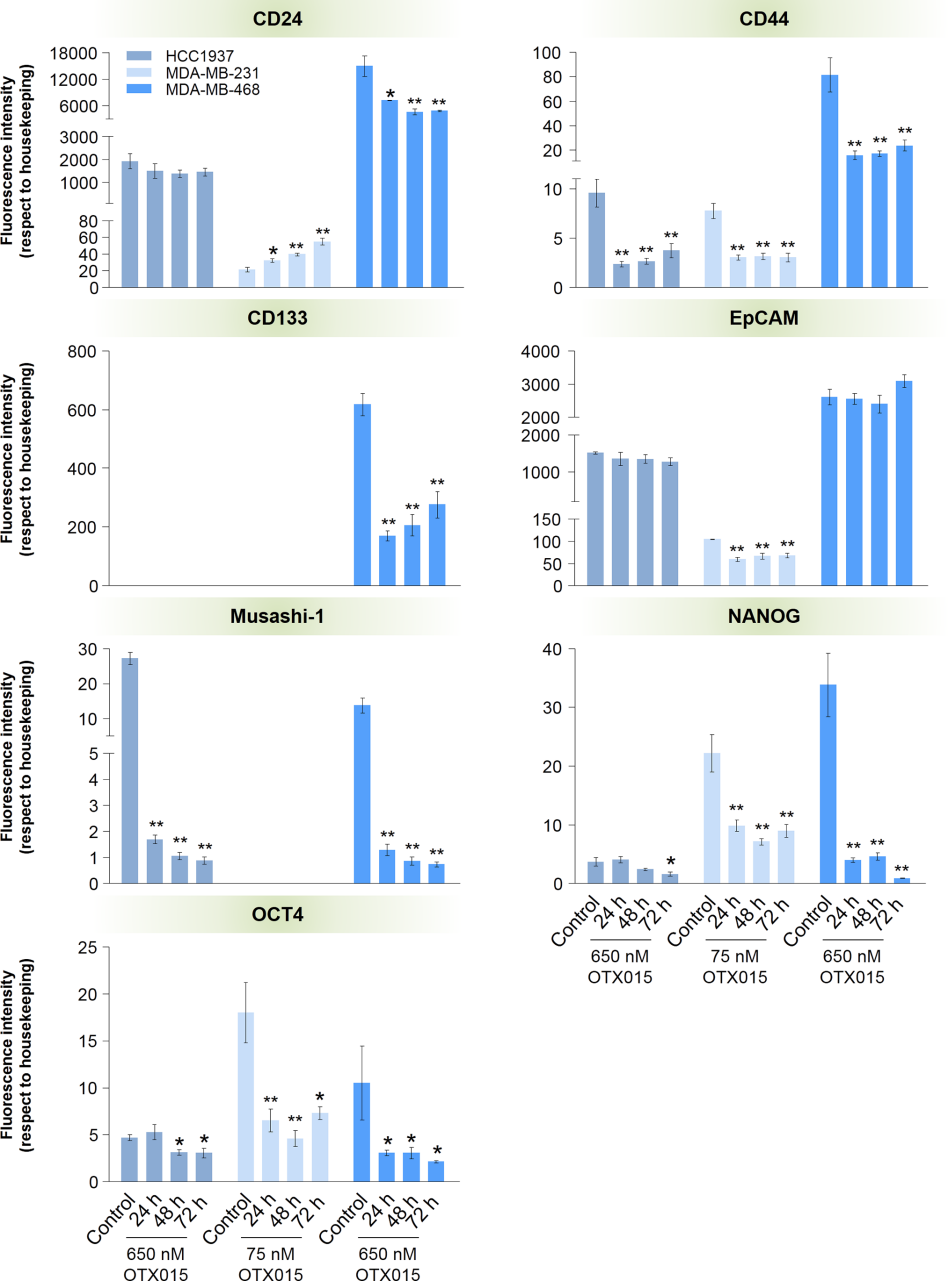
Effect of OTX015 on the expression of cancer stem cell markers in TNBC cell models Cells were exposed for 24, 48 and 72 h to OTX015 (650 nM for HCC1937 - ■ - and MDA-MB-468 - ■ -, and 75 nM for MDA-MB-231 cells - ■ -) and expression of stemness-related genes was determined by qRT-PCR. Differences in the mRNA levels of OTX015-treated cells *vs*. controls were determined by a one-way ANOVA test (*p* < 0.01) followed by an SNK *a posteriori* test (*p < 0.05, **p < 0.01). Bars and vertical lines represent the mean ± SEM, respectively (*n* = 3).

Based on the aforementioned GEP results regarding the stemness gene set, the expression levels of CSC markers were studied in the TNBC models, as shown in Figure 4. In breast cancer, CSCs are frequently identified by the expression of CD44^high^/CD24^low^ [27]. OTX015 induced a marked (*p* < 0.05) inversion of this ratio in the MDA-MB-231 cell line after 24 h of treatment. In HCC1937 cells, only CD44 mRNA levels decreased (*p* < 0.01), whereas both CSC markers diminished (*p* < 0.05) after treatment in MDA-MB-468 cells. CD133, another indicator of tumor initiating capacity, was only detected in MDA-MB-468 cells, and was significantly (*p* < 0.01) down-regulated by OTX015 after 24 h exposure. On the other hand, EpCAM, a classic epithelial marker, was detected in all three cell lines but only MDA-MB-231 cells showed decreased expression levels (*p* < 0.01) in response to OTX015 treatments. Musashi-1, a stem cell marker in breast cancer, was detected in HCC1937 and MDA-MB-468 cells, showing a strong down-regulation (*p* < 0.01) after treatment. OTX015 induced a substantial decrease in NANOG and OCT4 expression levels in the three cell lines. However, although the effect was significant after 24 h of treatment in MDA-MB-231 and MDA-MB-468 cells (*p* < 0.01 and *p* < 0.05, respectively), in the case of the HCC1937 cell line, down-regulation of NANOG and OCT4 was seen after 72 and 48 h of treatment, respectively (*p* < 0.05).

### OTX015/everolimus *in vitro* combination studies

**Figure 5:**
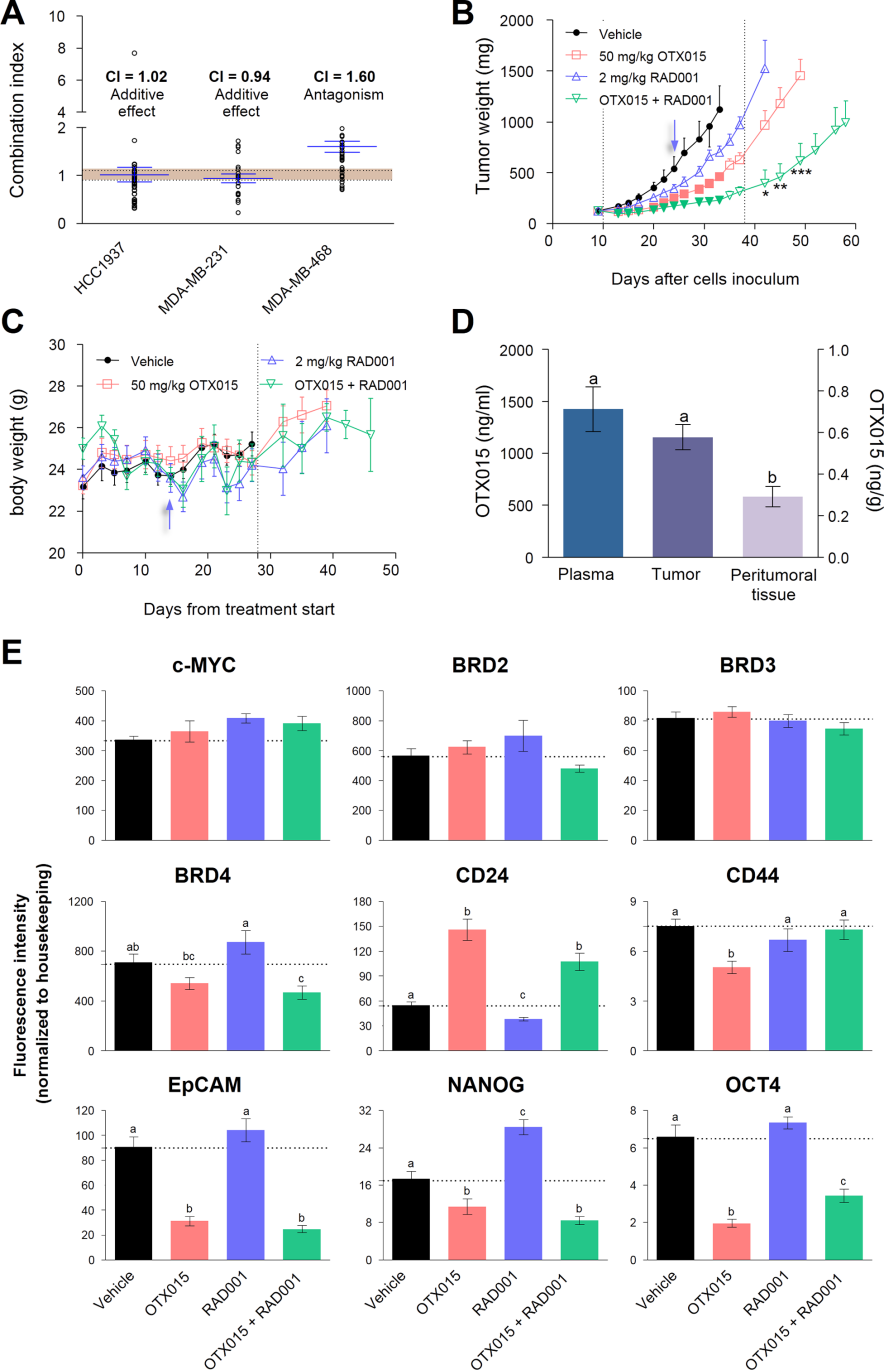
Antitumor activity of OTX015 in combination with everolimus in vitro and in MDA-MB-231 xenografts (**A**) Antiproliferative effects of concomitant OTX015 and everolimus exposure for 72 h was evaluated with the MTT assay in all three TNBC cell lines. Synergism is defined as a combination index < 0.9; values between 0.9 and 1.10 indicate an additive effect (shown as dotted lines); and values > 1.10, indicate antagonism (*n* = 3). (**B**) Antitumor effects of 50 mg/kg OTX015 twice daily (□) and 2 mg/kg everolimus thrice daily (RAD001; 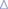) were evaluated as single agents and in combination (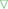) in MDA-MB-231 murine xenografts. Tumor weight was compared between drug regimens at each time point using a two-way ANOVA test (*p* < 0.001) followed by Bonferroni *a posteriori* test on log-transformed data. Color-filled symbols indicate significant (*p* < 0.05) differences between treated *vs*. vehicle mice (·). Significant differences in tumor mass between the combination *versus* single agent treatment after drug withdrawal are shown (*p < 0.05, **p < 0.01 and ***p < 0.001). Dotted lines define the treatment period. (**C**) Animal body weight during treatment (dotted lines) and subsequently. Results represent the mean ± SEM (during treatment, *n* = 9). Arrows in B and C indicate the change in the everolimus dosing schedule. (**D**) OTX015 levels in plasma and tissues from OTX015-treated mice, 4 h after the last dose. Variations in OTX015 concentrations detected between plasma, tumor and peritumoral tissue were evaluated by a one-way ANOVA test (*p* < 0.01) followed by an SNK *a posteriori* test. Letters above the bars indicate substantial differences (*p* < 0.05). (**E**) mRNA levels of *c-MYC*, *BRD2/3/4*, and CSC markers were determined by qRT-PCR in MDA-MB-231 tumors after 4-week treatment with OTX015 (■), everolimus (RAD001, ■) or the combination (■). Variations between the experimental groups were evaluated by a one-way ANOVA test (*p* < 0.001) followed by an SNK *a posteriori* test. Letters above the bars indicate significant differences (*p* < 0.05) in mRNA levels among the experimental groups. Bars and vertical lines represent the mean ± SEM, respectively (*n* = 4).

The antiproliferative activity of OTX015 and everolimus as single agents and in combination was compared using the MTT method in the three cell lines. Everolimus showed a less potent antiproliferative effect than OTX015 (*p* < 0.05) in all three cell lines after 72-h treatments (Supplementary Table S7). Moreover, the everolimus EC_50_ values for all three cell lines were ≈15 μM, independently of the specific PTEN status of each cell line (Table 1). Concomitant treatment with OTX015 and everolimus for 72 h in HCC1937 and MDA-MB-231 cells produced an additive effect (combination index = 1.02 and 0.94, respectively; Figure 5A). In contrast, this combination resulted in slight antagonism in the MDA-MB-468 cell line (combination index = 1.60).

### *In vivo* experiments in MDA-MB-231 xenografts

Based on *in vitro* combination studies, the MDA-MB-231 cell line was selected to generate murine xenografts to evaluate the *in vivo* effect of OTX015 alone and in combination with everolimus. OTX015-treated mice showed a substantial reduction in tumor mass with respect to the control group (*p* < 0.05) from 7 days after treatment start (Figure 5B). The best T/C value was 41.3% recorded on day 23 (Table 2). On the other hand, everolimus alone did not substantially affect tumor growth with respect to the control group.

**Table 2:** Efficacy of OTX015, everolimus and paclitaxel in MDA-MB-231 xenograft mice

Treatment group	Best T/C% (day of treatment)	AGD	LCK
**50 mg/kg OTX015**	**41.3 (23)**	10.1	0.41
**2 mg/kg RAD001**	58.6 (16)	3.6	0.15
**OTX015 + RAD001**	**20.7 (23)**	21.6	0.89
**0.15 mg/kg paclitaxel**	**28.9 (23)**	17.8	0.64

AGD, absolute growth delay (difference between the average times for each tumor to reach 500 mg in treated and control groups); LCK, log cell kill (*log*_2_ × AGD/tumor doubling time); T/C%, mean tumor mass in treated *versus* control animals (T/C < 42% is considered active [28, 29] – in bold), RAD001, everolimus.

In this experimental setting, the OTX015/everolimus combination was the most effective approach, showing a significant reduction in tumor mass with respect to vehicle-treated animals (*p* < 0.05) from day 4 after treatment start (Figure 5B) and a best T/C 20.7% on day 23 (Table 2). Furthermore, throughout the treatment period, mean tumor weights of the OTX015/everolimus combination group were significantly lower (*p* < 0.05) with respect to the everolimus and OTX015 single agents groups from day 7 and 21 onwards, respectively. More importantly, after treatment end, tumors of mice treated with the combination remained significantly smaller (*p* < 0.05) than those in animals treated with either of the single agents (Figure 5B). Of note, signs of toxicity were apparent in all mice in the everolimus-treated groups, as indicated by decreased mean body weight (Figure 5C). The everolimus schedule was therefore modified to once-a-week from day 14 after treatment start for both single agent and combination schedules.

Paclitaxel, which is widely used in the clinic to treat TNBC, was evaluated in a separate experiment, providing a clinical benchmark (Supplementary Figure S2). This drug showed activity with an optimal T/C of 28.9% on day 23 (Table 2). Comparison of the pharmacological efficacy of OTX015, everolimus, the OTX015/everolimus combination and paclitaxel according to three parameters (T/C%, AGD and LCK), demonstrated clearly that the OTX015/everolimus combination is the most active treatment (Table 2).

At the end of the treatment period, half of the animals from the OTX015 group were sacrificed 4 h after their last dose to determine drug levels in blood and tissues. Tumor and plasma presented equivalent OTX015 concentrations, which were significantly higher (*p* < 0.05) than the levels observed in peritumoral tissue (Figure 5D). These drug concentrations were ≈2 μM (in both plasma and tumor tissue), indicating that concentrations used *in vitro* are achievable in the tumor environment, which is coherent with a recent report from Gaudio *et al*. in lymphoma models [23]. For the remaining animals, as mentioned above, tumor and body weight were monitored for a further 20 days.

### Expression of c-MYC, BETs and CSC markers in MDA-MB-231 xenografts

Tumor tissue from xenograft mice sacrificed 4 weeks after treatment start was evaluated for changes in key genes (Figure 5E). None of the experimental groups showed variations in the expression of *c-MYC*, *BRD2*/*3* mRNA with respect to the vehicle-treated animals. Nevertheless, mice treated with the OTX015/everolimus combination showed a significant reduction (*p* < 0.05) in mRNA levels of *BRD4* in comparison with the vehicle control group. Likewise, as in the *in vitro* case, OTX015 induced a substantial increase in *CD24* mRNA levels with concomitant reduction in *CD44* expression (*p* < 0.05). However, the OTX015/everolimus combination resulted in only a marked increase (*p* < 0.05) in *CD24*, without affecting *CD44* expression. On the other hand, everolimus induced a significant decrease (*p* < 0.05) in *CD24* expression with no effect on *CD44*. Both OTX015 and the combination treatment down-regulated mRNA levels of the *EpCAM*, *NANOG* and *OCT4* stemness markers (*p* < 0.05). In contrast, everolimus did not modify the *EpCAM* and *OCT4* expression but significantly up-regulated *NANOG* mRNA levels (*p* < 0.05).

## DISCUSSION

Clinical management of TNBC remains a major concern, with very few therapeutic options for this specific population. No targeted therapies have been successfully developed against this malignancy to date, mainly due to the challenging intra- and inter-tumor heterogeneity that characterizes TNBC [10]. Taking into account the fact that triple-negative breast tumors are considered to be driven by *c-MYC* [4] and that BET inhibitors OTX015 and JQ1 have been reported to induce *c-MYC* down-regulation in several cancer types [14, 15, 17, 30, 31], we hypothesized that OTX015 would exert antitumor effects in TNBC cell models by down-regulating *c-MYC* expression. However, our findings indicate that, even when OTX015 displayed an equivalent inhibitory effect on the cell growth of the three cell lines evaluated, only the MDA-MB-468 cells showed decreased expression levels of *c-MYC*. Nevertheless, GEP studies concurrently indicated a significant down-regulation in MYC-regulated set of genes in both MDA-MB-231 and MDA-MB-468 cells. In light of this, we cannot rule out the possibility that OTX015 affects the transcriptional activity of c-MYC in these cell models in a similar manner, leading to the same final effect. Further studies will corroborate or discredit this hypothesis.

Evidence points to the PI3K/Akt/mTOR cascade as a potential therapeutic target in TNBC [32]. However, the use of inhibitors of this pathway as single-agent therapies has proved minimally effective in breast cancer [10]. Hurvitz *et al*. indicate that there is no association between PTEN expression levels and everolimus antiproliferative effect [33], which is in agreement with results presented here. The everolimus/exemestane combination was approved in more than 100 countries for patients with advanced hormone receptor-positive HER2-negative breast cancers, based on the Breast Cancer Trials of Oral Everolimus-2 (BOLERO-2) phase III trial [34, 35]. Tellingly, the OTX015/everolimus combination was the most effective approach in MDA-MB-231 xenografts. It is well known that targeting specific signaling pathways crucial for tumor growth, such as PI3K/AKT/mTOR, tyrosine kinase receptors and MAPK/ERK, elicits adaptive kinome and transcriptome responses which trigger a global reorganization of cell signaling machinery in order to up-regulate alternative kinase signaling networks or reactivate the targeted pathway to overcome the repressive treatment [36]. Based on this, Stuhlmiller *et al*. have suggested that targeting broad-acting epigenetic regulators of transcription such as BET proteins is not only advantageous, but also necessary to attenuate this dramatic induction of gene expression [36]. Bihani *et al*. agree with this therapeutic approach, recently reporting that BRD4-induced up-regulation of c-MYC is crucial in mediating everolimus resistance in different models of estrogen receptor-positive breast cancer cells [37]. Moreover, they showed that the JQ1/everolimus combination was highly effective at inhibiting the growth of resistant cells. A very recent study by Shu *et al*. indicates that the JQ1 resistance developed by TNBC cell lines may be based on epigenetic mechanisms of adaptation, specifically, the gain of BRD4-targeted super-enhancers accompanied by increased levels of phosphorylated BRD4 due to a reduced PP2A phosphatase activity [22]. This fact supports the OTX015/everolimus approach since this enzyme is also an Akt pathway inhibitor [38]. However, further studies are required to fully understand the role of OTX015 and mTOR inhibitors in TNBC, given some of our seemingly inconsistent results; OTX015 treatment induced down-regulation of *c-MYC* expression in only MDA-MB-468 cells, where the combination of OTX015 with everolimus resulted in an antagonistic interaction. Other combination alternatives are currently being evaluated in our laboratories. Nevertheless, it will be important to accompany these experiments with the identification of specific biomarkers of the different TNBC types.

Interestingly, in lymphoma models, high expression of hypoxia-related genes was reported to be associated with high sensitivity to OTX015 [14]. In this work, OTX015 appears to have induced a pro-anaerobic metabolism profile in TNBC cells, as inferred by the increased expression of glycolysis-inducing genes to the detriment of key genes necessary for mitochondrial activity. Importantly, this effect was not advantageous to OTX015-treated cells in hypoxia.

There is increasing evidence that CSCs, which are found in many human solid tumors such as breast, liver, colon and prostate, are involved in many aspects of human cancer development, from initiation, tumorigenesis, metastasis, angiogenesis and recurrence, impacting the efficacy of standard chemotherapy treatments [39, 40]. It has been suggested that the failure of cytotoxic chemotherapeutics such as taxanes and cisplatin in TNBC patients and ovarian cancer-derived cells may be due to the induction of CSC generation [41, 42]. As shown herein, OTX015 down-regulated the expression of genes specifically associated with stemness processes such as *NANOG* and *OCT4*, both *in vitro* and *in vivo*. Substantial evidence indicates that BET proteins may play an important role in stemness induction and maintenance. Horne *et al*. recently reported that JQ1 affects the pluripotency of murine embryonic stem cells by downregulating *NANOG* expression [42]. Of note, this effect was not accompanied by down-regulation of c-MYC levels. In addition, Shi *et al*. have described that *WNT5A* is a crucial activator of CSC-like properties and invasion in basal-like breast cancer, and that BRD4/diacetylated-Twist binding at the *WNT5A* promoter is necessary for its expression [43]. Indeed, JQ1-mediated blockade of this interaction suppressed tumorigenicity and stemness properties in these cells. In addition, Herrmann *et al*. have described the antiproliferative and pro-apoptotic effects of JQ1 in patient-derived acute myeloid leukemia cells, particularly affecting leukemia stem and progenitor niches [44]. Together, these findings point to BET proteins as promising drug targets to address the CSC component of tumorigenesis in different malignancies.

At least three other small molecule BET inhibitors are currently being evaluated in clinical trials in a range of tumor types (NCT01949883, NCT01587703, NCT01987362), highlighting the increasing interest in this class of drugs. Our pre-clinical results show the therapeutic potential of OTX015 both as a single agent as well as in combination with everolimus for the treatment of TNBC.

## MATERIALS AND METHODS

### Reagents

Reagents were obtained from Sigma-Aldrich (USA), unless otherwise specified. OTX015 was supplied by Oncoethix GmbH (a wholly owned subsidiary of Merck Sharp & Dohme Corp., Switzerland). For *in vitro* experiments, it was dissolved in DMSO (10 mM) and stored at −20°C. For *in vivo* assays, the OTX015-SDI formulation was stored at room temperature and prepared by adding water immediately before administration. Everolimus was purchased from SelleckChem (USA; cat. S1120). For *in vitro* assays, it was dissolved in DMSO (50 mM) and stored at −20°C. For *in vivo* studies, a stock solution was prepared in ethanol (5.5 mg/ml) and stored at −20°C. Before administration, a 0.2 mg/ml solution was prepared with vehicle: 5% Tween-80 + 5% polyethylene glycol 400.

### Cell lines

The HCC1937, MDA-MB-231 and MDA-MB-468 cell lines were obtained from the American Type Culture Collection (ATCC; USA). All three cell lines were authenticated by Idexx Bioresearch (Germany) with a CellCheck 9 Plus analysis in February 2014. The HCC1937 cell line was cultured in RPMI 1640 medium, while MDA-MB-231 and MDA-MB-468 cells were grown in DMEM. Media (Lonza, Belgium) were supplemented with 10% FBS, 2 mM glutamine (Lonza), and 1% penicillin-streptomycin (Biowest, France), and cells were cultured at 37°C in a humidified atmosphere with 5% CO_2_. Hypoxia experiments were carried out at 37°C in a humidified atmosphere with 0.1% O_2_ and 5% CO_2_ in an InvivO2 400 Hypoxia Workstation^®^ (Ruskinn Technology, UK). Cell lines were regularly checked for *Mycoplasma* *spp*. infection by PCR throughout the study.

### Cell counting

Cells (20000/ml) were seeded in a 12-well plate (1 ml/well) under normoxic or hypoxic conditions and treated 24 h later. After 72-h treatments, cells were detached with trypsin-EDTA and their number determined with a Beckman Multisizer^®^ 3 Coulter Counter^®^ (Beckman Coulter, USA). Wells with 0.1% DMSO were used as vehicle control, confirming cell proliferation was not affected (data not shown). The drug concentration that gives a half-maximal effect (EC_50_) with the 95% confidence interval (CI95%) and the maximum effect of the drug (E_max_) were calculated using the equation for sigmoidal dose response, employing Prism 5.00 software (GraphPad Software, USA). GI_50_ values were defined as the concentration at which cell growth/proliferation was reduced by half.

### Drug combination studies

Cells were seeded at a density of 20000 cells/ml in 96-well plates (100 μl/wells) and treated 24 h later with different concentrations of OTX015 or everolimus, or combination of both drugs for 72 h. Cells were then incubated with 0.8 mg/ml MTT (3-[4,5-dimethylthiazol-2-yl]-2,5-diphenyltetrazolium bromide) for 2–4 hours. Cell pellets were resuspended in 0.05 ml DMSO and absorbance was measured at 560 nm using an Infinte 200 microplate reader (Tecan Trading AG, Switzerland). OTX015 and everolimus EC_50_ values were determined as previously mentioned using Prism 5.00 software. Assays were performed in triplicate in at least three independent experiments. Results were further analyzed according to the Chou-Talalay algorithm with Compusyn software [45]. Based on the calculated combination indexes values, < 0.90 reflects synergism, 0.9 to ≤ 1.10 indicates additive effects, and > 1.10 reflects antagonism.

### Cell cycle analysis

Control and treated cells were detached with trypsin-EDTA solution, fixed, stained and processed by using FACS Calibur instrument (Becton Dickinson, USA), as previously described [46].

### Western blotting

Cells were seeded in T75 flasks (10000–50000 cells/ml) and treated 24 h later with OTX015 GI_50_ concentrations for 24, 48 and 72 h. Untreated cells were used as controls. Cells were lysed in 50 mM Tris–HCl pH 6.8, 2% SDS, 100 mM 2-mercaptoethanol, 10% glycerol and 0.05% bromophenol blue, and sonicated to shear DNA. Cell lysates (10 μg) were resolved by SDS–PAGE, and probed with the anti-BRD2 (cat. ab139690, Abcam, UK), anti-BRD3 (cat. ab56342, Abcam), anti-BRD4 (cat. sc-48772, USA) and anti-β-tubulin (cat. sc-9104, Santa Cruz) primary antibodies. All blots were incubated with horseradish peroxidase conjugated anti-rabbit or anti-mouse secondary antibodies (cat. sc-2004 and sc-2005, respectively; Santa Cruz) and developed by enhanced chemiluminescence (Thermo Scientific, USA; cat. 34080).

### Quantitative real-time PCR (qRT-PCR)

RNA was extracted with the RNeasy Mini Kit (Qiagen, Germany; cat. 74104) following manufacturer's instructions. Tumor tissue was broken down mechanically before RNA extraction. Samples were processed and analyzed as previously described [16]. Primers used for qRT-PCR were designed and validated in our laboratory (Supplementary Table S8). Results are expressed as n-fold differences in the target gene expression relative to the housekeeping genes (*GAPDH* and *HPRT1*) [47].

### Gene expression profiling

RNA was extracted as described above and hybridized on HumanHT-12-v4 Expression BeadChip (Illumina^®^, USA) microarrays. Gene expression values were processed and analyzed as previously reported [14]. Changes in expression level were considered statistically significant when the absolute value of logarithm of fold-change (|LogFC|) was ≥ 0.5 and the adjusted *p*-value (*q*) was ≤ 0.05. Functional gene set analysis was performed using gene set enrichment analysis (GSEA) with the complete MSigDB v5.0 database and drug signatures created from the Connectivity Map database (CMAP). In addition, gene set expression values were computed performing a ‘gene set variation analysis’ (GSVA). In these studies, a *p*-value ≤ 0.05 and a false discovery rate (FDR) < 25% were considered statistically significant. Raw data will be available at the National Center for Biotechnology Information Gene Expression Omnibus (https://www.ncbi.nlm.nih.gov/geo/) database. Access code GSE79721.

### Animal studies

6-week-old female nude Foxn1 mice (≈25 g, Harlan Laboratories, Italy) were maintained at a constant temperature and humidity, according to institutional guidelines. Protocols were approved by the Ethics Committee of the IRCCS-Istituto di Ricerche Farmacologiche Mario Negri (Italy), in compliance with national (D.lgs 26/2014; Authorisation n.19/2008-A issued March 6, 2008 by Ministry of Health) and international laws (EU Directive 2010/63/EU).

Mice were subcutaneously injected in the right flank with 10 × 10^6^ MDA-MB-231 cells. When average tumor weight was ≈130 mg, mice were randomized (nine animals/group) to one of the following experimental groups: vehicle (for OTX15, water, twice daily, oral; for everolimus vehicle, 5% Tween-80/5% polyethylene glycol 400, thrice weekly, intraperitoneal); 50 mg/kg OTX015, twice daily, oral; 2 mg/kg everolimus, thrice weekly, intraperitoneal; 50 mg/kg OTX015 + 2 mg/kg everolimus, according to the single agent dosing schedules. In the experiment with paclitaxel, mice were randomized (eight animals/group) to vehicle (cremophor:ethanol 1:1, then diluted 1:5 with saline; once weekly, intravenous) or 0.15 mg/kg paclitaxel, once weekly, intravenous. Mice were sacrified at the first sign of severe distress and tumors were collected. Tumor weight (1 mm^3^ = 1 mg) was determined using the formula *d^2^* × *D/2*, where ‘*d*’ and ‘*D*’ are the minor and major diameters of the tumor in mm, respectively. Growth curves of each tumor were normalized with respect to volume at the start of treatment. The doubling time (Td) of each untreated tumor was calculated by exponential fit of the whole growth curve. Treatment efficacy was evaluated based on T/C%, absolute growth delay (AGD) and log cell kill (LCK) parameters. In T/C%, T and C are the mean tumor weight of treated and control groups, respectively. A T/C < 42% was considered active, according to the standards of the National Cancer Institute (NCI) of the United States [28, 29]. AGD was calculated as the difference between the average times required for each tumor to reach 500 mg in treated and control groups. LCK was calculated with the formula *log* (2) × AGD/*Td*.

Tolerability was evaluated on the basis of body weight loss, clinical observation and mortality.

### OTX015 quantification in plasma and solid tissues

Mice were euthanized with CO_2_. Blood was collected by cardiac puncture and immediately heparinized. Plasma was separated by centrifugation at 2000 × g for 15 min at 4°C and stored at −80°C. Tumors and peritumoral tissues were rapidly frozen in dry ice and stored at −80°C. OTX015 concentrations were determined using a validated Acquity Ultra Performance Liquid Chromatography System (Waters, USA) coupled with a tandem mass spectrometry detection method (UPLC/MS/MS), as previously described [48].

### Statistical analyses

Graphical results are plotted as mean ± standard error of the mean (SEM) of at least three independent experiments. Statistical analysis was performed by one-way ANOVA followed by a Student–Newman–Keuls (SNK) *a posteriori* test, Student *t*-test for independent samples with equal or unequal variances, as appropriate, with InfoStat software (Argentina), or by two-way ANOVA followed by a Bonferroni *a posteriori* test employing Prism 5.0 software. Transformation of the variable to obtain homoscedasticity was applied before the ANOVA tests if required. A *p*-value ≤ 0.05 was considered statistically significant.

## SUPPLEMENTARY MATERIALS FIGURES AND TABLES








