# Supplementary material for: The bromodomain inhibitor OTX015 (MK-8628) exerts anti-tumor activity in triple-negative breast cancer models as single agent and in combination with everolimus
Source: Oncotarget. 2016 Dec 7;8(5):7598–613. doi: 10.18632/oncotarget.13814 (PMC5352346; doi:10.18632/oncotarget.13814)
Supplement: Supplementary file 1 [file oncotarget-08-7598-s001.pdf]

# The bromodomain inhibitor OTX015 (MK-8628) exerts anti-tumor activity in triple-negative breast cancer models as single agent and in combination with everolimus

## Supplementary Materials

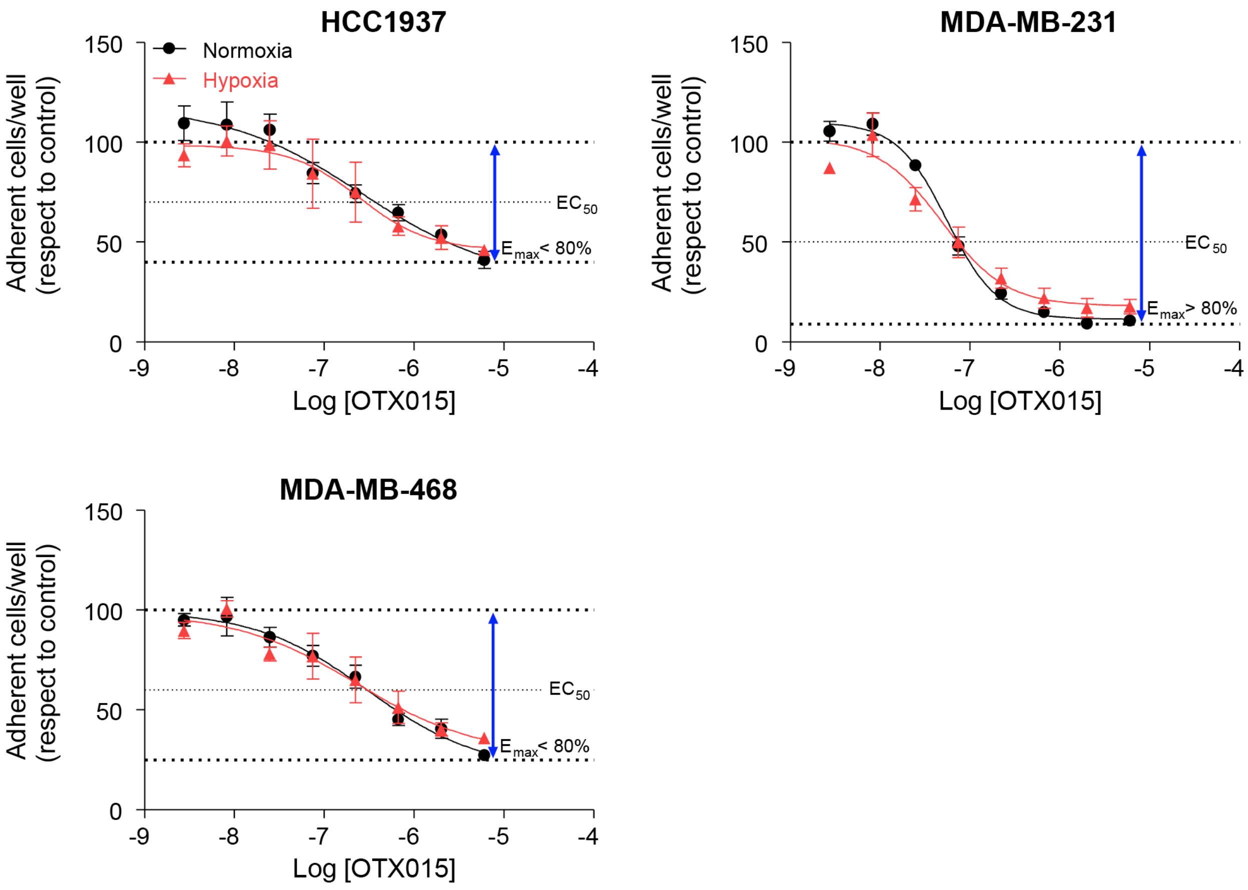


**Supplementary Figure S1: Cell growth inhibition curves under normoxic and hypoxic conditions, by cell count.** Cells were treated with increasing concentrations of OTX015 (2.7–6000 nM) or 0.1% (v/v) DMSO (vehicle control group, not shown) in normoxia and hypoxia. The effects on cell proliferation were evaluated at 72 h employing a Beckman Coulter cell counter. The dotted lines indicate the $E_{max}$ values. The $EC_{50}$ values were calculated with the equation for sigmoidal dose response using Prism 5.00 for MS Windows software. Each point and vertical line represents the mean ± SEM ($n \geq 3$).

### Evaluation of paclitaxel in MDA-MB-231 cell line-derived xenografts

6-week-old female nude Foxn1 mice (≈25 g, Harlan Laboratories, Italy) were maintained at a constant temperature and humidity, according to institutional guidelines. Protocols were approved by the Ethics Committee of the IRCCS-Istituto di Ricerche Farmacologiche Mario Negri (Italy), in compliance with national (D.lgs 26/2014; Authorisation n.19/2008-A issued March 6, 2008 by Ministry of Health) and international laws (EU Directive 2010/63/EU).

Mice were subcutaneously injected in the right flank with 10 × $10^6$ MDA-MB-231 cells and 12 days after randomized (eight animals/group) into vehicle (cremophor:ethanol 1:1 was used to dissolve paclitaxel, then diluted 1:5 with saline; once weekly, intravenous) or 0.15 mg/kg paclitaxel, once weekly, intravenous. Tumor weight and treatment efficacy were evaluated as described in the Material and Methods section. Tolerability was determined on the basis of body weight loss, clinical observation and mortality. Mice were sacrificed at the first sign of severe distress.

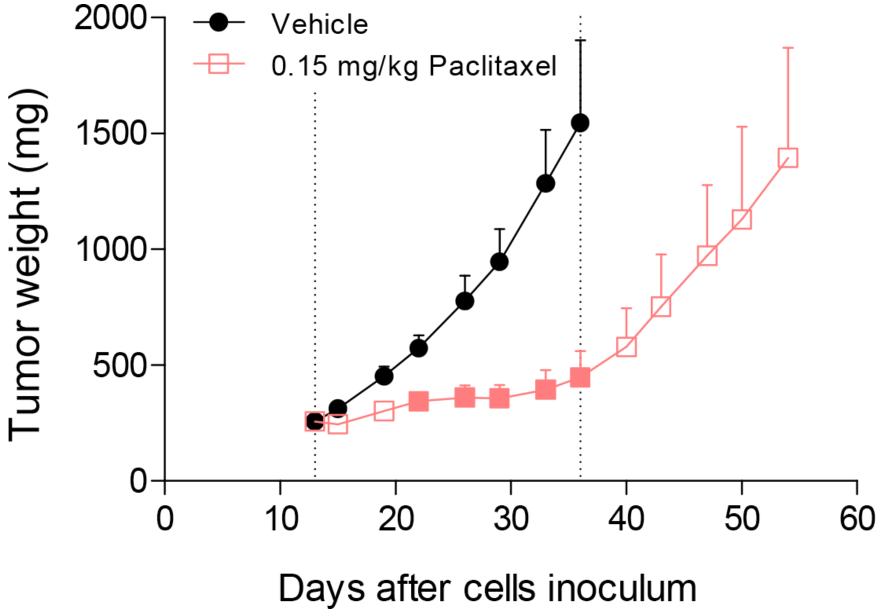


**Supplementary Figure S2: Anti-tumor effect of paclitaxel in MDA-MB-231 xenografts.** Tumor weight was compared between drug regimens at each time point using a two-way ANOVA test ($p$ < 0.001) followed by Bonferroni a posteriori test on log-transformed data. Color-filled symbols indicate significant ($p$ < 0.01) differences between treated vs. vehicle mice. Dotted vertical lines indicate the treatment period ($n$ = 8).

**Supplementary Table S1: LIMMA results in MDA-MB-231 cells.** See Supplementary_Table_S1

**Supplementary Table S2: LIMMA results in MDA-MB-468 cells.** See Supplementary_Table_S2

**Supplementary Table S3: Common up-regulated genes in MDA-MB-231 and MDA-MB-468 cell lines.** See Supplementary_Table_S3

**Supplementary Table S4: Common down-regulated in MDA-MB-231 and MDA-MB-468 cell lines**

| Symbol | Definition | Synonyms |
|---|---|---|
| GPR56 | Homo sapiens G protein-coupled receptor 56 (GPR56), transcript variant 3, mRNA. | BFPP; DKFZp781L1398; TM7LN4; TM7XN1 |
| FJX1 | Homo sapiens four jointed box 1 (Drosophila) (FJX1), mRNA. | FLJ25593; FLJ22416 |
| PHF15 | Homo sapiens PHD finger protein 15 (PHF15), mRNA. | JADE2; KIAA0239 |
| SPDEF | Homo sapiens SAM pointed domain containing ets transcription factor (SPDEF), mRNA. | bA375E1.3; PDEF; RP11-375E1__A.3 |
| CCDC86 | Homo sapiens coiled-coil domain containing 86 (CCDC86), mRNA. | FLJ22321; MGC2574 |
| TRIP6 | Homo sapiens thyroid hormone receptor interactor 6 (TRIP6), mRNA. | MGC29959; MGC3837; MGC10556; OIP1; ZRP-1; MGC4423; MGC10558 |

**Supplementary Table S5: MSIGDB GSEA report.** See Supplementary_Table_S5

**Supplementary Table S6: CMAP GSEA report.** See Supplementary_Table_S6

**Supplementary Table S7: OTX015 and Everolimus $EC_{50}$ values at 72 h determined by the MTT assay**

| Cell line | OTX015 | | Everolimus | |
|---|---|---|---|---|
| | $EC_{50}$ (nM) | $E_{max}$% | $EC_{50}$ (μM) | $E_{max}$% |
| **HCC1937** | 81.3 (47.0–140.5) | 51 | 13.2 (12.0–14.5) | > 95 |
| **MDA-MB-231** | 81.7 (67.2–99.4) | 83 | 14.8 (12.2–18) | > 95 |
| **MDA-MB-468** | 448.3 (269.2–746.5) | 42 | 17.5 (12.8–19.6) | > 95 |

Results are expressed as the mean concentration that that gives half-maximal response ($EC_{50}$) with 95% confidence intervals. $E_{max}$ % indicates the maximum inhibitory effect induced by OTX015 on cell proliferation (with respect to control untreated cells). In all cases, $n \geq 3$.

**Supplementary Table S8: Primer sequences (forward and reverse) used for real-time PCR**

| Gene | Primer | Sequence | Primer length | $T_m$ (°C) | Amplicon length | Comments |
|---|---|---|---|---|---|---|
| ***c-MYC* (NM_002467)** | Fw<br>Rv | cgactctgaggaggaacaagaa<br>ggatagtccttccgagtgga | 22<br>20 | 60.9<br>59.1 | 210 | Primer not spanning an exon-exon junction |
| ***BRD2* 4 isoforms** | Fw<br>Rv | ccacctcaacctaagaagtcca<br>acttcctccagaaggtccaaag | 22<br>22 | 60.5<br>61.0 | 93 | Specific to 4 isoforms<br>Not spanning an exon-exon junction |
| ***BRD3* (NM_005378.4)** | Fw<br>Rv | gtgcacatcatccaatctcg<br>cttgctgagaacggtttcct | 20<br>20 | 60.1<br>59.5 | 158 | Primer spanning an exon-exon junction |
| ***BRD4* 2 isoforms** | Fw<br>Rv | tctacaacaagcctggagatga<br>tctcggtttcttctgtgggtag | 22<br>22 | 59.9<br>60.7 | 99 | Specific to 2 isoforms<br>Primer spanning an exon-exon junction |
| ***n-MYC* (NM_005378.4)** | Fw<br>Rv | tgagcgattcagatgatgaaga<br>gcatcgtttgaggatcagc | 22<br>19 | 60.9<br>59.3 | 191 | Primer spanning an exon-exon junction |
| ***p21* 2 isoforms** | Fw<br>Rv | gaccagcatgacagatttctacc<br>aagatgtagagcgggccttt | 23<br>20 | 60.0<br>60.2 | 127 | Specific to 2 isoforms<br>Not spanning an exon-exon junction |
| ***GAPDH* 2 isoforms** | Fw<br>Rv | gatccctccaaaatcaagtgg<br>ggaggcattgctgatgatct | 21<br>20 | 60.7<br>60.2 | 213 | Specific to 2 isoforms<br>Primer spanning an exon-exon junction |
| ***HPRT1* (NM_0000194.2)** | Fw<br>Rv | tgaatacttcagggatttgaatcat<br>ctcatcttaggctttgtattttgc | 25<br>24 | 60.0<br>60.0 | 76 | Primer spanning an exon-exon junction |
| ***β-actin* (NM_001101.3)** | Fw<br>Rv | cagagcctcgcctttgc<br>tcatcatccatggtgagctg | 17<br>20 | 60.1<br>58.6 | 76 | Primer spanning an exon-exon junction |
| ***EpCAM* (NM_002354.2)** | Fw<br>Rv | ttgtggttgtggtgatagcag<br>cacccatctcctttatctcagc | 21<br>22 | 59.6<br>60.1 | 105 | Primer spanning an exon-exon junction |
| ***Musashi-1* (NM_002442.3)** | Fw<br>Rv | tgttcatcgggggactca<br>ggtcaattgttttggagtcgag | 18<br>22 | 61<br>60.8 | 210 | Primer spanning an exon-exon junction |
| ***NANOG* 2 isoforms** | Fw<br>Rv | gccttgctttgaagcatcc<br>gaggaaggaagaggagagacagt | 19<br>23 | 59.1<br>60.0 | 138 | Specific to 2 isoforms<br>Primer spanning an exon-exon junction |
| ***OCT4* 5 isoforms** | Fw<br>Rv | cacacaaagcactttatccattct<br>tcacccccagtttaaggatgt | 24<br>21 | 59.6<br>60.6 | 220 | Specific to 4 isoforms<br>Not spanning an exon-exon junction |
| ***CD24* 4 isoforms** | Fw<br>Rv | tcctcccagagtacttccaact<br>gagtgagaccacgaagagactg | 22<br>22 | 59.2<br>59.1 | 111 | Specific to 4 isoforms<br>Not spanning an exon-exon junction |
| ***CD133* 7 isoforms** | Fw<br>Rv | cagctacttggctcagactgg<br>gtgcatctcttttcagggagtt | 21<br>22 | 60.2<br>59.8 | 128 | Specific to 7 isoforms<br>Not spanning an exon-exon junction |
| ***CD44* 8 isoforms** | Fw<br>Rv | ccctccgtcttaggtcactg<br>cggcaggttatattcaaatcg | 20<br>21 | 59.7<br>59.5 | 282 | Specific to 8 isoforms<br>Not spanning an exon-exon junction |
