# Supplementary material for: The bromodomain inhibitor OTX015 (MK-8628) exerts anti-tumor activity in triple-negative breast cancer models as single agent and in combination with everolimus
Source: Oncotarget. 2016 Dec 7;8(5):7598–613. doi: 10.18632/oncotarget.13814 (PMC5352346; doi:10.18632/oncotarget.13814)
Supplement: Supplementary file 2 [file oncotarget-08-7598-s002.docx]

**Supplementary Table S1.** LIMMA results in MDA-MB-231 cells.

| SEARCH_KEY | ILMN_GENE | CHROMOSOME | DEFINITION |
| --- | --- | --- | --- |
| NM_138720.1 | HIST1H2BD | 6 | Homo sapiens histone cluster 1, H2bd (HIST1H2BD), transcript variant 2, mRNA. |
| NM_138720.1 | HIST1H2BD | 6 | Homo sapiens histone cluster 1, H2bd (HIST1H2BD), transcript variant 2, mRNA. |
| NM_003512.3 | HIST1H2AC | 6 | Homo sapiens histone cluster 1, H2ac (HIST1H2AC), mRNA. |
| NM_078467.1 | CDKN1A | 6 | Homo sapiens cyclin-dependent kinase inhibitor 1A (p21, Cip1) (CDKN1A), transcript variant 1, mRNA. |
| NM_005020.1 | PDE1C | 7 | Homo sapiens phosphodiesterase 1C, calmodulin-dependent 70kDa (PDE1C), mRNA. |
| NM_013409.1 | FST | 5 | Homo sapiens follistatin (FST), transcript variant FST344, mRNA. |
| NM_000142.2 | FGFR3 | 4 | Homo sapiens fibroblast growth factor receptor 3 (achondroplasia, thanatophoric dwarfism) (FGFR3), transcript variant 2, mRNA. |
| NM_006086.2 | TUBB3 | 16 | Homo sapiens tubulin, beta 3 (TUBB3), mRNA. |
| NM_017579.1 | DMBT1 | 10 | Homo sapiens deleted in malignant brain tumors 1 (DMBT1), transcript variant 3, mRNA. |
| NM_017445.1 | HIST1H2BK | 6 | Homo sapiens histone cluster 1, H2bk (HIST1H2BK), mRNA. |
| NM_003516.2 | HIST2H2AA3 | 1 | Homo sapiens histone cluster 2, H2aa3 (HIST2H2AA3), mRNA. |
| NM_033103.3 | RHPN2 | 19 | Homo sapiens rhophilin, Rho GTPase binding protein 2 (RHPN2), mRNA. |
| NM_005328.1 | HAS2 | 8 | Homo sapiens hyaluronan synthase 2 (HAS2), mRNA. |
| Hs.4892 | HS.4892 | 2 | Homo sapiens clone 24841 mRNA sequence |
| NM_005952.2 | MT1X | 16 | Homo sapiens metallothionein 1X (MT1X), mRNA. |
| NM_005318.2 | H1F0 | 22 | Homo sapiens H1 histone family, member 0 (H1F0), mRNA. |
| NM_152644.2 | FAM24B | 10 | Homo sapiens family with sequence similarity 24, member B (FAM24B), mRNA. |
| NM_003543.3 | HIST1H4H | 6 | Homo sapiens histone cluster 1, H4h (HIST1H4H), mRNA. |
| XM_933997.1 | LOC284023 |  | PREDICTED: Homo sapiens hypothetical protein LOC284023, transcript variant 3 (LOC284023), mRNA. |
| NM_003528.2 | HIST2H2BE | 1 | Homo sapiens histone cluster 2, H2be (HIST2H2BE), mRNA. |
| NM_001040874.1 | HIST2H2AA4 | 1 | Homo sapiens histone cluster 2, H2aa4 (HIST2H2AA4), mRNA. |
| NM_000266.1 | NDP | X | Homo sapiens Norrie disease (pseudoglioma) (NDP), mRNA. |
| NM_080593.1 | HIST1H2BK | 6 | Homo sapiens histone cluster 1, H2bk (HIST1H2BK), mRNA. |
| XM_001718050.1 | LOC100134073 |  | PREDICTED: Homo sapiens similar to LYPDC1 protein (LOC100134073), mRNA. |
| NM_004030.1 | IRF7 | 11 | Homo sapiens interferon regulatory factor 7 (IRF7), transcript variant b, mRNA. |
| NM_003877.3 | SOCS2 | 12 | Homo sapiens suppressor of cytokine signaling 2 (SOCS2), mRNA. |
| NM_002924.2 | RGS7 |  | Homo sapiens regulator of G-protein signalling 7 (RGS7), mRNA. |
| NM_020127.1 | TUFT1 | 1 | Homo sapiens tuftelin 1 (TUFT1), mRNA. |
| NM_203418.1 | RCAN1 | 21 | Homo sapiens regulator of calcineurin 1 (RCAN1), transcript variant 3, mRNA. |
| NM_175621.2 | MTE |  | Homo sapiens metallothionein E (MTE), mRNA. |
| NM_006362.4 | NXF1 | 11 | Homo sapiens nuclear RNA export factor 1 (NXF1), transcript variant 1, mRNA. |
| NM_138440.1 | VASN | 16 | Homo sapiens vasorin (VASN), mRNA. |
| NM_000584.2 | IL8 | 4 | Homo sapiens interleukin 8 (IL8), mRNA. |
| Hs.537002 | HS.537002 | X | Homo sapiens cDNA clone IMAGE:4811759 |
| NM_148957.2 | TNFRSF19 | 13 | Homo sapiens tumor necrosis factor receptor superfamily, member 19 (TNFRSF19), transcript variant 2, mRNA. |
| NM_003485.3 | GPR68 | 14 | Homo sapiens G protein-coupled receptor 68 (GPR68), mRNA. |
| NM_016286.2 | DCXR | 17 | Homo sapiens dicarbonyl/L-xylulose reductase (DCXR), mRNA. |
| NM_013961.1 | NRG1 | 8 | Homo sapiens neuregulin 1 (NRG1), transcript variant GGF2, mRNA. |
| NM_000576.2 | IL1B | 2 | Homo sapiens interleukin 1, beta (IL1B), mRNA. |
| NM_201525.1 | GPR56 | 16 | Homo sapiens G protein-coupled receptor 56 (GPR56), transcript variant 3, mRNA. |
| NM_005104.2 | BRD2 | 6 | Homo sapiens bromodomain containing 2 (BRD2), mRNA. |
| NM_018645.3 | HES6 | 2 | Homo sapiens hairy and enhancer of split 6 (Drosophila) (HES6), mRNA. |
| XM_927769.1 | LOC653506 | 17 | PREDICTED: Homo sapiens similar to meteorin, glial cell differentiation regulator-like (LOC653506), mRNA. |
| NM_005644.2 | TAF12 | 1 | Homo sapiens TAF12 RNA polymerase II, TATA box binding protein (TBP)-associated factor, 20kDa (TAF12), mRNA. |
| NM_013376.1 | SERTAD1 | 19 | Homo sapiens SERTA domain containing 1 (SERTAD1), mRNA. |
| NM_005950.1 | MT1G | 16 | Homo sapiens metallothionein 1G (MT1G), mRNA. |
| NM_021058.3 | HIST1H2BJ | 6 | Homo sapiens histone cluster 1, H2bj (HIST1H2BJ), mRNA. |
| NM_023925.3 | CAPRIN2 | 12 | Homo sapiens caprin family member 2 (CAPRIN2), transcript variant 1, mRNA. |
| NM_005025.2 | SERPINI1 | 3 | Homo sapiens serpin peptidase inhibitor, clade I (neuroserpin), member 1 (SERPINI1), mRNA. |
| NM_175617.3 | MT1E | 16 | Homo sapiens metallothionein 1E (MT1E), mRNA. |
| NM_001814.2 | CTSC | 11 | Homo sapiens cathepsin C (CTSC), transcript variant 1, mRNA. |
| NM_001007538.1 | SHISA2 | 13 | Homo sapiens shisa homolog 2 (Xenopus laevis) (SHISA2), mRNA. |
| NM_005319.3 | HIST1H1C | 6 | Homo sapiens histone cluster 1, H1c (HIST1H1C), mRNA. |
| NM_014344.2 | FJX1 | 11 | Homo sapiens four jointed box 1 (Drosophila) (FJX1), mRNA. |
| NM_001011668.1 | CHCHD7 | 8 | Homo sapiens coiled-coil-helix-coiled-coil-helix domain containing 7 (CHCHD7), transcript variant 6, mRNA. |
| NM_020040.3 | TUBB4Q | 4 | Homo sapiens tubulin, beta polypeptide 4, member Q (TUBB4Q), mRNA. |
| NM_007084.2 | SOX21 | 13 | Homo sapiens SRY (sex determining region Y)-box 21 (SOX21), mRNA. |
| NM_003517.2 | HIST2H2AC | 1 | Homo sapiens histone cluster 2, H2ac (HIST2H2AC), mRNA. |
| NM_006472.1 | TXNIP | 1 | Homo sapiens thioredoxin interacting protein (TXNIP), mRNA. |
| NM_197941.2 | ADAMTS6 | 5 | Homo sapiens ADAM metallopeptidase with thrombospondin type 1 motif, 6 (ADAMTS6), mRNA. |
| NM_020909.2 | EPB41L5 | 2 | Homo sapiens erythrocyte membrane protein band 4.1 like 5 (EPB41L5), mRNA. |
| XM_937100.1 | LOC728285 | 17 | PREDICTED: Homo sapiens similar to keratin associated protein 2-4 (LOC728285), mRNA. |
| NM_006096.2 | NDRG1 | 8 | Homo sapiens N-myc downstream regulated gene 1 (NDRG1), mRNA. |
| XM_938862.1 | LRRC58 |  | PREDICTED: Homo sapiens leucine rich repeat containing 58 (LRRC58), mRNA. |
| NM_181339.1 | IL24 | 1 | Homo sapiens interleukin 24 (IL24), transcript variant 1, mRNA. |
| NM_000362.4 | TIMP3 | 22 | Homo sapiens TIMP metallopeptidase inhibitor 3 (TIMP3), mRNA. |
| XM_498969.2 | LOC441019 | 4 | PREDICTED: Homo sapiens hypothetical LOC441019 (LOC441019), mRNA. |
| NM_015288.4 | PHF15 | 5 | Homo sapiens PHD finger protein 15 (PHF15), mRNA. |
| NM_012391.1 | SPDEF | 6 | Homo sapiens SAM pointed domain containing ets transcription factor (SPDEF), mRNA. |
| NM_000584.2 | IL8 | 4 | Homo sapiens interleukin 8 (IL8), mRNA. |
| NM_024098.1 | CCDC86 | 11 | Homo sapiens coiled-coil domain containing 86 (CCDC86), mRNA. |
| NM_006362.3 | NXF1 | 11 | Homo sapiens nuclear RNA export factor 1 (NXF1), transcript variant 2, mRNA. |
| NM_003516.2 | HIST2H2AA3 | 1 | Homo sapiens histone cluster 2, H2aa3 (HIST2H2AA3), mRNA. |
| NM_020808.1 | SIPA1L2 | 1 | Homo sapiens signal-induced proliferation-associated 1 like 2 (SIPA1L2), mRNA. |
| NM_152680.1 | TMEM154 | 4 | Homo sapiens transmembrane protein 154 (TMEM154), mRNA. |
| NM_023112.2 | OTUB2 | 14 | Homo sapiens OTU domain, ubiquitin aldehyde binding 2 (OTUB2), mRNA. |
| NM_020814.1 | Mar-04 | 2 | Homo sapiens membrane-associated ring finger (C3HC4) 4 (MARCH4), mRNA. |
| NM_144649.1 | TMEM71 | 8 | Homo sapiens transmembrane protein 71 (TMEM71), mRNA. |
| Hs.551128 | HS.551128 | 8 | Homo sapiens MSTP131 (MST131) mRNA, complete cds |
| NM_003302.1 | TRIP6 | 7 | Homo sapiens thyroid hormone receptor interactor 6 (TRIP6), mRNA. |
| NM_080757.1 | C20ORF127 | 20 | Homo sapiens chromosome 20 open reading frame 127 (C20orf127), mRNA. |

**Supplementary Table S2.** LIMMA results in MDA-MB-468 cells.

| SEARCH_KEY | ILMN_GENE | CHROMOSOME | DEFINITION |
| --- | --- | --- | --- |
| NM_003512.3 | HIST1H2AC | 6 | Homo sapiens histone cluster 1, H2ac (HIST1H2AC), mRNA. |
| NM_078467.1 | CDKN1A | 6 | Homo sapiens cyclin-dependent kinase inhibitor 1A (p21, Cip1) (CDKN1A), transcript variant 1, mRNA. |
| NM_138720.1 | HIST1H2BD | 6 | Homo sapiens histone cluster 1, H2bd (HIST1H2BD), transcript variant 2, mRNA. |
| NM_003088.2 | FSCN1 | 7 | Homo sapiens fascin homolog 1, actin-bundling protein (Strongylocentrotus purpuratus) (FSCN1), mRNA. |
| NM_006362.4 | NXF1 | 11 | Homo sapiens nuclear RNA export factor 1 (NXF1), transcript variant 1, mRNA. |
| NM_003516.2 | HIST2H2AA3 | 1 | Homo sapiens histone cluster 2, H2aa3 (HIST2H2AA3), mRNA. |
| NM_006086.2 | TUBB3 | 16 | Homo sapiens tubulin, beta 3 (TUBB3), mRNA. |
| NM_001033049.1 | ADARB1 | 21 | Homo sapiens adenosine deaminase, RNA-specific, B1 (RED1 homolog rat) (ADARB1), transcript variant 1, mRNA. |
| NM_021058.3 | HIST1H2BJ | 6 | Homo sapiens histone cluster 1, H2bj (HIST1H2BJ), mRNA. |
| NR_003286.1 | LOC100008588 |  | Homo sapiens 18S ribosomal RNA (LOC100008588), non-coding RNA. |
| NM_138720.1 | HIST1H2BD | 6 | Homo sapiens histone cluster 1, H2bd (HIST1H2BD), transcript variant 2, mRNA. |
| NM_001040874.1 | HIST2H2AA4 | 1 | Homo sapiens histone cluster 2, H2aa4 (HIST2H2AA4), mRNA. |
| NM_006362.3 | NXF1 | 11 | Homo sapiens nuclear RNA export factor 1 (NXF1), transcript variant 2, mRNA. |
| Hs.4892 | HS.4892 | 2 | Homo sapiens clone 24841 mRNA sequence |
| XM_937367.1 | IL7R |  | PREDICTED: Homo sapiens interleukin 7 receptor (IL7R), mRNA. |
| NM_003528.2 | HIST2H2BE | 1 | Homo sapiens histone cluster 2, H2be (HIST2H2BE), mRNA. |
| NM_004030.1 | IRF7 | 11 | Homo sapiens interferon regulatory factor 7 (IRF7), transcript variant b, mRNA. |
| NM_012391.1 | SPDEF | 6 | Homo sapiens SAM pointed domain containing ets transcription factor (SPDEF), mRNA. |
| NM_005130.3 | FGFBP1 | 4 | Homo sapiens fibroblast growth factor binding protein 1 (FGFBP1), mRNA. |
| NM_001124.1 | ADM | 11 | Homo sapiens adrenomedullin (ADM), mRNA. |
| NM_003508.2 | FZD9 | 7 | Homo sapiens frizzled homolog 9 (Drosophila) (FZD9), mRNA. |
| NM_000142.2 | FGFR3 | 4 | Homo sapiens fibroblast growth factor receptor 3 (achondroplasia, thanatophoric dwarfism) (FGFR3), transcript variant 2, mRNA. |
| NM_015833.2 | ADARB1 | 21 | Homo sapiens adenosine deaminase, RNA-specific, B1 (RED1 homolog rat) (ADARB1), transcript variant 2, mRNA. |
| NM_003407.1 | ZFP36 | 19 | Homo sapiens zinc finger protein 36, C3H type, homolog (mouse) (ZFP36), mRNA. |
| NM_032918.1 | RERG | 12 | Homo sapiens RAS-like, estrogen-regulated, growth inhibitor (RERG), mRNA. |
| NM_006216.2 | SERPINE2 | 2 | Homo sapiens serpin peptidase inhibitor, clade E (nexin, plasminogen activator inhibitor type 1), member 2 (SERPINE2), mRNA. |
| NM_022748.9 | TNS3 | 7 | Homo sapiens tensin 3 (TNS3), mRNA. |
| XR_017149.2 | LOC392437 | X | PREDICTED: Homo sapiens misc_RNA (LOC392437), miscRNA. |
| NM_005025.2 | SERPINI1 | 3 | Homo sapiens serpin peptidase inhibitor, clade I (neuroserpin), member 1 (SERPINI1), mRNA. |
| NM_017445.1 | HIST1H2BK | 6 | Homo sapiens histone cluster 1, H2bk (HIST1H2BK), mRNA. |
| NM_001080535.1 | LINCR | 2 | Homo sapiens likely ortholog of mouse lung-inducible Neutralized-related C3HC4 RING domain protein (LINCR), mRNA. |
| XM_930284.1 | LOC441763 | 16 | PREDICTED: Homo sapiens hypothetical LOC441763 (LOC441763), mRNA. |
| NM_005461.3 | MAFB | 20 | Homo sapiens v-maf musculoaponeurotic fibrosarcoma oncogene homolog B (avian) (MAFB), mRNA. |
| NM_001040152.1 | PEG10 | 7 | Homo sapiens paternally expressed 10 (PEG10), transcript variant 1, mRNA. XM_940378 |
| NM_015444.1 | TMEM158 | 3 | Homo sapiens transmembrane protein 158 (TMEM158), mRNA. |
| XM_928128.1 | LOC643431 | 5 | PREDICTED: Homo sapiens similar to Keratin, type II cytoskeletal 8 (Cytokeratin-8) (CK-8) (Keraton-8) (K8) (LOC643431), mRNA. |
| NM_002600.3 | PDE4B | 1 | Homo sapiens phosphodiesterase 4B, cAMP-specific (phosphodiesterase E4 dunce homolog, Drosophila) (PDE4B), transcript variant a, mRNA. |
| NM_207380.1 | C15ORF52 | 15 | Homo sapiens chromosome 15 open reading frame 52 (C15orf52), mRNA. |
| NM_001011668.1 | CHCHD7 | 8 | Homo sapiens coiled-coil-helix-coiled-coil-helix domain containing 7 (CHCHD7), transcript variant 6, mRNA. |
| NM_006340.1 | BAIAP2 | 17 | Homo sapiens BAI1-associated protein 2 (BAIAP2), transcript variant 3, mRNA. |
| NM_004091.2 | E2F2 | 1 | Homo sapiens E2F transcription factor 2 (E2F2), mRNA. |
| NM_021158.3 | TRIB3 | 20 | Homo sapiens tribbles homolog 3 (Drosophila) (TRIB3), mRNA. |
| XM_001724542.1 | LOC100133565 |  | PREDICTED: Homo sapiens similar to hCG23738 (LOC100133565), mRNA. |
| NM_024626.1 | VTCN1 | 1 | Homo sapiens V-set domain containing T cell activation inhibitor 1 (VTCN1), mRNA. |
| NM_013376.1 | SERTAD1 | 19 | Homo sapiens SERTA domain containing 1 (SERTAD1), mRNA. |
| NM_002105.2 | H2AFX | 11 | Homo sapiens H2A histone family, member X (H2AFX), mRNA. |
| NM_153840.2 | GPR110 | 6 | Homo sapiens G protein-coupled receptor 110 (GPR110), transcript variant 1, mRNA. |
| XR_017231.2 | KRT8P9 | 15 | PREDICTED: Homo sapiens misc_RNA (KRT8P9), miscRNA. |
| NM_006079.3 | CITED2 | 6 | Homo sapiens Cbp/p300-interacting transactivator, with Glu/Asp-rich carboxy-terminal domain, 2 (CITED2), transcript variant 1, mRNA. |
| NM_003810.2 | TNFSF10 | 3 | Homo sapiens tumor necrosis factor (ligand) superfamily, member 10 (TNFSF10), mRNA. |
| NM_018645.3 | HES6 | 2 | Homo sapiens hairy and enhancer of split 6 (Drosophila) (HES6), mRNA. |
| NM_001007023.1 | DIO2 | 14 | Homo sapiens deiodinase, iodothyronine, type II (DIO2), transcript variant 3, mRNA. |
| XM_934731.1 | BOLA2 | 16 | Homo sapiens bolA homolog 2 (E. coli) (BOLA2), mRNA. |
| NM_000224.2 | KRT18 | 12 | Homo sapiens keratin 18 (KRT18), transcript variant 1, mRNA. |
| NM_012408.3 | ZMYND8 | 20 | Homo sapiens zinc finger, MYND-type containing 8 (ZMYND8), transcript variant 1, mRNA. |
| NM_013370.2 | OKL38 | 16 | Homo sapiens pregnancy-induced growth inhibitor (OKL38), transcript variant 1, mRNA. |
| NM_004029.2 | IRF7 | 11 | Homo sapiens interferon regulatory factor 7 (IRF7), transcript variant b, mRNA. |
| NM_021229.3 | NTN4 | 12 | Homo sapiens netrin 4 (NTN4), mRNA. |
| XM_499343.2 | PEG10 |  | PREDICTED: Homo sapiens paternally expressed 10 (PEG10), mRNA. |
| NM_005104.2 | BRD2 | 6 | Homo sapiens bromodomain containing 2 (BRD2), mRNA. |
| NM_017572.2 | MKNK2 | 19 | Homo sapiens MAP kinase interacting serine/threonine kinase 2 (MKNK2), transcript variant 1, mRNA. |
| NM_153747.1 | PIGC | 1 | Homo sapiens phosphatidylinositol glycan anchor biosynthesis, class C (PIGC), transcript variant 1, mRNA. |
| NM_002037.3 | FYN | 6 | Homo sapiens FYN oncogene related to SRC, FGR, YES (FYN), transcript variant 1, mRNA. |
| NM_199054.1 | MKNK2 | 19 | Homo sapiens MAP kinase interacting serine/threonine kinase 2 (MKNK2), transcript variant 2, mRNA. |
| NM_015878.4 | AZIN1 | 8 | Homo sapiens antizyme inhibitor 1 (AZIN1), transcript variant 1, mRNA. |
| NM_024578.1 | OCEL1 | 19 | Homo sapiens occludin/ELL domain containing 1 (OCEL1), mRNA. |
| NM_001956.2 | EDN2 | 1 | Homo sapiens endothelin 2 (EDN2), mRNA. |
| NM_001904.2 | CTNNB1 | 3 | Homo sapiens catenin (cadherin-associated protein), beta 1, 88kDa (CTNNB1), transcript variant 2, mRNA. |
| NM_198857.1 | SLC6A10P | 16 | Homo sapiens solute carrier family 6 (neurotransmitter transporter, creatine), member 10 (pseudogene) (SLC6A10P) on chromosome 16. |
| NM_005378.4 | MYCN | 2 | Homo sapiens v-myc myelocytomatosis viral related oncogene, neuroblastoma derived (avian) (MYCN), mRNA. |
| NM_004753.4 | DHRS3 | 1 | Homo sapiens dehydrogenase/reductase (SDR family) member 3 (DHRS3), mRNA. |
| NM_024889.3 | C10ORF81 | 10 | Homo sapiens chromosome 10 open reading frame 81 (C10orf81), mRNA. |
| NM_152649.1 | MLKL | 16 | Homo sapiens mixed lineage kinase domain-like (MLKL), mRNA. |
| NM_080593.1 | HIST1H2BK | 6 | Homo sapiens histone cluster 1, H2bk (HIST1H2BK), mRNA. |
| NM_003516.2 | HIST2H2AA3 | 1 | Homo sapiens histone cluster 2, H2aa3 (HIST2H2AA3), mRNA. |
| NM_058187.3 | C21ORF63 | 21 | Homo sapiens chromosome 21 open reading frame 63 (C21orf63), mRNA. |
| NM_006088.5 | TUBB2C | 9 | Homo sapiens tubulin, beta 2C (TUBB2C), mRNA. |
| NM_001901.1 | CTGF | 6 | Homo sapiens connective tissue growth factor (CTGF), mRNA. |
| NM_006026.2 | H1FX | 3 | Homo sapiens H1 histone family, member X (H1FX), mRNA. |
| NM_003543.3 | HIST1H4H | 6 | Homo sapiens histone cluster 1, H4h (HIST1H4H), mRNA. |
| NM_001013642.1 | TRNP1 | 1 | Homo sapiens TMF1-regulated nuclear protein 1 (TRNP1), mRNA. |
| NM_003273.1 | TM7SF2 | 11 | Homo sapiens transmembrane 7 superfamily member 2 (TM7SF2), mRNA. |
| NM_005841.1 | SPRY1 | 4 | Homo sapiens sprouty homolog 1, antagonist of FGF signaling (Drosophila) (SPRY1), transcript variant 2, mRNA. |
| NM_020899.2 | ZBTB4 | 17 | Homo sapiens zinc finger and BTB domain containing 4 (ZBTB4), mRNA. |
| NM_001024912.1 | CEACAM1 | 19 | Homo sapiens carcinoembryonic antigen-related cell adhesion molecule 1 (biliary glycoprotein) (CEACAM1), transcript variant 2, mRNA. |
| TUBB | TUBB2A | 6 | Homo sapiens tubulin, beta 2A (TUBB2A), mRNA. |
| NM_018670.1 | MESP1 | 15 | Homo sapiens mesoderm posterior 1 homolog (mouse) (MESP1), mRNA. |
| NM_001630.1 | ANXA8 | 10 | Homo sapiens annexin A8 (ANXA8), mRNA. |
| NM_016084.3 | RASD1 | 17 | Homo sapiens RAS, dexamethasone-induced 1 (RASD1), mRNA. |
| NM_130840.1 | ATP6V0A4 | 7 | Homo sapiens ATPase, H+ transporting, lysosomal V0 subunit a4 (ATP6V0A4), transcript variant 1, mRNA. |
| NM_173495.1 | PTCHD1 | X | Homo sapiens patched domain containing 1 (PTCHD1), mRNA. |
| NM_017709.2 | FAM46C | 1 | Homo sapiens family with sequence similarity 46, member C (FAM46C), mRNA. |
| NM_001114981.1 | TP63 | 3 | Homo sapiens tumor protein p63 (TP63), transcript variant 5, mRNA. |
| NM_130840.2 | ATP6V0A4 | 7 | Homo sapiens ATPase, H+ transporting, lysosomal V0 subunit a4 (ATP6V0A4), transcript variant 2, mRNA. |
| NM_005345.4 | HSPA1A | 6 | Homo sapiens heat shock 70kDa protein 1A (HSPA1A), mRNA. |
| NM_000856.2 | GUCY1A3 | 4 | Homo sapiens guanylate cyclase 1, soluble, alpha 3 (GUCY1A3), mRNA. |
| NM_000937.2 | POLR2A | 17 | Homo sapiens polymerase (RNA) II (DNA directed) polypeptide A, 220kDa (POLR2A), mRNA. |
| NM_017734.4 | PALMD | 1 | Homo sapiens palmdelphin (PALMD), mRNA. |
| NM_003548.2 | HIST2H4A | 1 | Homo sapiens histone cluster 2, H4a (HIST2H4A), mRNA. |
| XM_943555.1 | TMEM191B | 22 | PREDICTED: Homo sapiens transmembrane protein 191B, transcript variant 2 (TMEM191B), mRNA. |
| NM_005542.3 | INSIG1 | 7 | Homo sapiens insulin induced gene 1 (INSIG1), transcript variant 2, mRNA. |
| NM_153840.2 | GPR110 | 6 | Homo sapiens G protein-coupled receptor 110 (GPR110), transcript variant 1, mRNA. |
| NM_022726.2 | ELOVL4 | 6 | Homo sapiens elongation of very long chain fatty acids (FEN1/Elo2, SUR4/Elo3, yeast)-like 4 (ELOVL4), mRNA. |
| NM_014817.2 | TRIL | 7 | Homo sapiens TLR4 interactor with leucine rich repeats (TRIL), mRNA. |
| NM_014604.2 | TAX1BP3 | 17 | Homo sapiens Tax1 (human T-cell leukemia virus type I) binding protein 3 (TAX1BP3), mRNA. |
| NM_004508.2 | IDI1 | 10 | Homo sapiens isopentenyl-diphosphate delta isomerase 1 (IDI1), mRNA. |
| NM_183048.1 | ZMYND8 | 20 | Homo sapiens zinc finger, MYND-type containing 8 (ZMYND8), transcript variant 3, mRNA. |
| NM_000856.2 | GUCY1A3 | 4 | Homo sapiens guanylate cyclase 1, soluble, alpha 3 (GUCY1A3), mRNA. |
| NM_001080450.1 | BEND3 | 6 | Homo sapiens BEN domain containing 3 (BEND3), mRNA. |
| NM_001901.2 | CTGF | 6 | Homo sapiens connective tissue growth factor (CTGF), mRNA. |
| NM_000146.3 | FTL | 19 | Homo sapiens ferritin, light polypeptide (FTL), mRNA. |
| NM_020744.2 | MTA3 | 2 | Homo sapiens metastasis associated 1 family, member 3 (MTA3), mRNA. |
| NM_080792.1 | SIRPA | 20 | Homo sapiens signal-regulatory protein alpha (SIRPA), transcript variant 2, mRNA. |
| NM_178134.2 | CYP4Z1 | 1 | Homo sapiens cytochrome P450, family 4, subfamily Z, polypeptide 1 (CYP4Z1), mRNA. |
| NM_005738.2 | ARL4A | 7 | Homo sapiens ADP-ribosylation factor-like 4A (ARL4A), transcript variant 1, mRNA. |
| NM_003517.2 | HIST2H2AC | 1 | Homo sapiens histone cluster 2, H2ac (HIST2H2AC), mRNA. |
| NM_005476.3 | GNE | 9 | Homo sapiens glucosamine (UDP-N-acetyl)-2-epimerase/N-acetylmannosamine kinase (GNE), mRNA. |
| NM_014969.3 | WDR47 | 1 | Homo sapiens WD repeat domain 47 (WDR47), mRNA. |
| NM_006045.1 | ATP9A | 20 | Homo sapiens ATPase, class II, type 9A (ATP9A), mRNA. |
| NM_016323.1 | HERC5 | 4 | Homo sapiens hect domain and RLD 5 (HERC5), mRNA. |
| NM_177401.4 | MIDN | 19 | Homo sapiens midnolin (MIDN), mRNA. |
| NM_002185.2 | IL7R | 5 | Homo sapiens interleukin 7 receptor (IL7R), mRNA. |
| NM_052962.2 | IL22RA2 | 6 | Homo sapiens interleukin 22 receptor, alpha 2 (IL22RA2), transcript variant 1, mRNA. |
| NM_002250.2 | KCNN4 | 19 | Homo sapiens potassium intermediate/small conductance calcium-activated channel, subfamily N, member 4 (KCNN4), mRNA. |
| NM_001033049.1 | ADARB1 | 21 | Homo sapiens adenosine deaminase, RNA-specific, B1 (RED1 homolog rat) (ADARB1), transcript variant 4, mRNA. |
| XM_035299.7 | ZSWIM6 | 5 | PREDICTED: Homo sapiens zinc finger, SWIM-type containing 6 (ZSWIM6), mRNA. |
| NM_015286.4 | SYNM | 15 | Homo sapiens synemin, intermediate filament protein (SYNM), transcript variant B, mRNA. |
| NM_001945.1 | HBEGF | 5 | Homo sapiens heparin-binding EGF-like growth factor (HBEGF), mRNA. |
| NM_000839.2 | GRM2 | 3 | Homo sapiens glutamate receptor, metabotropic 2 (GRM2), mRNA. |
| NM_020127.1 | TUFT1 | 1 | Homo sapiens tuftelin 1 (TUFT1), mRNA. |
| XR_037197.1 | LOC392437 |  | PREDICTED: Homo sapiens misc_RNA (LOC392437), miscRNA. |
| NM_030965.1 | ST6GALNAC5 | 1 | Homo sapiens ST6 (alpha-N-acetyl-neuraminyl-2,3-beta-galactosyl-1, 3)-N-acetylgalactosaminide alpha-2,6-sialyltransferase 5 (ST6GALNAC5), mRNA. |
| XM_938141.1 | LOC647954 |  | PREDICTED: Homo sapiens misc_RNA (LOC647954), miscRNA. |
| NM_001321.1 | CSRP2 | 12 | Homo sapiens cysteine and glycine-rich protein 2 (CSRP2), mRNA. |
| XR_018203.2 | LOC644563 |  | PREDICTED: Homo sapiens misc_RNA (LOC644563), miscRNA. |
| NM_002862.3 | PYGB | 20 | Homo sapiens phosphorylase, glycogen; brain (PYGB), mRNA. |
| NM_007235.3 | LOC650215 |  | PREDICTED: Homo sapiens similar to Exportin-T (tRNA exportin) (Exportin(tRNA)) (LOC650215), mRNA. |
| NM_207343.1 | RNF214 | 11 | Homo sapiens ring finger protein 214 (RNF214), transcript variant 1, mRNA. |
| XR_015626.2 | KRT17P3 | 17 | PREDICTED: Homo sapiens misc_RNA (KRT17P3), miscRNA. |
| NM_001165.3 | BIRC3 | 11 | Homo sapiens baculoviral IAP repeat-containing 3 (BIRC3), transcript variant 1, mRNA. |
| NM_001001684.1 | FLJ45831 | 17 | Homo sapiens FLJ45831 protein (FLJ45831), mRNA. |
| NM_004364.2 | CEBPA | 19 | Homo sapiens CCAAT/enhancer binding protein (C/EBP), alpha (CEBPA), mRNA. |
| NM_152339.2 | SPATA2L | 16 | Homo sapiens spermatogenesis associated 2-like (SPATA2L), mRNA. |
| NM_000146.3 | FTL | 19 | Homo sapiens ferritin, light polypeptide (FTL), mRNA. |
| NM_178134.2 | CYP4Z1 | 1 | Homo sapiens cytochrome P450, family 4, subfamily Z, polypeptide 1 (CYP4Z1), mRNA. |
| NM_024901.3 | DENND2D | 1 | Homo sapiens DENN/MADD domain containing 2D (DENND2D), mRNA. |
| NM_020040.3 | TUBB4Q | 4 | Homo sapiens tubulin, beta polypeptide 4, member Q (TUBB4Q), mRNA. |
| NM_018649.1 | H2AFY2 | 10 | Homo sapiens H2A histone family, member Y2 (H2AFY2), mRNA. |
| NM_199050.1 | C2CD2 | 21 | Homo sapiens C2 calcium-dependent domain containing 2 (C2CD2), transcript variant 1, mRNA. |
| Hs.537002 | HS.537002 | X | Homo sapiens cDNA clone IMAGE:4811759 |
| NM_033405.2 | PRIC285 | 20 | Homo sapiens peroxisomal proliferator-activated receptor A interacting complex 285 (PRIC285), transcript variant 2, mRNA. |
| NM_001514.3 | GTF2B | 1 | Homo sapiens general transcription factor IIB (GTF2B), mRNA. |
| NM_024915.1 | GRHL2 | 8 | Homo sapiens grainyhead-like 2 (Drosophila) (GRHL2), mRNA. |
| NM_001080501.1 | MGC3196 |  | Homo sapiens hypothetical protein MGC3196 (MGC3196), mRNA. |
| NM_032174.4 | TOMM40L | 1 | Homo sapiens translocase of outer mitochondrial membrane 40 homolog (yeast)-like (TOMM40L), nuclear gene encoding mitochondrial protein, mRNA. |
| NM_004089.3 | TSC22D3 | X | Homo sapiens TSC22 domain family, member 3 (TSC22D3), transcript variant 2, mRNA. |
| NM_017750.2 | RETSAT | 2 | Homo sapiens retinol saturase (all-trans-retinol 13,14-reductase) (RETSAT), mRNA. |
| NM_002167.2 | ID3 | 1 | Homo sapiens inhibitor of DNA binding 3, dominant negative helix-loop-helix protein (ID3), mRNA. |
| NM_005841.1 | SPRY1 | 4 | Homo sapiens sprouty homolog 1, antagonist of FGF signaling (Drosophila) (SPRY1), transcript variant 1, mRNA. |
| NM_033267.2 | IRX2 | 5 | Homo sapiens iroquois homeobox 2 (IRX2), mRNA. |
| NM_182908.3 | DHRS2 | 14 | Homo sapiens dehydrogenase/reductase (SDR family) member 2 (DHRS2), transcript variant 1, mRNA. |
| NM_022818.3 | MAP1LC3B | 16 | Homo sapiens microtubule-associated protein 1 light chain 3 beta (MAP1LC3B), mRNA. |
| NM_004454.1 | ETV5 | 3 | Homo sapiens ets variant gene 5 (ets-related molecule) (ETV5), mRNA. |
| NM_181726.1 | ANKRD37 | 4 | Homo sapiens ankyrin repeat domain 37 (ANKRD37), mRNA. |
| NM_015074.2 | KIF1B | 1 | Homo sapiens kinesin family member 1B (KIF1B), transcript variant 1, mRNA. |
| NM_003722.3 | TP73L | 3 | Homo sapiens tumor protein p73-like (TP73L), mRNA. |
| NM_013262.3 | MYLIP | 6 | Homo sapiens myosin regulatory light chain interacting protein (MYLIP), mRNA. |
| XM_928356.1 | TMEM191B | 22 | PREDICTED: Homo sapiens transmembrane protein 191B, transcript variant 2 (TMEM191B), mRNA. |
| NM_198505.2 | ATP13A5 | 3 | Homo sapiens ATPase type 13A5 (ATP13A5), mRNA. |
| NM_001013398.1 | IGFBP3 | 7 | Homo sapiens insulin-like growth factor binding protein 3 (IGFBP3), transcript variant 1, mRNA. |
| NM_002037.3 | FYN | 6 | Homo sapiens FYN oncogene related to SRC, FGR, YES (FYN), transcript variant 2, mRNA. |
| NM_001017424.1 | KCNK2 | 1 | Homo sapiens potassium channel, subfamily K, member 2 (KCNK2), transcript variant 3, mRNA. |
| NM_017666.2 | ZNF280C | X | Homo sapiens zinc finger protein 280C (ZNF280C), mRNA. |
| NM_012135.1 | FAM50B | 6 | Homo sapiens family with sequence similarity 50, member B (FAM50B), mRNA. |
| NM_004526.2 | MCM2 | 3 | Homo sapiens minichromosome maintenance complex component 2 (MCM2), mRNA. |
| NM_138447.1 | ZNF689 | 16 | Homo sapiens zinc finger protein 689 (ZNF689), mRNA. |
| NM_017658.2 | KLHL28 | 14 | Homo sapiens kelch-like 28 (Drosophila) (KLHL28), mRNA. |
| NM_033446.1 | FAM125B | 9 | Homo sapiens family with sequence similarity 125, member B (FAM125B), transcript variant 1, mRNA. |
| NM_022117.1 | TSPYL2 | X | Homo sapiens TSPY-like 2 (TSPYL2), mRNA. |
| NM_015349.1 | KIAA0240 | 6 | Homo sapiens KIAA0240 (KIAA0240), mRNA. |
| NM_031917.2 | ANGPTL6 | 19 | Homo sapiens angiopoietin-like 6 (ANGPTL6), mRNA. |
| NM_001280.1 | CIRBP | 19 | Homo sapiens cold inducible RNA binding protein (CIRBP), mRNA. |
| NM_032354.2 | TMEM107 | 17 | Homo sapiens transmembrane protein 107 (TMEM107), transcript variant 1, mRNA. |
| XM_938988.1 | LOC402221 |  | PREDICTED: Homo sapiens similar to actin alpha 1 skeletal muscle protein (LOC402221), mRNA. |
| NM_001031615.1 | ALDH3B2 | 11 | Homo sapiens aldehyde dehydrogenase 3 family, member B2 (ALDH3B2), transcript variant 2, mRNA. |
| NM_004040.2 | RHOB | 2 | Homo sapiens ras homolog gene family, member B (RHOB), mRNA. |
| NM_207362.1 | MGC42367 | 2 | Homo sapiens similar to 2010300C02Rik protein (MGC42367), mRNA. |
| NM_021732.1 | AVPI1 | 10 | Homo sapiens arginine vasopressin-induced 1 (AVPI1), mRNA. |
| NM_003108.3 | SOX11 | 2 | Homo sapiens SRY (sex determining region Y)-box 11 (SOX11), mRNA. |
| NM_003873.3 | NRP1 | 10 | Homo sapiens neuropilin 1 (NRP1), transcript variant 1, mRNA. |
| NM_006022.2 | TSC22D1 | 13 | Homo sapiens TSC22 domain family, member 1 (TSC22D1), transcript variant 2, mRNA. |
| NM_001001850.1 | STX19 | 3 | Homo sapiens syntaxin 19 (STX19), mRNA. |
| NM_199328.1 | CLDN8 | 21 | Homo sapiens claudin 8 (CLDN8), mRNA. |
| NM_001077242.1 | DEPDC7 | 11 | Homo sapiens DEP domain containing 7 (DEPDC7), transcript variant 1, mRNA. |
| NM_000274.1 | OAT | 10 | Homo sapiens ornithine aminotransferase (gyrate atrophy) (OAT), nuclear gene encoding mitochondrial protein, mRNA. |
| NM_001031694.1 | SCMH1 | 1 | Homo sapiens sex comb on midleg homolog 1 (Drosophila) (SCMH1), transcript variant 1, mRNA. |
| NM_014740.2 | EIF4A3 | 17 | Homo sapiens eukaryotic translation initiation factor 4A, isoform 3 (EIF4A3), mRNA. |
| NM_032181.1 | TMEM166 | 2 | Homo sapiens transmembrane protein 166 (TMEM166), mRNA. |
| NM_178450.2 | Mar-03 |  | PREDICTED: Homo sapiens membrane-associated ring finger (C3HC4) 3 (MARCH3), mRNA. |
| NM_015508.2 | TIPARP | 3 | Homo sapiens TCDD-inducible poly(ADP-ribose) polymerase (TIPARP), mRNA. |
| NM_001031706.1 | PLEKHB2 | 2 | Homo sapiens pleckstrin homology domain containing, family B (evectins) member 2 (PLEKHB2), transcript variant 1, mRNA. |
| NM_006875.2 | PIM2 | X | Homo sapiens pim-2 oncogene (PIM2), mRNA. |
| NM_018181.4 | ZNF532 | 18 | Homo sapiens zinc finger protein 532 (ZNF532), mRNA. |
| NM_012383.3 | OSTF1 | 9 | Homo sapiens osteoclast stimulating factor 1 (OSTF1), mRNA. |
| NM_001008219.1 | AMY1C | 1 | Homo sapiens amylase, alpha 1C (salivary) (AMY1C), mRNA. |
| NR_002560.1 | SNORD31 | 11 | Homo sapiens small nucleolar RNA, C/D box 31 (SNORD31), small nucleolar RNA. |
| NM_052943.2 | FAM46B | 1 | Homo sapiens family with sequence similarity 46, member B (FAM46B), mRNA. |
| NM_001924.2 | GADD45A | 1 | Homo sapiens growth arrest and DNA-damage-inducible, alpha (GADD45A), mRNA. |
| NM_020149.2 | MEIS2 | 15 | Homo sapiens Meis homeobox 2 (MEIS2), transcript variant g, mRNA. |
| NM_016297.2 | PCYOX1 | 2 | Homo sapiens prenylcysteine oxidase 1 (PCYOX1), mRNA. |
| Hs.505676 | HS.505676 | 12 | Homo sapiens cDNA FLJ33772 fis, clone BRSSN2000175 |
| NM_001024024.1 | GCH1 | 14 | Homo sapiens GTP cyclohydrolase 1 (GCH1), transcript variant 4, mRNA. |
| NM_005120.1 | MED12 | X | Homo sapiens mediator of RNA polymerase II transcription, subunit 12 homolog (yeast) (MED12), mRNA. |
| NM_198336.1 | INSIG1 | 7 | Homo sapiens insulin induced gene 1 (INSIG1), transcript variant 2, mRNA. |
| XM_938009.1 | Mar-03 |  | PREDICTED: Homo sapiens membrane-associated ring finger (C3HC4) 3 (MARCH3), mRNA. |
| XM_933997.1 | LOC284023 |  | PREDICTED: Homo sapiens hypothetical protein LOC284023, transcript variant 3 (LOC284023), mRNA. |
| NM_005252.2 | FOS | 14 | Homo sapiens v-fos FBJ murine osteosarcoma viral oncogene homolog (FOS), mRNA. |
| NM_003047.2 | SLC9A1 | 1 | Homo sapiens solute carrier family 9 (sodium/hydrogen exchanger), member 1 (SLC9A1), mRNA. |
| NM_001885.1 | CRYAB | 11 | Homo sapiens crystallin, alpha B (CRYAB), mRNA. |
| NM_153742.3 | CTH | 1 | Homo sapiens cystathionase (cystathionine gamma-lyase) (CTH), transcript variant 2, mRNA. |
| NM_003302.1 | TRIP6 | 7 | Homo sapiens thyroid hormone receptor interactor 6 (TRIP6), mRNA. |
| NM_199329.1 | SLC43A3 | 11 | Homo sapiens solute carrier family 43, member 3 (SLC43A3), mRNA. |
| NM_018478.2 | DBNDD2 | 20 | Homo sapiens dysbindin (dystrobrevin binding protein 1) domain containing 2 (DBNDD2), transcript variant 3, mRNA. |
| NM_023015.3 | INTS3 | 1 | Homo sapiens integrator complex subunit 3 (INTS3), mRNA. |
| NM_005324.3 | H3F3B | 17 | Homo sapiens H3 histone, family 3B (H3.3B) (H3F3B), mRNA. |
| XM_001713607.1 | LOC100129668 | 6 | PREDICTED: Homo sapiens hypothetical protein LOC100129668 (LOC100129668), mRNA. |
| NM_031845.1 | MAP2 | 2 | Homo sapiens microtubule-associated protein 2 (MAP2), transcript variant 2, mRNA. |
| NM_001012642.1 | GRAMD2 | 15 | Homo sapiens GRAM domain containing 2 (GRAMD2), mRNA. |
| NM_152892.1 | LRWD1 | 7 | Homo sapiens leucine-rich repeats and WD repeat domain containing 1 (LRWD1), mRNA. |
| NM_016185.2 | HN1 | 17 | Homo sapiens hematological and neurological expressed 1 (HN1), transcript variant 1, mRNA. |
| NM_001017928.2 | CCDC58 | 3 | Homo sapiens coiled-coil domain containing 58 (CCDC58), mRNA. |
| NM_003714.2 | STC2 | 5 | Homo sapiens stanniocalcin 2 (STC2), mRNA. |
| NM_001034077.4 | HIST2H4B | 1 | Homo sapiens histone cluster 2, H4b (HIST2H4B), mRNA. |
| NM_005125.1 | CCS | 11 | Homo sapiens copper chaperone for superoxide dismutase (CCS), mRNA. |
| NM_033025.4 | SYDE1 | 19 | Homo sapiens synapse defective 1, Rho GTPase, homolog 1 (C. elegans) (SYDE1), mRNA. |
| NM_138425.2 | C12ORF57 | 12 | Homo sapiens chromosome 12 open reading frame 57 (C12orf57), mRNA. |
| NM_012247.3 | SEPHS1 | 10 | Homo sapiens selenophosphate synthetase 1 (SEPHS1), mRNA. |
| NM_001024070.1 | GCH1 | 14 | Homo sapiens GTP cyclohydrolase 1 (GCH1), transcript variant 3, mRNA. |
| NR_002561.1 | SNORD30 | 11 | Homo sapiens small nucleolar RNA, C/D box 30 (SNORD30), small nucleolar RNA. |
| NM_018335.2 | C14ORF131 | 14 | Homo sapiens chromosome 14 open reading frame 131 (C14orf131), mRNA. |
| NM_000104.2 | CYP1B1 | 2 | Homo sapiens cytochrome P450, family 1, subfamily B, polypeptide 1 (CYP1B1), mRNA. |
| NM_003900.3 | SQSTM1 | 5 | Homo sapiens sequestosome 1 (SQSTM1), mRNA. |
| NM_145739.1 | OSBPL6 | 2 | Homo sapiens oxysterol binding protein-like 6 (OSBPL6), transcript variant 1, mRNA. |
| NM_002304.1 | LFNG | 7 | Homo sapiens LFNG O-fucosylpeptide 3-beta-N-acetylglucosaminyltransferase (LFNG), transcript variant 1, mRNA. |
| NM_015288.4 | PHF15 | 5 | Homo sapiens PHD finger protein 15 (PHF15), mRNA. |
| Hs.555260 | HS.555260 | X | 17000532215901 GRN_ES Homo sapiens cDNA 5, mRNA sequence |
| NM_016830.2 | VAMP1 | 12 | Homo sapiens vesicle-associated membrane protein 1 (synaptobrevin 1) (VAMP1), transcript variant 2, mRNA. |
| NM_001013699.1 | LOC440093 | 12 | Homo sapiens histone H3-like (LOC440093), mRNA. |
| NM_176797.1 | P2RY6 | 11 | Homo sapiens pyrimidinergic receptor P2Y, G-protein coupled, 6 (P2RY6), transcript variant 3, mRNA. |
| NM_002888.2 | RARRES1 | 3 | Homo sapiens retinoic acid receptor responder (tazarotene induced) 1 (RARRES1), transcript variant 2, mRNA. |
| NM_003518.3 | HIST1H2BG | 6 | Homo sapiens histone cluster 1, H2bg (HIST1H2BG), mRNA. |
| NM_175859.1 | CTPS2 | X | Homo sapiens CTP synthase II (CTPS2), transcript variant 2, mRNA. |
| NM_153367.1 | ZCCHC24 | 10 | Homo sapiens zinc finger, CCHC domain containing 24 (ZCCHC24), mRNA. |
| NM_003536.2 | HIST1H3H | 6 | Homo sapiens histone cluster 1, H3h (HIST1H3H), mRNA. |
| NM_006888.2 | CALM1 | 14 | Homo sapiens calmodulin 1 (phosphorylase kinase, delta) (CALM1), mRNA. |
| NM_003915.2 | RBM12 | 20 | Homo sapiens RNA binding motif protein 12 (RBM12), transcript variant 1, mRNA. |
| NM_012238.3 | SIRT1 | 10 | Homo sapiens sirtuin (silent mating type information regulation 2 homolog) 1 (S. cerevisiae) (SIRT1), mRNA. |
| NM_016059.3 | PPIL1 | 6 | Homo sapiens peptidylprolyl isomerase (cyclophilin)-like 1 (PPIL1), mRNA. |
| NM_152227.1 | SNX5 | 20 | Homo sapiens sorting nexin 5 (SNX5), transcript variant 1, mRNA. |
| NM_021095.1 | SLC5A6 | 2 | Homo sapiens solute carrier family 5 (sodium-dependent vitamin transporter), member 6 (SLC5A6), mRNA. |
| NM_020244.2 | CHPT1 | 12 | Homo sapiens choline phosphotransferase 1 (CHPT1), mRNA. |
| NM_002374.3 | MAP2 | 2 | Homo sapiens microtubule-associated protein 2 (MAP2), transcript variant 1, mRNA. |
| NM_001032221.1 | STXBP1 | 9 | Homo sapiens syntaxin binding protein 1 (STXBP1), transcript variant 2, mRNA. |
| XM_001720815.1 | LOC100133609 |  | PREDICTED: Homo sapiens similar to membrane-associated ring finger (C3HC4) 3 (LOC100133609), mRNA. |
| NM_002193.1 | INHBB | 2 | Homo sapiens inhibin, beta B (activin AB beta polypeptide) (INHBB), mRNA. |
| NM_014044.4 | UNC50 | 2 | Homo sapiens unc-50 homolog (C. elegans) (UNC50), mRNA. |
| NM_138408.2 | GTF3C6 | 6 | Homo sapiens general transcription factor IIIC, polypeptide 6, alpha 35kDa (GTF3C6), mRNA. |
| XM_001718809.1 | LOC100134794 | 7 | PREDICTED: Homo sapiens similar to keratin 8 (LOC100134794), mRNA. |
| NM_012247.3 | SEPHS1 | 10 | Homo sapiens selenophosphate synthetase 1 (SEPHS1), mRNA. |
| NM_000434.2 | NEU1 | 6 | Homo sapiens sialidase 1 (lysosomal sialidase) (NEU1), mRNA. |
| NM_002423.3 | MMP7 | 11 | Homo sapiens matrix metallopeptidase 7 (matrilysin, uterine) (MMP7), mRNA. |
| NM_015675.1 | GADD45B | 19 | Homo sapiens growth arrest and DNA-damage-inducible, beta (GADD45B), mRNA. |
| NM_182757.2 | RNF144B | 6 | Homo sapiens ring finger protein 144B (RNF144B), mRNA. |
| NM_021809.4 | TGIF2 | 20 | Homo sapiens TGFB-induced factor homeobox 2 (TGIF2), mRNA. |
| NM_005700.2 | DPP3 | 11 | Homo sapiens dipeptidyl-peptidase 3 (DPP3), transcript variant 1, mRNA. |
| NM_032993.2 | GAR1 | 4 | Homo sapiens GAR1 ribonucleoprotein homolog (yeast) (GAR1), transcript variant 2, mRNA. |
| NM_032730.3 | RTN4IP1 | 6 | Homo sapiens reticulon 4 interacting protein 1 (RTN4IP1), nuclear gene encoding mitochondrial protein, mRNA. |
| NM_012432.2 | SETDB1 | 1 | Homo sapiens SET domain, bifurcated 1 (SETDB1), mRNA. |
| NM_004502.2 | HOXB7 | 17 | Homo sapiens homeobox B7 (HOXB7), mRNA. |
| NM_001823.3 | CKB | 14 | Homo sapiens creatine kinase, brain (CKB), mRNA. |
| NM_001011670.1 | CHCHD7 | 8 | Homo sapiens coiled-coil-helix-coiled-coil-helix domain containing 7 (CHCHD7), transcript variant 5, mRNA. |
| NM_005410.2 | SEPP1 | 5 | Homo sapiens selenoprotein P, plasma, 1 (SEPP1), transcript variant 1, mRNA. |
| NM_000247.1 | MICA | 6 | Homo sapiens MHC class I polypeptide-related sequence A (MICA), mRNA. |
| NM_005318.2 | H1F0 | 22 | Homo sapiens H1 histone family, member 0 (H1F0), mRNA. |
| NM_001031628.1 | SMAGP | 12 | Homo sapiens small cell adhesion glycoprotein (SMAGP), transcript variant 2, mRNA. |
| NM_001511.1 | CXCL1 | 4 | Homo sapiens chemokine (C-X-C motif) ligand 1 (melanoma growth stimulating activity, alpha) (CXCL1), mRNA. |
| NM_024098.1 | CCDC86 | 11 | Homo sapiens coiled-coil domain containing 86 (CCDC86), mRNA. |
| NM_001017425.2 | KCNK2 | 1 | Homo sapiens potassium channel, subfamily K, member 2 (KCNK2), transcript variant 3, mRNA. |
| NM_199328.1 | CLDN8 | 21 | Homo sapiens claudin 8 (CLDN8), mRNA. |
| XR_000996.1 | CYP4Z2P |  | PREDICTED: Homo sapiens cytochrome P450 4Z2 pseudogene (CYP4Z2P), misc RNA. |
| NM_000617.1 | SLC11A2 | 12 | Homo sapiens solute carrier family 11 (proton-coupled divalent metal ion transporters), member 2 (SLC11A2), mRNA. |
| XM_938649.1 | PIPSL | 10 | Homo sapiens PIP5K1A and PSMD4-like (PIPSL), non-coding RNA. |
| NM_016267.2 | VGLL1 | X | Homo sapiens vestigial like 1 (Drosophila) (VGLL1), mRNA. |
| NM_000917.2 | P4HA1 | 10 | Homo sapiens procollagen-proline, 2-oxoglutarate 4-dioxygenase (proline 4-hydroxylase), alpha polypeptide I (P4HA1), transcript variant 1, mRNA. |
| NM_002153.1 | HSD17B2 | 16 | Homo sapiens hydroxysteroid (17-beta) dehydrogenase 2 (HSD17B2), mRNA. |
| NM_001347.2 | DGKQ | 4 | Homo sapiens diacylglycerol kinase, theta 110kDa (DGKQ), mRNA. |
| XM_930977.1 | ARHGAP23 | 17 | PREDICTED: Homo sapiens Rho GTPase activating protein 23, transcript variant 1 (ARHGAP23), mRNA. |
| NM_014856.2 | DENND4B | 1 | Homo sapiens DENN/MADD domain containing 4B (DENND4B), mRNA. |
| NM_005178.2 | BCL3 | 19 | Homo sapiens B-cell CLL/lymphoma 3 (BCL3), mRNA. |
| NM_000421.2 | KRT10 | 17 | Homo sapiens keratin 10 (epidermolytic hyperkeratosis; keratosis palmaris et plantaris) (KRT10), mRNA. |
| XM_926584.1 | LOC653110 | 10 | PREDICTED: Homo sapiens similar to annexin A8, transcript variant 1 (LOC653110), mRNA. |
| NM_001151.2 | SLC25A4 | 4 | Homo sapiens solute carrier family 25 (mitochondrial carrier; adenine nucleotide translocator), member 4 (SLC25A4), nuclear gene encoding mitochondrial protein, mRNA. |
| NM_014750.3 | DLGAP5 | 14 | Homo sapiens discs, large (Drosophila) homolog-associated protein 5 (DLGAP5), mRNA. |
| NM_006825.2 | CKAP4 | 12 | Homo sapiens cytoskeleton-associated protein 4 (CKAP4), mRNA. |
| NM_000270.1 | NP | 14 | Homo sapiens nucleoside phosphorylase (NP), mRNA. |
| NM_022575.2 | VPS16 | 20 | Homo sapiens vacuolar protein sorting 16 homolog (S. cerevisiae) (VPS16), transcript variant 1, mRNA. |
| NM_021960.3 | MCL1 | 1 | Homo sapiens myeloid cell leukemia sequence 1 (BCL2-related) (MCL1), transcript variant 1, mRNA. |
| Hs.201441 | HS.201441 | 13 | Homo sapiens cDNA FLJ11076 fis, clone PLACE1005077 |
| NM_014002.2 | IKBKE | 1 | Homo sapiens inhibitor of kappa light polypeptide gene enhancer in B-cells, kinase epsilon (IKBKE), mRNA. |
| NM_020808.1 | SIPA1L2 | 1 | Homo sapiens signal-induced proliferation-associated 1 like 2 (SIPA1L2), mRNA. |
| NM_138340.3 | ABHD3 | 18 | Homo sapiens abhydrolase domain containing 3 (ABHD3), mRNA. |
| NM_000676.2 | ADORA2B | 17 | Homo sapiens adenosine A2b receptor (ADORA2B), mRNA. |
| NM_004665.2 | VNN2 | 6 | Homo sapiens vanin 2 (VNN2), transcript variant 1, mRNA. |
| NM_018846.2 | KLHL7 | 7 | Homo sapiens kelch-like 7 (Drosophila) (KLHL7), transcript variant 2, mRNA. |
| NM_020760.1 | HECW2 | 2 | Homo sapiens HECT, C2 and WW domain containing E3 ubiquitin protein ligase 2 (HECW2), mRNA. |
| XM_926231.1 | P704P | 14 | PREDICTED: Homo sapiens prostate-specific P704P (P704P), mRNA. |
| NM_001135032.1 | FAM176A | 2 | Homo sapiens family with sequence similarity 176, member A (FAM176A), transcript variant 1, mRNA. |
| NM_139178.1 | ALKBH3 | 11 | Homo sapiens alkB, alkylation repair homolog 3 (E. coli) (ALKBH3), mRNA. |
| NM_018660.2 | ZNF395 | 8 | Homo sapiens zinc finger protein 395 (ZNF395), mRNA. |
| NM_001007067.1 | SDCBP | 8 | Homo sapiens syndecan binding protein (syntenin) (SDCBP), transcript variant 2, mRNA. |
| NM_007047.3 | BTN3A2 | 6 | Homo sapiens butyrophilin, subfamily 3, member A2 (BTN3A2), mRNA. |
| XM_935791.1 | C7ORF70 | 7 | Homo sapiens chromosome 7 open reading frame 70 (C7orf70), mRNA. |
| NM_002085.2 | GPX4 | 19 | Homo sapiens glutathione peroxidase 4 (phospholipid hydroperoxidase) (GPX4), transcript variant 2, mRNA. |
| NM_005629.1 | SLC6A8 | X | Homo sapiens solute carrier family 6 (neurotransmitter transporter, creatine), member 8 (SLC6A8), mRNA. |
| NM_005080.2 | XBP1 | 22 | Homo sapiens X-box binding protein 1 (XBP1), transcript variant 1, mRNA. |
| NM_198434.1 | AURKA | 20 | Homo sapiens aurora kinase A (AURKA), transcript variant 3, mRNA. |
| NM_004566.2 | PFKFB3 | 10 | Homo sapiens 6-phosphofructo-2-kinase/fructose-2,6-biphosphatase 3 (PFKFB3), mRNA. |
| NM_012188.3 | FOXI1 | 5 | Homo sapiens forkhead box I1 (FOXI1), transcript variant 1, mRNA. |
| NM_005418.3 | ST5 | 11 | Homo sapiens suppression of tumorigenicity 5 (ST5), transcript variant 3, mRNA. |
| NM_014220.2 | TM4SF1 | 3 | Homo sapiens transmembrane 4 L six family member 1 (TM4SF1), mRNA. |
| NM_022740.2 | HIPK2 | 7 | Homo sapiens homeodomain interacting protein kinase 2 (HIPK2), mRNA. |
| NM_006745.3 | SC4MOL | 4 | Homo sapiens sterol-C4-methyl oxidase-like (SC4MOL), transcript variant 1, mRNA. |
| NM_005873.1 | RGS19 | 20 | Homo sapiens regulator of G-protein signaling 19 (RGS19), transcript variant 1, mRNA. |
| NM_016286.2 | DCXR | 17 | Homo sapiens dicarbonyl/L-xylulose reductase (DCXR), mRNA. |
| NR_002312.1 | RPPH1 | 14 | Homo sapiens ribonuclease P RNA component H1 (RPPH1), RNase P RNA. |
| NM_020228.2 | PRDM10 | 11 | Homo sapiens PR domain containing 10 (PRDM10), transcript variant 1, mRNA. |
| NM_031448.2 | C19ORF12 | 19 | Homo sapiens chromosome 19 open reading frame 12 (C19orf12), transcript variant 1, mRNA. |
| NM_203504.1 | G3BP2 | 4 | Homo sapiens GTPase activating protein (SH3 domain) binding protein 2 (G3BP2), transcript variant 3, mRNA. |
| NM_032822.1 | FAM136A | 2 | Homo sapiens family with sequence similarity 136, member A (FAM136A), mRNA. |
| NM_002251.3 | KCNS1 | 20 | Homo sapiens potassium voltage-gated channel, delayed-rectifier, subfamily S, member 1 (KCNS1), mRNA. |
| NM_001007026.1 | ATN1 | 12 | Homo sapiens atrophin 1 (ATN1), transcript variant 1, mRNA. |
| NM_153374.1 | LYSMD2 | 15 | Homo sapiens LysM, putative peptidoglycan-binding, domain containing 2 (LYSMD2), mRNA. |
| NM_012475.4 | USP21 | 1 | Homo sapiens ubiquitin specific peptidase 21 (USP21), transcript variant 1, mRNA. |
| NM_014315.2 | KLHDC2 | 14 | Homo sapiens kelch domain containing 2 (KLHDC2), mRNA. |
| NM_004192.1 | ASMTL | XY | Homo sapiens acetylserotonin O-methyltransferase-like (ASMTL), mRNA. |
| NM_001020820.1 | MYADM | 19 | Homo sapiens myeloid-associated differentiation marker (MYADM), transcript variant 4, mRNA. |
| NM_033016.1 | PDGFB | 22 | Homo sapiens platelet-derived growth factor beta polypeptide (simian sarcoma viral (v-sis) oncogene homolog) (PDGFB), transcript variant 1, mRNA. |
| NM_001621.3 | AHR | 7 | Homo sapiens aryl hydrocarbon receptor (AHR), mRNA. |
| NM_001024912.1 | CEACAM1 | 19 | Homo sapiens carcinoembryonic antigen-related cell adhesion molecule 1 (biliary glycoprotein) (CEACAM1), transcript variant 2, mRNA. |
| NM_022840.2 | METTL4 | 18 | Homo sapiens methyltransferase like 4 (METTL4), mRNA. |
| NM_022893.2 | BCL11A | 2 | Homo sapiens B-cell CLL/lymphoma 11A (zinc finger protein) (BCL11A), transcript variant 1, mRNA. |
| XM_935818.1 | HEATR2 | 7 | Homo sapiens HEAT repeat containing 2 (HEATR2), mRNA. XM_935824 XM_935825 |
| NM_004192.1 | ASMTL | Y | Homo sapiens acetylserotonin O-methyltransferase-like (ASMTL), mRNA. |
| NM_005534.2 | IFNGR2 | 21 | Homo sapiens interferon gamma receptor 2 (interferon gamma transducer 1) (IFNGR2), mRNA. |
| NM_080677.1 | DYNLL2 | 17 | Homo sapiens dynein, light chain, LC8-type 2 (DYNLL2), mRNA. |
| NM_014138.3 | FAM156A | X | Homo sapiens family with sequence similarity 156, member A (FAM156A), mRNA. |
| NM_015104.1 | ATG2A | 11 | Homo sapiens ATG2 autophagy related 2 homolog A (S. cerevisiae) (ATG2A), mRNA. |
| NM_017693.2 | BIVM | 13 | Homo sapiens basic, immunoglobulin-like variable motif containing (BIVM), mRNA. |
| NM_004776.2 | B4GALT5 | 20 | Homo sapiens UDP-Gal:betaGlcNAc beta 1,4- galactosyltransferase, polypeptide 5 (B4GALT5), mRNA. |
| NM_001964.2 | EGR1 | 5 | Homo sapiens early growth response 1 (EGR1), mRNA. |
| NM_000199.2 | SGSH | 17 | Homo sapiens N-sulfoglucosamine sulfohydrolase (sulfamidase) (SGSH), mRNA. |
| NM_138771.2 | CCDC126 | 7 | Homo sapiens coiled-coil domain containing 126 (CCDC126), mRNA. |
| NM_000416.1 | IFNGR1 | 6 | Homo sapiens interferon gamma receptor 1 (IFNGR1), mRNA. |
| XM_496202.2 | LOC650517 |  | PREDICTED: Homo sapiens hypothetical LOC650517 (LOC650517), mRNA. |
| NM_032554.2 | GPR81 | 12 | Homo sapiens G protein-coupled receptor 81 (GPR81), mRNA. |
| NM_002982.3 | CCL2 | 17 | Homo sapiens chemokine (C-C motif) ligand 2 (CCL2), mRNA. |
| NM_139160.1 | DEPDC7 | 11 | Homo sapiens DEP domain containing 7 (DEPDC7), transcript variant 2, mRNA. |
| NM_014235.2 | UBL4A | X | Homo sapiens ubiquitin-like 4A (UBL4A), mRNA. |
| NM_017759.4 | INO80D | 2 | Homo sapiens INO80 complex subunit D (INO80D), mRNA. |
| NM_021960.3 | MCL1 | 1 | Homo sapiens myeloid cell leukemia sequence 1 (BCL2-related) (MCL1), transcript variant 1, mRNA. |
| NM_015516.3 | TSKU | 11 | Homo sapiens tsukushin (TSKU), mRNA. |
| NM_020919.2 | ALS2 | 2 | Homo sapiens amyotrophic lateral sclerosis 2 (juvenile) (ALS2), mRNA. |
| NM_007156.3 | ZXDA | X | Homo sapiens zinc finger, X-linked, duplicated A (ZXDA), mRNA. |
| NM_032046.1 | TMPRSS13 | 11 | Homo sapiens transmembrane protease, serine 13 (TMPRSS13), mRNA. |
| XM_939368.1 | LOC654103 |  | PREDICTED: Homo sapiens similar to solute carrier family 25, member 37 (LOC654103), mRNA. |
| NM_013401.2 | RAB3IL1 | 11 | Homo sapiens RAB3A interacting protein (rabin3)-like 1 (RAB3IL1), mRNA. |
| NM_004428.2 | EFNA1 | 1 | Homo sapiens ephrin-A1 (EFNA1), transcript variant 1, mRNA. |
| NM_001007595.1 | NLF2 |  | PREDICTED: Homo sapiens nuclear localized factor 2 (NLF2), mRNA. |
| NM_018043.5 | ANO1 | 11 | Homo sapiens anoctamin 1, calcium activated chloride channel (ANO1), transcript variant 1, mRNA. |
| NM_003976.2 | ARTN | 1 | Homo sapiens artemin (ARTN), transcript variant 2, mRNA. |
| NM_001921.2 | DCTD | 4 | Homo sapiens dCMP deaminase (DCTD), transcript variant 2, mRNA. |
| NM_032262.1 | DKFZP434N035 | 22 | Homo sapiens hypothetical protein DKFZp434N035 (DKFZp434N035), mRNA. |
| NM_015554.1 | GLCE | 15 | Homo sapiens glucuronic acid epimerase (GLCE), mRNA. |
| NM_138440.1 | VASN | 16 | Homo sapiens vasorin (VASN), mRNA. |
| NM_004949.2 | DSC2 | 18 | Homo sapiens desmocollin 2 (DSC2), transcript variant Dsc2b, mRNA. |
| NM_024430.2 | PSTPIP2 | 18 | Homo sapiens proline-serine-threonine phosphatase interacting protein 2 (PSTPIP2), mRNA. |
| NM_025165.2 | ELL3 | 15 | Homo sapiens elongation factor RNA polymerase II-like 3 (ELL3), mRNA. |
| NM_003034.2 | ST8SIA1 | 12 | Homo sapiens ST8 alpha-N-acetyl-neuraminide alpha-2,8-sialyltransferase 1 (ST8SIA1), mRNA. |
| NM_000393.2 | COL5A2 | 2 | Homo sapiens collagen, type V, alpha 2 (COL5A2), mRNA. |
| NM_033086.1 | FGD3 | 9 | Homo sapiens FYVE, RhoGEF and PH domain containing 3 (FGD3), transcript variant 2, mRNA. |
| NM_001004431.1 | METRNL |  | PREDICTED: Homo sapiens meteorin, glial cell differentiation regulator-like (METRNL), mRNA. |
| XM_938904.1 | BANP | 16 | Homo sapiens BTG3 associated nuclear protein (BANP), transcript variant 2, mRNA. |
| NM_006454.2 | MXD4 | 4 | Homo sapiens MAX dimerization protein 4 (MXD4), mRNA. |
| NM_030769.1 | NPL | 1 | Homo sapiens N-acetylneuraminate pyruvate lyase (dihydrodipicolinate synthase) (NPL), mRNA. |
| NM_001032280.1 | TFAP2A | 6 | Homo sapiens transcription factor AP-2 alpha (activating enhancer binding protein 2 alpha) (TFAP2A), transcript variant 3, mRNA. |
| NM_003981.2 | PRC1 | 15 | Homo sapiens protein regulator of cytokinesis 1 (PRC1), transcript variant 2, mRNA. |
| NM_012289.3 | KEAP1 | 19 | Homo sapiens kelch-like ECH-associated protein 1 (KEAP1), transcript variant 2, mRNA. |
| NM_001013398.1 | IGFBP3 | 7 | Homo sapiens insulin-like growth factor binding protein 3 (IGFBP3), transcript variant 2, mRNA. |
| NM_019049.1 | C1ORF218 | 1 | Homo sapiens chromosome 1 open reading frame 218 (C1orf218), mRNA. |
| NM_001042618.1 | PARP2 | 14 | Homo sapiens poly (ADP-ribose) polymerase 2 (PARP2), transcript variant 2, mRNA. |
| NM_031924.3 | RSPH3 | 6 | Homo sapiens radial spoke 3 homolog (Chlamydomonas) (RSPH3), mRNA. |
| XM_927769.1 | LOC653506 | 17 | PREDICTED: Homo sapiens similar to meteorin, glial cell differentiation regulator-like (LOC653506), mRNA. |
| NM_014474.2 | SMPDL3B | 1 | Homo sapiens sphingomyelin phosphodiesterase, acid-like 3B (SMPDL3B), transcript variant 1, mRNA. |
| NM_172070.2 | ZNF650 | 2 | Homo sapiens zinc finger protein 650 (ZNF650), mRNA. |
| NM_003635.2 | NDST2 | 10 | Homo sapiens N-deacetylase/N-sulfotransferase (heparan glucosaminyl) 2 (NDST2), mRNA. |
| NM_022572.2 | PNKD | 2 | Homo sapiens paroxysmal nonkinesiogenic dyskinesia (PNKD), transcript variant 2, mRNA. |
| NM_024517.1 | PHF2 | 9 | Homo sapiens PHD finger protein 2 (PHF2), mRNA. |
| XM_937579.1 | LOC648526 |  | PREDICTED: Homo sapiens similar to epiplakin 1 (LOC648526), mRNA. |
| NM_001001390.1 | CD44 | 11 | Homo sapiens CD44 molecule (Indian blood group) (CD44), transcript variant 4, mRNA. |
| NM_153690.4 | FAM43A | 3 | Homo sapiens family with sequence similarity 43, member A (FAM43A), mRNA. |
| NM_020159.1 | SMARCAD1 | 4 | Homo sapiens SWI/SNF-related, matrix-associated actin-dependent regulator of chromatin, subfamily a, containing DEAD/H box 1 (SMARCAD1), mRNA. |
| NM_021018.2 | HIST1H3F | 6 | Homo sapiens histone cluster 1, H3f (HIST1H3F), mRNA. |
| NM_002586.3 | PBX2 | 6 | Homo sapiens pre-B-cell leukemia homeobox 2 (PBX2), mRNA. |
| NM_138371.1 | FAM113B | 12 | Homo sapiens family with sequence similarity 113, member B (FAM113B), mRNA. |
| NM_018983.3 | GAR1 | 4 | Homo sapiens GAR1 ribonucleoprotein homolog (yeast) (GAR1), transcript variant 1, mRNA. |
| NM_001039847.1 | GPX4 | 19 | Homo sapiens glutathione peroxidase 4 (phospholipid hydroperoxidase) (GPX4), transcript variant 2, mRNA. |
| NM_000086.2 | CLN3 | 16 | Homo sapiens ceroid-lipofuscinosis, neuronal 3 (CLN3), transcript variant 2, mRNA. |
| NM_144665.2 | SESN3 | 11 | Homo sapiens sestrin 3 (SESN3), mRNA. |
| NM_004038.3 | AMY1A | 1 | Homo sapiens amylase, alpha 1A (salivary) (AMY1A), transcript variant 1, mRNA. |
| NM_001852.3 | COL9A2 | 1 | Homo sapiens collagen, type IX, alpha 2 (COL9A2), mRNA. |
| NM_000252.1 | MTM1 | X | Homo sapiens myotubularin 1 (MTM1), mRNA. |
| NM_207644.1 | C22ORF36 | 22 | Homo sapiens chromosome 22 open reading frame 36 (C22orf36), mRNA. |
| NM_000086.1 | CLN3 | 16 | Homo sapiens ceroid-lipofuscinosis, neuronal 3 (CLN3), transcript variant 1, mRNA. |
| NM_012112.4 | TPX2 | 20 | Homo sapiens TPX2, microtubule-associated, homolog (Xenopus laevis) (TPX2), mRNA. |
| NM_144590.1 | ANKRD22 | 10 | Homo sapiens ankyrin repeat domain 22 (ANKRD22), mRNA. |
| NM_001031696.1 | PLD3 | 19 | Homo sapiens phospholipase D family, member 3 (PLD3), transcript variant 1, mRNA. |
| NM_016472.3 | C14ORF129 | 14 | Homo sapiens chromosome 14 open reading frame 129 (C14orf129), mRNA. |
| NM_001924.2 | GADD45A | 1 | Homo sapiens growth arrest and DNA-damage-inducible, alpha (GADD45A), mRNA. |
| NM_002957.3 | RXRA | 9 | Homo sapiens retinoid X receptor, alpha (RXRA), mRNA. |
| NM_000274.1 | OAT | 10 | Homo sapiens ornithine aminotransferase (gyrate atrophy) (OAT), nuclear gene encoding mitochondrial protein, mRNA. |
| NM_173362.2 | RFESD | 5 | Homo sapiens Rieske (Fe-S) domain containing (RFESD), mRNA. |
| NM_005606.5 | LGMN | 14 | Homo sapiens legumain (LGMN), transcript variant 2, mRNA. |
| NM_013313.3 | YPEL1 | 22 | Homo sapiens yippee-like 1 (Drosophila) (YPEL1), mRNA. |
| NM_024665.3 | TBL1XR1 | 3 | Homo sapiens transducin (beta)-like 1X-linked receptor 1 (TBL1XR1), mRNA. |
| NM_144636.1 | CHCHD4 | 3 | Homo sapiens coiled-coil-helix-coiled-coil-helix domain containing 4 (CHCHD4), nuclear gene encoding mitochondrial protein, transcript variant 2, mRNA. |
| NM_001034.1 | RRM2 | 2 | Homo sapiens ribonucleotide reductase M2 polypeptide (RRM2), mRNA. |
| NM_198219.1 | ING1 | 13 | Homo sapiens inhibitor of growth family, member 1 (ING1), transcript variant 1, mRNA. |
| XM_926857.1 | LOC648399 |  | PREDICTED: Homo sapiens similar to Ornithine aminotransferase, mitochondrial precursor (Ornithine--oxo-acid aminotransferase) (LOC648399), mRNA. |
| NM_001800.3 | CDKN2D | 19 | Homo sapiens cyclin-dependent kinase inhibitor 2D (p19, inhibits CDK4) (CDKN2D), transcript variant 2, mRNA. |
| NM_003937.2 | KYNU | 2 | Homo sapiens kynureninase (L-kynurenine hydrolase) (KYNU), transcript variant 2, mRNA. |
| NM_207343.2 | RNF214 | 11 | Homo sapiens ring finger protein 214 (RNF214), transcript variant 1, mRNA. |
| NM_018321.3 | BRIX1 | 5 | Homo sapiens BRX1, biogenesis of ribosomes, homolog (S. cerevisiae) (BRIX1), mRNA. |
| NM_017958.1 | PLEKHB2 | 2 | Homo sapiens pleckstrin homology domain containing, family B (evectins) member 2 (PLEKHB2), transcript variant 2, mRNA. |
| NM_003311.3 | PHLDA2 | 11 | Homo sapiens pleckstrin homology-like domain, family A, member 2 (PHLDA2), mRNA. |
| NM_152240.1 | ZMAT3 | 3 | Homo sapiens zinc finger, matrin type 3 (ZMAT3), transcript variant 2, mRNA. |
| XM_942395.1 | CYP4Z1 | 1 | Homo sapiens cytochrome P450, family 4, subfamily Z, polypeptide 1 (CYP4Z1), mRNA. |
| NM_147173.1 | NUDT2 | 9 | Homo sapiens nudix (nucleoside diphosphate linked moiety X)-type motif 2 (NUDT2), transcript variant 3, mRNA. |
| NM_032472.3 | PPIL3 | 2 | Homo sapiens peptidylprolyl isomerase (cyclophilin)-like 3 (PPIL3), transcript variant PPIL3b, mRNA. |
| NM_153747.1 | PIGC | 1 | Homo sapiens phosphatidylinositol glycan anchor biosynthesis, class C (PIGC), transcript variant 1, mRNA. |
| NM_178006.1 | STARD13 | 13 | Homo sapiens START domain containing 13 (STARD13), transcript variant alpha, mRNA. |
| NM_017421.2 | COQ3 | 6 | Homo sapiens coenzyme Q3 homolog, methyltransferase (S. cerevisiae) (COQ3), mRNA. |
| NM_006340.1 | BAIAP2 | 17 | Homo sapiens BAI1-associated protein 2 (BAIAP2), transcript variant 3, mRNA. |
| NM_014840.2 | NUAK1 | 12 | Homo sapiens NUAK family, SNF1-like kinase, 1 (NUAK1), mRNA. |
| NM_005928.1 | MFGE8 | 15 | Homo sapiens milk fat globule-EGF factor 8 protein (MFGE8), mRNA. |
| NM_003679.2 | KMO | 1 | Homo sapiens kynurenine 3-monooxygenase (kynurenine 3-hydroxylase) (KMO), mRNA. |
| NM_181803.1 | UBE2C | 20 | Homo sapiens ubiquitin-conjugating enzyme E2C (UBE2C), transcript variant 6, mRNA. |
| NM_018993.2 | RIN2 | 20 | Homo sapiens Ras and Rab interactor 2 (RIN2), mRNA. |
| NM_002137.3 | HNRNPA2B1 | 7 | Homo sapiens heterogeneous nuclear ribonucleoprotein A2/B1 (HNRNPA2B1), transcript variant A2, mRNA. |
| NM_014840.2 | NUAK1 | 12 | Homo sapiens NUAK family, SNF1-like kinase, 1 (NUAK1), mRNA. |
| NM_030809.1 | CSRNP2 | 12 | Homo sapiens cysteine-serine-rich nuclear protein 2 (CSRNP2), mRNA. |
| NM_001017370.1 | NHLRC3 | 13 | Homo sapiens NHL repeat containing 3 (NHLRC3), transcript variant 2, mRNA. |
| NM_173083.2 | LIN9 | 1 | Homo sapiens lin-9 homolog (C. elegans) (LIN9), mRNA. |
| NM_002600.2 | PDE4B | 1 | Homo sapiens phosphodiesterase 4B, cAMP-specific (phosphodiesterase E4 dunce homolog, Drosophila) (PDE4B), transcript variant a, mRNA. |
| NM_022356.2 | LEPRE1 | 1 | Homo sapiens leucine proline-enriched proteoglycan (leprecan) 1 (LEPRE1), mRNA. |
| NM_005484.2 | PARP2 | 14 | Homo sapiens poly (ADP-ribose) polymerase family, member 2 (PARP2), mRNA. |
| NM_004729.3 | ZBED1 | Y | Homo sapiens zinc finger, BED-type containing 1 (ZBED1), mRNA. |
| NM_032154.3 | PCGF6 | 10 | Homo sapiens polycomb group ring finger 6 (PCGF6), transcript variant 2, mRNA. |
| NM_004237.2 | TRIP13 | 5 | Homo sapiens thyroid hormone receptor interactor 13 (TRIP13), mRNA. |
| NM_001042535.1 | AGAP3 | 7 | Homo sapiens ArfGAP with GTPase domain, ankyrin repeat and PH domain 3 (AGAP3), transcript variant 2, mRNA. |
| NM_001007067.1 | SDCBP | 8 | Homo sapiens syndecan binding protein (syntenin) (SDCBP), transcript variant 2, mRNA. |
| NM_003344.2 | UBE2H | 7 | Homo sapiens ubiquitin-conjugating enzyme E2H (UBC8 homolog, yeast) (UBE2H), transcript variant 1, mRNA. |
| NR_002565.1 | SNORD25 | 11 | Homo sapiens small nucleolar RNA, C/D box 25 (SNORD25), small nucleolar RNA. |
| NM_002342.1 | LTBR | 12 | Homo sapiens lymphotoxin beta receptor (TNFR superfamily, member 3) (LTBR), mRNA. |
| NM_006795.2 | EHD1 | 11 | Homo sapiens EH-domain containing 1 (EHD1), mRNA. |
| NM_022893.2 | BCL11A | 2 | Homo sapiens B-cell CLL/lymphoma 11A (zinc finger protein) (BCL11A), transcript variant 1, mRNA. |
| NM_000745.2 | CHRNA5 | 15 | Homo sapiens cholinergic receptor, nicotinic, alpha 5 (CHRNA5), mRNA. |
| NM_198434.1 | AURKA | 20 | Homo sapiens aurora kinase A (AURKA), transcript variant 5, mRNA. |
| NM_015689.2 | DENND2A | 7 | Homo sapiens DENN/MADD domain containing 2A (DENND2A), mRNA. |
| NM_001079539.1 | XBP1 | 22 | Homo sapiens X-box binding protein 1 (XBP1), transcript variant 2, mRNA. |
| NM_001354.4 | AKR1C2 | 10 | Homo sapiens aldo-keto reductase family 1, member C2 (dihydrodiol dehydrogenase 2; bile acid binding protein; 3-alpha hydroxysteroid dehydrogenase, type III) (AKR1C2), transcript variant 1, mRNA. XM_943424 XM_943425 XM_943427 |
| NM_020940.2 | KIAA1600 | 10 | Homo sapiens KIAA1600 (KIAA1600), mRNA. |
| NM_018043.4 | TMEM16A | 11 | Homo sapiens transmembrane protein 16A (TMEM16A), mRNA. |
| NM_001337.3 | CX3CR1 | 3 | Homo sapiens chemokine (C-X3-C motif) receptor 1 (CX3CR1), mRNA. |
| NM_005463.2 | HNRPDL | 4 | Homo sapiens heterogeneous nuclear ribonucleoprotein D-like (HNRPDL), transcript variant 3, transcribed RNA. |
| NM_018137.1 | PRMT6 | 1 | Homo sapiens protein arginine methyltransferase 6 (PRMT6), mRNA. |
| NM_005134.1 | PPP4R1 | 18 | Homo sapiens protein phosphatase 4, regulatory subunit 1 (PPP4R1), transcript variant 2, mRNA. |
| NM_001040437.1 | C6ORF48 | 6 | Homo sapiens chromosome 6 open reading frame 48 (C6orf48), transcript variant 1, mRNA. |
| NM_005148.2 | UNC119 | 17 | Homo sapiens unc-119 homolog (C. elegans) (UNC119), transcript variant 2, mRNA. |
| NM_022750.2 | PARP12 | 7 | Homo sapiens poly (ADP-ribose) polymerase family, member 12 (PARP12), mRNA. |
| NM_017542.3 | POGK | 1 | Homo sapiens pogo transposable element with KRAB domain (POGK), mRNA. |
| XM_937100.1 | LOC728285 | 17 | PREDICTED: Homo sapiens similar to keratin associated protein 2-4 (LOC728285), mRNA. |
| NM_001112.2 | ADARB1 | 21 | Homo sapiens adenosine deaminase, RNA-specific, B1 (RED1 homolog rat) (ADARB1), transcript variant 1, mRNA. |
| NM_024036.3 | LRFN4 | 11 | Homo sapiens leucine rich repeat and fibronectin type III domain containing 4 (LRFN4), mRNA. |
| NM_178450.2 | Mar-03 | 5 | Homo sapiens membrane-associated ring finger (C3HC4) 3 (MARCH3), mRNA. |
| NM_004172.3 | SLC1A3 | 5 | Homo sapiens solute carrier family 1 (glial high affinity glutamate transporter), member 3 (SLC1A3), mRNA. |
| Hs.534279 | FOXO3 | 6 | Homo sapiens forkhead box O3 (FOXO3), transcript variant 2, mRNA. |
| NM_014804.1 | KIAA0753 | 17 | Homo sapiens KIAA0753 (KIAA0753), mRNA. |
| NM_030769.1 | NPL | 1 | Homo sapiens N-acetylneuraminate pyruvate lyase (dihydrodipicolinate synthase) (NPL), mRNA. |
| NM_001860.1 | SLC31A2 | 9 | Homo sapiens solute carrier family 31 (copper transporters), member 2 (SLC31A2), mRNA. |
| NM_017940.2 | NBPF1 |  | Homo sapiens neuroblastoma breakpoint family, member 1 (NBPF1), mRNA. XM_934962 XM_934964 XM_934966 XM_934967 XM_934969 XM_934972 XM_934973 XM_934974 XM_934975 XM_934976 XM_934978 |
| NM_004000.2 | CHI3L2 | 1 | Homo sapiens chitinase 3-like 2 (CHI3L2), transcript variant 1, mRNA. |
| NM_001135032.1 | FAM176A | 2 | Homo sapiens family with sequence similarity 176, member A (FAM176A), transcript variant 1, mRNA. |
| NM_013282.2 | UHRF1 | 19 | Homo sapiens ubiquitin-like with PHD and ring finger domains 1 (UHRF1), transcript variant 1, mRNA. |
| NM_139212.2 | HOPX | 4 | Homo sapiens HOP homeobox (HOPX), transcript variant 3, mRNA. |
| NM_001083538.1 | POTEE | 2 | Homo sapiens POTE ankyrin domain family, member E (POTEE), mRNA. |
| NM_006984.3 | CLDN10 | 13 | Homo sapiens claudin 10 (CLDN10), transcript variant 2, mRNA. |
| NM_173614.2 | NOMO1 | 16 | Homo sapiens NODAL modulator 1 (NOMO1), mRNA. |
| NM_015135.1 | NUP205 | 7 | Homo sapiens nucleoporin 205kDa (NUP205), mRNA. |
| NM_012456.1 | TIMM10 | 11 | Homo sapiens translocase of inner mitochondrial membrane 10 homolog (yeast) (TIMM10), nuclear gene encoding mitochondrial protein, mRNA. |
| NM_000509.4 | FGG | 4 | Homo sapiens fibrinogen gamma chain (FGG), transcript variant gamma-A, mRNA. |
| NM_003373.3 | VCL | 10 | Homo sapiens vinculin (VCL), transcript variant 2, mRNA. |
| NM_020379.1 | MAN1C1 | 1 | Homo sapiens mannosidase, alpha, class 1C, member 1 (MAN1C1), mRNA. |
| NM_031415.1 | GSDMC | 8 | Homo sapiens gasdermin C (GSDMC), mRNA. |
| NM_018845.1 | RAG1AP1 | 1 | Homo sapiens recombination activating gene 1 activating protein 1 (RAG1AP1), mRNA. |
| NM_175859.1 | CTPS2 | X | Homo sapiens CTP synthase II (CTPS2), transcript variant 2, mRNA. |
| NR_015366.2 | LOC388796 |  | Homo sapiens hypothetical LOC388796 (LOC388796), non-coding RNA. |
| NM_001099684.1 | FAM156B | X | Homo sapiens family with sequence similarity 156, member B (FAM156B), mRNA. |
| NM_153048.1 | FYN | 6 | Homo sapiens FYN oncogene related to SRC, FGR, YES (FYN), transcript variant 3, mRNA. |
| NM_016021.2 | UBE2J1 | 6 | Homo sapiens ubiquitin-conjugating enzyme E2, J1 (UBC6 homolog, yeast) (UBE2J1), mRNA. |
| NM_147184.1 | TP53I3 | 2 | Homo sapiens tumor protein p53 inducible protein 3 (TP53I3), transcript variant 2, mRNA. |
| NM_016499.2 | TMEM216 | 11 | Homo sapiens transmembrane protein 216 (TMEM216), mRNA. |
| NM_006134.5 | TMEM50B | 21 | Homo sapiens transmembrane protein 50B (TMEM50B), mRNA. |
| NM_144564.4 | SLC39A3 | 19 | Homo sapiens solute carrier family 39 (zinc transporter), member 3 (SLC39A3), transcript variant 1, mRNA. |
| NM_032727.2 | INA | 10 | Homo sapiens internexin neuronal intermediate filament protein, alpha (INA), mRNA. |
| NM_015033.2 | FNBP1 | 9 | Homo sapiens formin binding protein 1 (FNBP1), mRNA. |
| NM_001008704.1 | C6ORF1 | 6 | Homo sapiens chromosome 6 open reading frame 1 (C6orf1), transcript variant 3, mRNA. |
| NM_018718.1 | TSGA14 | 7 | Homo sapiens testis specific, 14 (TSGA14), mRNA. |
| NM_017953.2 | ZNHIT6 | 1 | Homo sapiens zinc finger, HIT type 6 (ZNHIT6), mRNA. |
| NM_000903.2 | NQO1 | 16 | Homo sapiens NAD(P)H dehydrogenase, quinone 1 (NQO1), transcript variant 1, mRNA. |
| NM_003879.3 | CFLAR | 2 | Homo sapiens CASP8 and FADD-like apoptosis regulator (CFLAR), mRNA. |
| NM_018433.3 | JMJD1A | 2 | Homo sapiens jumonji domain containing 1A (JMJD1A), mRNA. |
| NM_014177.1 | C18ORF55 | 18 | Homo sapiens chromosome 18 open reading frame 55 (C18orf55), mRNA. |
| NM_003130.2 | SRI | 7 | Homo sapiens sorcin (SRI), transcript variant 2, mRNA. |
| NM_001951.2 | E2F5 | 8 | Homo sapiens E2F transcription factor 5, p130-binding (E2F5), transcript variant 1, mRNA. |
| NM_080605.2 | B3GALT6 | 1 | Homo sapiens UDP-Gal:betaGal beta 1,3-galactosyltransferase polypeptide 6 (B3GALT6), mRNA. |
| NM_004380.1 | CREBBP | 16 | Homo sapiens CREB binding protein (CREBBP), transcript variant 2, mRNA. |
| NM_153338.1 | GGT6 | 17 | Homo sapiens gamma-glutamyltransferase 6 homolog (rat) (GGT6), mRNA. |
| NM_017865.2 | ZNF692 | 1 | Homo sapiens zinc finger protein 692 (ZNF692), transcript variant 2, mRNA. |
| NM_005611.2 | RBL2 | 16 | Homo sapiens retinoblastoma-like 2 (p130) (RBL2), mRNA. |
| NM_015169.3 | RRS1 | 8 | Homo sapiens RRS1 ribosome biogenesis regulator homolog (S. cerevisiae) (RRS1), mRNA. |
| NM_004273.2 | CHST3 | 10 | Homo sapiens carbohydrate (chondroitin 6) sulfotransferase 3 (CHST3), mRNA. |
| NM_005764.3 | PDZK1IP1 | 1 | Homo sapiens PDZK1 interacting protein 1 (PDZK1IP1), mRNA. |
| NM_032199.1 | ARID5B | 10 | Homo sapiens AT rich interactive domain 5B (MRF1-like) (ARID5B), mRNA. |
| NM_000693.1 | ALDH1A3 | 15 | Homo sapiens aldehyde dehydrogenase 1 family, member A3 (ALDH1A3), mRNA. |
| Hs.12876 | HS.12876 | 6 | 602659965F1 NCI_CGAP_Skn3 Homo sapiens cDNA clone IMAGE:4802969 5, mRNA sequence |
| NM_000147.2 | FUCA1 | 1 | Homo sapiens fucosidase, alpha-L- 1, tissue (FUCA1), mRNA. |
| NM_001112732.1 | MCF2L | 13 | Homo sapiens MCF.2 cell line derived transforming sequence-like (MCF2L), transcript variant 1, mRNA. |
| NM_001032287.1 | NR2C1 | 12 | Homo sapiens nuclear receptor subfamily 2, group C, member 1 (NR2C1), transcript variant 2, mRNA. |
| Hs.434957 | HS.434957 | 8 | Homo sapiens, clone IMAGE:3618365, mRNA |
| NM_003580.2 | NSMAF | 8 | Homo sapiens neutral sphingomyelinase (N-SMase) activation associated factor (NSMAF), mRNA. |
| NM_016343.3 | CENPF | 1 | Homo sapiens centromere protein F, 350/400ka (mitosin) (CENPF), mRNA. |
| NM_012118.2 | CCRN4L | 4 | Homo sapiens CCR4 carbon catabolite repression 4-like (S. cerevisiae) (CCRN4L), mRNA. |
| NM_006973.2 | ZNF32 | 10 | Homo sapiens zinc finger protein 32 (ZNF32), transcript variant 2, mRNA. |
| NM_012232.2 | PTRF | 17 | Homo sapiens polymerase I and transcript release factor (PTRF), mRNA. |
| NM_199203.1 | TMEM189-UBE2V1 | 20 | Homo sapiens TMEM189-UBE2V1 readthrough transcript (TMEM189-UBE2V1), transcript variant 1, mRNA. |
| NM_001001660.1 | LYRM5 | 12 | Homo sapiens LYR motif containing 5 (LYRM5), mRNA. |
| NM_014943.3 | ZHX2 | 8 | Homo sapiens zinc fingers and homeoboxes 2 (ZHX2), mRNA. |
| NM_004569.2 | PIGH | 14 | Homo sapiens phosphatidylinositol glycan anchor biosynthesis, class H (PIGH), mRNA. |
| NM_001042581.1 | SNUPN | 15 | Homo sapiens snurportin 1 (SNUPN), transcript variant 2, mRNA. |
| NM_002272.1 | KRT4 | 12 | Homo sapiens keratin 4 (KRT4), mRNA. |
| NM_017953.2 | ZNHIT6 | 1 | Homo sapiens zinc finger, HIT type 6 (ZNHIT6), mRNA. |
| NM_012385.1 | P8 | 16 | Homo sapiens p8 protein (candidate of metastasis 1) (P8), mRNA. |
| NM_001008218.1 | AMY1B | 1 | Homo sapiens amylase, alpha 1B (salivary) (AMY1B), mRNA. |
| NM_006017.1 | PROM1 | 4 | Homo sapiens prominin 1 (PROM1), mRNA. |
| NM_020126.3 | SPHK2 | 19 | Homo sapiens sphingosine kinase 2 (SPHK2), mRNA. |
| NM_000693.1 | ALDH1A3 | 15 | Homo sapiens aldehyde dehydrogenase 1 family, member A3 (ALDH1A3), mRNA. |
| NM_183241.1 | C9ORF142 | 9 | Homo sapiens chromosome 9 open reading frame 142 (C9orf142), mRNA. |
| NM_138699.2 | LOC93622 |  | PREDICTED: Homo sapiens hypothetical protein BC006130 (LOC93622), misc RNA. |
| NM_012218.2 | ILF3 | 19 | Homo sapiens interleukin enhancer binding factor 3, 90kDa (ILF3), transcript variant 1, mRNA. |
| NM_021979.2 | HSPA2 | 14 | Homo sapiens heat shock 70kDa protein 2 (HSPA2), mRNA. |
| NM_002149.2 | HPCAL1 | 2 | Homo sapiens hippocalcin-like 1 (HPCAL1), transcript variant 2, mRNA. |
| NM_016463.5 | CXXC5 | 5 | Homo sapiens CXXC finger 5 (CXXC5), mRNA. |
| NM_025160.4 | WDR26 | 1 | Homo sapiens WD repeat domain 26 (WDR26), mRNA. |
| NM_021953.2 | FOXM1 | 12 | Homo sapiens forkhead box M1 (FOXM1), transcript variant 2, mRNA. |
| NM_006934.2 | SLC6A9 | 1 | Homo sapiens solute carrier family 6 (neurotransmitter transporter, glycine), member 9 (SLC6A9), transcript variant 3, mRNA. |
| NM_002693.1 | POLG | 15 | Homo sapiens polymerase (DNA directed), gamma (POLG), mRNA. |
| NM_016297.2 | PCYOX1 | 2 | Homo sapiens prenylcysteine oxidase 1 (PCYOX1), mRNA. |
| NM_018112.1 | TMEM38B | 9 | Homo sapiens transmembrane protein 38B (TMEM38B), mRNA. |
| NM_018718.1 | TSGA14 | 7 | Homo sapiens testis specific, 14 (TSGA14), mRNA. |
| NM_001014764.1 | TMEM93 | 17 | Homo sapiens transmembrane protein 93 (TMEM93), transcript variant 1, mRNA. |
| NM_004808.1 | NMT2 | 10 | Homo sapiens N-myristoyltransferase 2 (NMT2), mRNA. |
| XM_931864.1 | PLEKHA2 | 8 | Homo sapiens pleckstrin homology domain containing, family A (phosphoinositide binding specific) member 2 (PLEKHA2), mRNA. |
| NM_019050.1 | USP53 | 4 | Homo sapiens ubiquitin specific peptidase 53 (USP53), mRNA. |
| NM_005096.2 | ZMYM3 | X | Homo sapiens zinc finger, MYM-type 3 (ZMYM3), transcript variant 2, mRNA. |
| NM_024321.3 | RBM42 | 19 | Homo sapiens RNA binding motif protein 42 (RBM42), mRNA. |
| NM_014580.3 | SLC2A8 | 9 | Homo sapiens solute carrier family 2 (facilitated glucose transporter), member 8 (SLC2A8), mRNA. |
| NM_006745.3 | SC4MOL | 4 | Homo sapiens sterol-C4-methyl oxidase-like (SC4MOL), transcript variant 2, mRNA. |
| NM_000378.3 | WT1 | 11 | Homo sapiens Wilms tumor 1 (WT1), transcript variant A, mRNA. |
| NM_003971.3 | SPAG9 | 17 | Homo sapiens sperm associated antigen 9 (SPAG9), mRNA. |
| NM_014344.2 | FJX1 | 11 | Homo sapiens four jointed box 1 (Drosophila) (FJX1), mRNA. |
| Hs.355933 | HS.355933 | 5 | Homo sapiens cDNA FLJ41921 fis, clone PERIC2002766 |
| NM_007156.3 | ZXDA | X | Homo sapiens zinc finger, X-linked, duplicated A (ZXDA), mRNA. |
| NM_000120.2 | EPHX1 | 1 | Homo sapiens epoxide hydrolase 1, microsomal (xenobiotic) (EPHX1), mRNA. |
| Hs.19339 | HS.19339 | 3 | Homo sapiens cDNA clone IMAGE:5263177 |
| NM_033551.2 | LARP1 | 5 | Homo sapiens La ribonucleoprotein domain family, member 1 (LARP1), transcript variant 2, mRNA. |
| XM_929667.1 | LOC653778 | 8 | PREDICTED: Homo sapiens similar to solute carrier family 25, member 37 (LOC653778), mRNA. |
| Hs.197076 | HS.197076 | 12 | Homo sapiens cDNA FLJ41747 fis, clone HSYRA2006873 |
| NM_001012993.1 | C9ORF152 | 9 | Homo sapiens chromosome 9 open reading frame 152 (C9orf152), mRNA. |
| XM_943353.1 | LOC648024 |  | PREDICTED: Homo sapiens similar to eukaryotic translation initiation factor 4A, isoform 1 (LOC648024), mRNA. |
| NM_032405.1 | TMPRSS3 | 21 | Homo sapiens transmembrane protease, serine 3 (TMPRSS3), transcript variant D, mRNA. |
| NM_018321.3 | BRIX1 | 5 | Homo sapiens BRX1, biogenesis of ribosomes, homolog (S. cerevisiae) (BRIX1), mRNA. |
| NM_006167.2 | NKX3-1 | 8 | Homo sapiens NK3 homeobox 1 (NKX3-1), mRNA. |
| XM_001717688.1 | LOC100130837 | 10 | PREDICTED: Homo sapiens hypothetical protein LOC100130837 (LOC100130837), mRNA. |
| NM_172070.3 | UBR3 | 2 | Homo sapiens ubiquitin protein ligase E3 component n-recognin 3 (putative) (UBR3), mRNA. |
| XM_290629.6 | C14ORF78 |  | PREDICTED: Homo sapiens chromosome 14 open reading frame 78 (C14orf78), mRNA. |
| NM_005864.2 | EFS | 14 | Homo sapiens embryonal Fyn-associated substrate (EFS), transcript variant 2, mRNA. |
| NM_002676.1 | PMM1 | 22 | Homo sapiens phosphomannomutase 1 (PMM1), mRNA. |
| NM_213636.1 | PDLIM7 | 5 | Homo sapiens PDZ and LIM domain 7 (enigma) (PDLIM7), transcript variant 1, mRNA. |
| NM_001014999.1 | GIYD1 | 16 | Homo sapiens GIY-YIG domain containing 1 (GIYD1), transcript variant 1, mRNA. |
| NM_001123.2 | ADK | 10 | Homo sapiens adenosine kinase (ADK), transcript variant ADK-short, mRNA. |
| NM_001014999.1 | GIYD2 | 16 | Homo sapiens GIY-YIG domain containing 2 (GIYD2), transcript variant 1, mRNA. |
| NM_020378.2 | NAT14 | 19 | Homo sapiens N-acetyltransferase 14 (GCN5-related, putative) (NAT14), mRNA. |
| XM_937514.1 | LOC648470 |  | PREDICTED: Homo sapiens similar to Caspase-4 precursor (CASP-4) (ICH-2 protease) (TX protease) (ICE(rel)-II) (LOC648470), mRNA. |
| NM_003916.3 | AP1S2 | X | Homo sapiens adaptor-related protein complex 1, sigma 2 subunit (AP1S2), mRNA. |
| NM_021643.1 | TRIB2 | 2 | Homo sapiens tribbles homolog 2 (Drosophila) (TRIB2), mRNA. |
| NM_015343.3 | DULLARD | 17 | Homo sapiens dullard homolog (Xenopus laevis) (DULLARD), mRNA. |
| NM_145804.1 | ABTB2 | 11 | Homo sapiens ankyrin repeat and BTB (POZ) domain containing 2 (ABTB2), mRNA. |
| NM_001718.2 | BMP6 | 6 | Homo sapiens bone morphogenetic protein 6 (BMP6), mRNA. |
| NM_003534.2 | HIST1H3G | 6 | Homo sapiens histone cluster 1, H3g (HIST1H3G), mRNA. |
| NM_152523.1 | CCNYL1 | 2 | Homo sapiens cyclin Y-like 1 (CCNYL1), mRNA. |
| NM_175709.2 | CBX7 | 22 | Homo sapiens chromobox homolog 7 (CBX7), mRNA. |
| NM_005674.1 | ZNF239 | 10 | Homo sapiens zinc finger protein 239 (ZNF239), transcript variant 4, mRNA. |
| NM_025081.2 | NYNRIN | 14 | Homo sapiens NYN domain and retroviral integrase containing (NYNRIN), mRNA. |
| NM_032860.3 | LTV1 | 6 | Homo sapiens LTV1 homolog (S. cerevisiae) (LTV1), mRNA. |
| NM_006558.1 | KHDRBS3 | 8 | Homo sapiens KH domain containing, RNA binding, signal transduction associated 3 (KHDRBS3), mRNA. |
| NM_001144.3 | AMFR | 16 | Homo sapiens autocrine motility factor receptor (AMFR), mRNA. |
| NM_003127.1 | SPTAN1 | 9 | Homo sapiens spectrin, alpha, non-erythrocytic 1 (alpha-fodrin) (SPTAN1), mRNA. |
| NM_003690.3 | PRKRA | 2 | Homo sapiens protein kinase, interferon-inducible double stranded RNA dependent activator (PRKRA), mRNA. |
| XM_931683.1 | ISCA1L | 5 | Homo sapiens iron-sulfur cluster assembly 1 homolog (S. cerevisiae)-like (ISCA1L), mRNA. |
| NM_003949.2 | HAP1 | 17 | Homo sapiens huntingtin-associated protein 1 (HAP1), transcript variant 3, mRNA. |
| NM_032578.1 | MYPN | 10 | Homo sapiens myopalladin (MYPN), mRNA. |
| NM_145034.1 | TOR1AIP2 | 1 | Homo sapiens torsin A interacting protein 2 (TOR1AIP2), mRNA. |
| NM_032993.1 | NOLA1 | 4 | Homo sapiens nucleolar protein family A, member 1 (H/ACA small nucleolar RNPs) (NOLA1), transcript variant 2, mRNA. |
| NM_022737.1 | LPPR2 | 19 | Homo sapiens lipid phosphate phosphatase-related protein type 2 (LPPR2), mRNA. |
| NM_005952.2 | MT1X | 16 | Homo sapiens metallothionein 1X (MT1X), mRNA. |
| NM_004907.2 | IER2 | 19 | Homo sapiens immediate early response 2 (IER2), mRNA. |
| NM_138420.2 | AHNAK2 | 14 | Homo sapiens AHNAK nucleoprotein 2 (AHNAK2), mRNA. |
| NR_024438.2 | LOC648740 | 1 | Homo sapiens ACTB pseudogene (LOC648740), non-coding RNA. |
| NM_024702.1 | ZNF750 | 17 | Homo sapiens zinc finger protein 750 (ZNF750), mRNA. |
| NM_005487.3 | HMGXB4 | 22 | Homo sapiens HMG box domain containing 4 (HMGXB4), transcript variant 1, mRNA. |
| NM_001001998.1 | EXOSC10 | 1 | Homo sapiens exosome component 10 (EXOSC10), transcript variant 1, mRNA. |
| NM_012198.2 | GCA | 2 | Homo sapiens grancalcin, EF-hand calcium binding protein (GCA), mRNA. |
| NM_005319.3 | HIST1H1C | 6 | Homo sapiens histone cluster 1, H1c (HIST1H1C), mRNA. |
| NM_006603.3 | STAG2 | X | Homo sapiens stromal antigen 2 (STAG2), transcript variant 2, mRNA. |
| NM_000149.1 | FUT3 | 19 | Homo sapiens fucosyltransferase 3 (galactoside 3(4)-L-fucosyltransferase, Lewis blood group) (FUT3), transcript variant 4, mRNA. |
| NM_203301.1 | FBXO33 | 14 | Homo sapiens F-box protein 33 (FBXO33), mRNA. |
| NM_001675.2 | ATF4 | 22 | Homo sapiens activating transcription factor 4 (tax-responsive enhancer element B67) (ATF4), transcript variant 1, mRNA. |
| NM_032039.1 | ITFG3 | 16 | Homo sapiens integrin alpha FG-GAP repeat containing 3 (ITFG3), mRNA. |
| NM_032667.4 | BSCL2 | 11 | Homo sapiens Bernardinelli-Seip congenital lipodystrophy 2 (seipin) (BSCL2), mRNA. |
| NM_003244.2 | TGIF1 | 18 | Homo sapiens TGFB-induced factor homeobox 1 (TGIF1), transcript variant 4, mRNA. |
| NM_145244.2 | DDIT4L | 4 | Homo sapiens DNA-damage-inducible transcript 4-like (DDIT4L), mRNA. |
| NM_138458.1 | WDR92 | 2 | Homo sapiens WD repeat domain 92 (WDR92), mRNA. |
| NM_012079.2 | DGAT1 | 8 | Homo sapiens diacylglycerol O-acyltransferase homolog 1 (mouse) (DGAT1), mRNA. |
| NM_152504.2 | C20ORF196 | 20 | Homo sapiens chromosome 20 open reading frame 196 (C20orf196), mRNA. |
| NM_002166.4 | ID2 | 2 | Homo sapiens inhibitor of DNA binding 2, dominant negative helix-loop-helix protein (ID2), mRNA. |
| NM_213636.1 | PDLIM7 | 5 | Homo sapiens PDZ and LIM domain 7 (enigma) (PDLIM7), transcript variant 4, mRNA. |
| NM_016605.1 | FAM53C | 5 | Homo sapiens family with sequence similarity 53, member C (FAM53C), mRNA. |
| NM_014882.2 | ARHGAP25 | 2 | Homo sapiens Rho GTPase activating protein 25 (ARHGAP25), transcript variant 2, mRNA. |
| NM_016472.3 | C14ORF129 | 14 | Homo sapiens chromosome 14 open reading frame 129 (C14orf129), mRNA. |
| NM_024325.4 | ZNF343 | 20 | Homo sapiens zinc finger protein 343 (ZNF343), mRNA. |
| NM_016360.2 | TACO1 | 17 | Homo sapiens translational activator of mitochondrially encoded cytochrome c oxidase I (TACO1), nuclear gene encoding mitochondrial protein, mRNA. |
| NM_006145.1 | DNAJB1 | 19 | Homo sapiens DnaJ (Hsp40) homolog, subfamily B, member 1 (DNAJB1), mRNA. |
| NM_023009.4 | MARCKSL1 | 1 | Homo sapiens MARCKS-like 1 (MARCKSL1), mRNA. |
| NM_024320.2 | PRR15L | 17 | Homo sapiens proline rich 15-like (PRR15L), mRNA. |
| NM_000784.2 | CYP27A1 | 2 | Homo sapiens cytochrome P450, family 27, subfamily A, polypeptide 1 (CYP27A1), nuclear gene encoding mitochondrial protein, mRNA. |
| NM_020766.1 | PCDH19 | X | Homo sapiens protocadherin 19 (PCDH19), mRNA. |
| NM_014779.2 | TSC22D2 | 3 | Homo sapiens TSC22 domain family, member 2 (TSC22D2), mRNA. |
| NM_005025.2 | SERPINI1 | 3 | Homo sapiens serpin peptidase inhibitor, clade I (neuroserpin), member 1 (SERPINI1), mRNA. |
| NM_152832.1 | FAM89B | 11 | Homo sapiens family with sequence similarity 89, member B (FAM89B), transcript variant 3, mRNA. |
| NM_199337.1 | LOC374395 | 11 | Homo sapiens similar to RIKEN cDNA 1810059G22 (LOC374395), mRNA. |
| NM_017826.1 | SOHLH2 | 13 | Homo sapiens spermatogenesis and oogenesis specific basic helix-loop-helix 2 (SOHLH2), mRNA. |
| NM_033212.2 | CCDC102A | 16 | Homo sapiens coiled-coil domain containing 102A (CCDC102A), mRNA. |
| NM_007157.3 | ZXDB | X | Homo sapiens zinc finger, X-linked, duplicated B (ZXDB), mRNA. |
| NM_007026.1 | DUSP14 | 17 | Homo sapiens dual specificity phosphatase 14 (DUSP14), mRNA. |
| NM_014815.3 | MED24 | 17 | Homo sapiens mediator complex subunit 24 (MED24), transcript variant 1, mRNA. |
| NM_004428.2 | EFNA1 | 1 | Homo sapiens ephrin-A1 (EFNA1), transcript variant 1, mRNA. |
| NM_199327.1 | LOC730525 |  | PREDICTED: Homo sapiens hypothetical protein LOC730525 (LOC730525), mRNA. |
| NM_005701.2 | SNUPN | 15 | Homo sapiens snurportin 1 (SNUPN), transcript variant 3, mRNA. |
| NM_021220.2 | OVOL2 | 20 | Homo sapiens ovo-like 2 (Drosophila) (OVOL2), mRNA. |
| NM_024097.1 | C1ORF50 | 1 | Homo sapiens chromosome 1 open reading frame 50 (C1orf50), mRNA. |
| Hs.445036 | HS.445036 | 2 | Homo sapiens mRNA; cDNA DKFZp686D22106 (from clone DKFZp686D22106) |
| NM_014506.1 | TOR1B | 9 | Homo sapiens torsin family 1, member B (torsin B) (TOR1B), mRNA. |
| NM_152490.1 | B3GALNT2 | 1 | Homo sapiens beta-1,3-N-acetylgalactosaminyltransferase 2 (B3GALNT2), mRNA. |
| XM_930914.1 | LOC653108 | 21 | PREDICTED: Homo sapiens similar to coxsackie virus and adenovirus receptor precursor (LOC653108), mRNA. |
| XM_001722872.1 | LOC100130919 | X | PREDICTED: Homo sapiens hypothetical protein LOC100130919 (LOC100130919), mRNA. |
| NR_001453.1 | LOC85389 | 11 | Homo sapiens RNA, small nucleolar (LOC85389), non-coding RNA. |
| NM_005053.2 | RAD23A | 19 | Homo sapiens RAD23 homolog A (S. cerevisiae) (RAD23A), mRNA. |
| NM_032324.1 | C1ORF57 | 1 | Homo sapiens chromosome 1 open reading frame 57 (C1orf57), mRNA. |
| NM_021229.2 | NTN4 | 12 | Homo sapiens netrin 4 (NTN4), mRNA. |
| NM_001031726.1 | C19ORF12 | 19 | Homo sapiens chromosome 19 open reading frame 12 (C19orf12), transcript variant 1, mRNA. |
| NM_144591.1 | C10ORF32 | 10 | Homo sapiens chromosome 10 open reading frame 32 (C10orf32), mRNA. |
| NM_015440.3 | MTHFD1L | 6 | Homo sapiens methylenetetrahydrofolate dehydrogenase (NADP+ dependent) 1-like (MTHFD1L), mRNA. |
| NM_153768.1 | CABYR | 18 | Homo sapiens calcium binding tyrosine-(Y)-phosphorylation regulated (CABYR), transcript variant 2, mRNA. |
| NM_182662.1 | AADAT | 4 | Homo sapiens aminoadipate aminotransferase (AADAT), transcript variant 2, mRNA. |
| NM_002114.1 | HIVEP1 | 6 | Homo sapiens human immunodeficiency virus type I enhancer binding protein 1 (HIVEP1), mRNA. |
| NM_198969.1 | AES | 19 | Homo sapiens amino-terminal enhancer of split (AES), transcript variant 2, mRNA. |
| NM_020376.2 | PNPLA2 | 11 | Homo sapiens patatin-like phospholipase domain containing 2 (PNPLA2), mRNA. |
| NM_152527.3 | SLC16A14 | 2 | Homo sapiens solute carrier family 16, member 14 (monocarboxylic acid transporter 14) (SLC16A14), mRNA. |
| NM_024944.2 | CHODL | 21 | Homo sapiens chondrolectin (CHODL), mRNA. |
| NM_030644.1 | APOL3 | 22 | Homo sapiens apolipoprotein L, 3 (APOL3), transcript variant beta/a, mRNA. |
| NM_000018.2 | ACADVL | 17 | Homo sapiens acyl-Coenzyme A dehydrogenase, very long chain (ACADVL), nuclear gene encoding mitochondrial protein, transcript variant 1, mRNA. |
| NM_001080538.1 | AKR1B15 | 7 | Homo sapiens aldo-keto reductase family 1, member B15 (AKR1B15), mRNA. |
| NM_003253.1 | TIAM1 | 21 | Homo sapiens T-cell lymphoma invasion and metastasis 1 (TIAM1), mRNA. |
| NM_021824.2 | NIF3L1 | 2 | Homo sapiens NIF3 NGG1 interacting factor 3-like 1 (S. pombe) (NIF3L1), mRNA. |
| NM_004226.2 | STK17B | 2 | Homo sapiens serine/threonine kinase 17b (STK17B), mRNA. |
| NM_012289.3 | KEAP1 | 19 | Homo sapiens kelch-like ECH-associated protein 1 (KEAP1), transcript variant 2, mRNA. |
| NM_030777.3 | SLC2A10 | 20 | Homo sapiens solute carrier family 2 (facilitated glucose transporter), member 10 (SLC2A10), mRNA. |
| NM_053023.2 | ZFP91 | 11 | Homo sapiens zinc finger protein 91 homolog (mouse) (ZFP91), mRNA. |
| NM_024335.2 | IRX6 | 16 | Homo sapiens iroquois homeobox 6 (IRX6), mRNA. |
| NM_030809.1 | CSRNP2 | 12 | Homo sapiens cysteine-serine-rich nuclear protein 2 (CSRNP2), mRNA. |
| NM_018370.1 | DRAM1 | 12 | Homo sapiens DNA-damage regulated autophagy modulator 1 (DRAM1), mRNA. |
| NM_178835.2 | ZNF827 | 4 | Homo sapiens zinc finger protein 827 (ZNF827), mRNA. |
| NM_138349.2 | TP53I13 | 17 | Homo sapiens tumor protein p53 inducible protein 13 (TP53I13), mRNA. |
| NM_152360.2 | ZNF573 | 19 | Homo sapiens zinc finger protein 573 (ZNF573), mRNA. |
| NM_002916.3 | RFC4 | 3 | Homo sapiens replication factor C (activator 1) 4, 37kDa (RFC4), transcript variant 1, mRNA. |
| NM_002166.4 | ID2 | 2 | Homo sapiens inhibitor of DNA binding 2, dominant negative helix-loop-helix protein (ID2), mRNA. |
| NM_001135255.1 | RBBP4 | 1 | Homo sapiens retinoblastoma binding protein 4 (RBBP4), transcript variant 2, mRNA. |
| NM_177538.1 | CYP20A1 | 2 | Homo sapiens cytochrome P450, family 20, subfamily A, polypeptide 1 (CYP20A1), transcript variant 1, mRNA. |
| XM_942506.1 | ASMTL |  | PREDICTED: Homo sapiens acetylserotonin O-methyltransferase-like (ASMTL), mRNA. |
| XM_945045.1 | LOC649679 |  | PREDICTED: Homo sapiens similar to Tubulin beta-4q chain, transcript variant 2 (LOC649679), mRNA. |
| NM_177417.1 | KLC3 | 19 | Homo sapiens kinesin light chain 3 (KLC3), transcript variant 1, mRNA. |
| NM_182908.3 | DHRS2 | 14 | Homo sapiens dehydrogenase/reductase (SDR family) member 2 (DHRS2), transcript variant 1, mRNA. |
| XM_927722.1 | LOC727848 | 14 | PREDICTED: Homo sapiens similar to actin-like protein (LOC727848), mRNA. |
| NM_152550.2 | SH3RF2 | 5 | Homo sapiens SH3 domain containing ring finger 2 (SH3RF2), mRNA. |
| NM_003315.1 | DNAJC7 | 17 | Homo sapiens DnaJ (Hsp40) homolog, subfamily C, member 7 (DNAJC7), mRNA. |
| NM_198264.1 | FAM189B | 1 | Homo sapiens family with sequence similarity 189, member B (FAM189B), transcript variant 2, mRNA. |
| NM_031488.4 | L3MBTL2 | 22 | Homo sapiens l(3)mbt-like 2 (Drosophila) (L3MBTL2), mRNA. |
| NM_005460.2 | SNCAIP | 5 | Homo sapiens synuclein, alpha interacting protein (SNCAIP), mRNA. |
| NM_017514.2 | PLXNA3 | X | Homo sapiens plexin A3 (PLXNA3), mRNA. |
| Hs.326560 | HS.326560 |  | PREDICTED: Homo sapiens LOC440151 (LOC440151), mRNA |
| NM_021064.3 | HIST1H2AG | 6 | Homo sapiens histone cluster 1, H2ag (HIST1H2AG), mRNA. |
| XM_001715564.1 | LOC100134018 |  | PREDICTED: Homo sapiens similar to hCG1774568 (LOC100134018), mRNA. |
| Hs.534061 | HS.534061 | 17 | full-length cDNA clone XCL0BB001ZD04 of Neuroblastoma of Homo sapiens (human) |
| NM_138426.1 | GLCCI1 | 7 | Homo sapiens glucocorticoid induced transcript 1 (GLCCI1), mRNA. |
| XM_941466.1 | METRNL |  | PREDICTED: Homo sapiens meteorin, glial cell differentiation regulator-like (METRNL), mRNA. |
| NM_031476.1 | CRISPLD2 | 16 | Homo sapiens cysteine-rich secretory protein LCCL domain containing 2 (CRISPLD2), mRNA. |
| NM_002413.3 | MGST2 | 4 | Homo sapiens microsomal glutathione S-transferase 2 (MGST2), mRNA. |
| NR_003716.2 | HOTAIR | 12 | Homo sapiens hox transcript antisense RNA (non-protein coding) (HOTAIR), antisense RNA. |
| NM_014755.1 | SERTAD2 | 2 | Homo sapiens SERTA domain containing 2 (SERTAD2), mRNA. |
| NM_016075.2 | VPS36 | 13 | Homo sapiens vacuolar protein sorting 36 homolog (S. cerevisiae) (VPS36), mRNA. |
| NM_014901.4 | RNF44 | 5 | Homo sapiens ring finger protein 44 (RNF44), mRNA. |
| NM_020251.2 | ARRB1 | 11 | Homo sapiens arrestin, beta 1 (ARRB1), transcript variant 2, mRNA. |
| NM_005540.1 | INPP5B | 1 | Homo sapiens inositol polyphosphate-5-phosphatase, 75kDa (INPP5B), nuclear gene encoding mitochondrial protein, mRNA. |
| NM_000849.3 | GSTM3 | 1 | Homo sapiens glutathione S-transferase M3 (brain) (GSTM3), mRNA. |
| NM_020940.3 | FAM160B1 | 10 | Homo sapiens family with sequence similarity 160, member B1 (FAM160B1), transcript variant 1, mRNA. |
| NM_177963.2 | SYT12 | 11 | Homo sapiens synaptotagmin XII (SYT12), mRNA. |
| NM_002086.3 | GRB2 | 17 | Homo sapiens growth factor receptor-bound protein 2 (GRB2), transcript variant 1, mRNA. |
| NM_017758.2 | ALKBH5 | 17 | Homo sapiens alkB, alkylation repair homolog 5 (E. coli) (ALKBH5), mRNA. |
| NM_020184.2 | CNNM4 | 2 | Homo sapiens cyclin M4 (CNNM4), mRNA. |
| NM_018265.1 | C1ORF106 | 1 | Homo sapiens chromosome 1 open reading frame 106 (C1orf106), mRNA. |
| NM_015907.2 | LAP3 | 4 | Homo sapiens leucine aminopeptidase 3 (LAP3), mRNA. |
| NM_001005376.1 | PLAUR | 19 | Homo sapiens plasminogen activator, urokinase receptor (PLAUR), transcript variant 2, mRNA. |
| NM_004252.1 | SLC9A3R1 | 17 | Homo sapiens solute carrier family 9 (sodium/hydrogen exchanger), member 3 regulator 1 (SLC9A3R1), mRNA. |
| Hs.547277 | HS.547277 | 1 | zh47e08.r1 Soares_fetal_liver_spleen_1NFLS_S1 Homo sapiens cDNA clone IMAGE:415238 5, mRNA sequence |
| NM_003582.2 | DYRK3 | 1 | Homo sapiens dual-specificity tyrosine-(Y)-phosphorylation regulated kinase 3 (DYRK3), transcript variant 1, mRNA. |
| NM_019885.2 | CYP26B1 | 2 | Homo sapiens cytochrome P450, family 26, subfamily B, polypeptide 1 (CYP26B1), mRNA. |
| NM_007111.3 | TFDP1 | 13 | Homo sapiens transcription factor Dp-1 (TFDP1), mRNA. |
| NM_015027.1 | KIAA0251 |  | PREDICTED: Homo sapiens KIAA0251 protein (KIAA0251), mRNA. |
| NM_005095.2 | ZMYM4 | 1 | Homo sapiens zinc finger, MYM-type 4 (ZMYM4), mRNA. |
| Hs.10862 | HS.10862 | 1 | Homo sapiens cDNA: FLJ23313 fis, clone HEP11919 |
| NM_006113.4 | VAV3 | 1 | Homo sapiens vav 3 guanine nucleotide exchange factor (VAV3), transcript variant 1, mRNA. |
| NM_014167.1 | CCDC59 |  | Homo sapiens coiled-coil domain containing 59 (CCDC59), mRNA. |
| NR_006880.1 | SNORD3A | 17 | Homo sapiens small nucleolar RNA, C/D box 3A (SNORD3A), small nucleolar RNA. |
| NM_006113.3 | VAV3 | 1 | Homo sapiens vav 3 guanine nucleotide exchange factor (VAV3), transcript variant 1, mRNA. |
| NM_002053.1 | GBP1 | 1 | Homo sapiens guanylate binding protein 1, interferon-inducible, 67kDa (GBP1), mRNA. |
| Hs.483906 | HS.483906 |  | Homo sapiens mRNA; cDNA DKFZp667L2214 (from clone DKFZp667L2214) |
| NM_005686.2 | SOX13 | 1 | Homo sapiens SRY (sex determining region Y)-box 13 (SOX13), mRNA. |
| NM_014887.1 | PFAAP5 | 13 | Homo sapiens phosphonoformate immuno-associated protein 5 (PFAAP5), mRNA. |
| NM_000754.2 | COMT | 22 | Homo sapiens catechol-O-methyltransferase (COMT), transcript variant S-COMT, mRNA. |
| NM_004281.3 | BAG3 | 10 | Homo sapiens BCL2-associated athanogene 3 (BAG3), mRNA. |
| NM_173794.2 | FUNDC1 | X | Homo sapiens FUN14 domain containing 1 (FUNDC1), mRNA. |
| NM_139276.2 | STAT3 | 17 | Homo sapiens signal transducer and activator of transcription 3 (acute-phase response factor) (STAT3), transcript variant 1, mRNA. |
| NM_016478.3 | ZC3HC1 | 7 | Homo sapiens zinc finger, C3HC-type containing 1 (ZC3HC1), mRNA. |
| NM_005536.2 | IMPA1 | 8 | Homo sapiens inositol(myo)-1(or 4)-monophosphatase 1 (IMPA1), mRNA. |
| NM_006270.2 | RRAS | 19 | Homo sapiens related RAS viral (r-ras) oncogene homolog (RRAS), mRNA. |
| NM_080792.2 | SIRPA | 20 | Homo sapiens signal-regulatory protein alpha (SIRPA), transcript variant 3, mRNA. |
| NM_138768.2 | MYEOV | 11 | Homo sapiens myeloma overexpressed (in a subset of t(11;14) positive multiple myelomas) (MYEOV), mRNA. |
| NM_021734.3 | SLC25A19 | 17 | Homo sapiens solute carrier family 25 (mitochondrial thiamine pyrophosphate carrier), member 19 (SLC25A19), nuclear gene encoding mitochondrial protein, mRNA. |
| NM_152553.2 | RNF217 | 6 | Homo sapiens ring finger protein 217 (RNF217), mRNA. |
| NM_004807.1 | HS6ST1 | 2 | Homo sapiens heparan sulfate 6-O-sulfotransferase 1 (HS6ST1), mRNA. |
| NM_025203.1 | C2ORF44 | 2 | Homo sapiens chromosome 2 open reading frame 44 (C2orf44), mRNA. |
| NM_181873.1 | MTMR11 | 1 | Homo sapiens myotubularin related protein 11 (MTMR11), mRNA. |
| NM_206929.1 | SYTL2 | 11 | Homo sapiens synaptotagmin-like 2 (SYTL2), transcript variant e, mRNA. |
| NM_022367.2 | SEMA4A | 1 | Homo sapiens sema domain, immunoglobulin domain (Ig), transmembrane domain (TM) and short cytoplasmic domain, (semaphorin) 4A (SEMA4A), mRNA. |
| NM_024331.2 | TTPAL | 20 | Homo sapiens tocopherol (alpha) transfer protein-like (TTPAL), transcript variant 2, mRNA. |
| NM_004417.2 | DUSP1 | 5 | Homo sapiens dual specificity phosphatase 1 (DUSP1), mRNA. |
| NM_032737.2 | LMNB2 | 19 | Homo sapiens lamin B2 (LMNB2), mRNA. |
| XM_943555.1 | LOC728229 | 22 | PREDICTED: Homo sapiens hypothetical protein LOC728229, transcript variant 3 (LOC728229), mRNA. |
| NM_002070.2 | GNAI2 | 3 | Homo sapiens guanine nucleotide binding protein (G protein), alpha inhibiting activity polypeptide 2 (GNAI2), transcript variant 1, mRNA. |
| NM_020645.1 | NRIP3 | 11 | Homo sapiens nuclear receptor interacting protein 3 (NRIP3), mRNA. |
| NR_024392.1 | HSPBL2 | 9 | Homo sapiens heat shock 27kDa protein-like 2 pseudogene (HSPBL2), non-coding RNA. |
| NM_001037293.1 | PALM2 | 9 | Homo sapiens paralemmin 2 (PALM2), transcript variant 2, mRNA. |
| NM_173515.2 | CNKSR3 | 6 | Homo sapiens CNKSR family member 3 (CNKSR3), mRNA. |
| XM_940575.1 | MAP2K3 | 17 | Homo sapiens mitogen-activated protein kinase kinase 3 (MAP2K3), transcript variant A, mRNA. |
| NM_016423.1 | ZNF219 | 14 | Homo sapiens zinc finger protein 219 (ZNF219), mRNA. |
| NM_001417.2 | EIF4B | 12 | Homo sapiens eukaryotic translation initiation factor 4B (EIF4B), mRNA. |
| NM_006241.3 | PPP1R2 | 3 | Homo sapiens protein phosphatase 1, regulatory (inhibitor) subunit 2 (PPP1R2), mRNA. |
| NM_032448.1 | FAM120B | 6 | Homo sapiens family with sequence similarity 120B (FAM120B), mRNA. |
| NM_133476.2 | ZNF384 | 12 | Homo sapiens zinc finger protein 384 (ZNF384), transcript variant 2, mRNA. |
| NM_005308.2 | GRK5 | 10 | Homo sapiens G protein-coupled receptor kinase 5 (GRK5), mRNA. |
| NM_032132.2 | HORMAD1 | 1 | Homo sapiens HORMA domain containing 1 (HORMAD1), mRNA. |
| NM_014330.2 | PPP1R15A | 19 | Homo sapiens protein phosphatase 1, regulatory (inhibitor) subunit 15A (PPP1R15A), mRNA. |
| NM_014153.2 | ZC3H7A | 16 | Homo sapiens zinc finger CCCH-type containing 7A (ZC3H7A), mRNA. |
| NM_014448.2 | ARHGEF16 | 1 | Homo sapiens Rho guanine exchange factor (GEF) 16 (ARHGEF16), mRNA. |
| NM_004564.1 | PET112L | 4 | Homo sapiens PET112-like (yeast) (PET112L), mRNA. |
| NM_004651.3 | USP11 | X | Homo sapiens ubiquitin specific peptidase 11 (USP11), mRNA. |
| NM_006899.2 | IDH3B | 20 | Homo sapiens isocitrate dehydrogenase 3 (NAD+) beta (IDH3B), nuclear gene encoding mitochondrial protein, transcript variant 1, mRNA. |
| NM_054014.1 | FKBP1A | 20 | Homo sapiens FK506 binding protein 1A, 12kDa (FKBP1A), transcript variant 12A, mRNA. |
| NM_033386.2 | MICALL1 | 22 | Homo sapiens MICAL-like 1 (MICALL1), mRNA. |
| NM_001259.5 | CDK6 | 7 | Homo sapiens cyclin-dependent kinase 6 (CDK6), mRNA. |
| NM_030674.3 | SLC38A1 | 12 | Homo sapiens solute carrier family 38, member 1 (SLC38A1), transcript variant 1, mRNA. |
| NM_182826.1 | SCARA3 | 8 | Homo sapiens scavenger receptor class A, member 3 (SCARA3), transcript variant 2, mRNA. |
| NM_005685.2 | GTF2IRD1 | 7 | Homo sapiens GTF2I repeat domain containing 1 (GTF2IRD1), transcript variant 1, mRNA. |
| NM_004990.2 | MARS | 12 | Homo sapiens methionyl-tRNA synthetase (MARS), mRNA. |
| NM_001078.2 | VCAM1 | 1 | Homo sapiens vascular cell adhesion molecule 1 (VCAM1), transcript variant 1, mRNA. |
| NM_033452.2 | TRIM47 | 17 | Homo sapiens tripartite motif-containing 47 (TRIM47), mRNA. |
| Hs.535028 | HS.535028 | 7 | Homo sapiens cDNA: FLJ22720 fis, clone HSI14320 |
| XM_931359.1 | LOC338758 | 12 | PREDICTED: Homo sapiens hypothetical protein LOC338758 (LOC338758), mRNA. |
| NM_012475.4 | USP21 | 1 | Homo sapiens ubiquitin specific peptidase 21 (USP21), transcript variant 1, mRNA. |
| NM_004239.1 | TRIP11 | 14 | Homo sapiens thyroid hormone receptor interactor 11 (TRIP11), mRNA. |
| NR_001454.1 | LOC85390 | 11 | Homo sapiens RNA, small nucleolar (LOC85390), non-coding RNA. |
| NM_021979.2 | HSPA2 | 14 | Homo sapiens heat shock 70kDa protein 2 (HSPA2), mRNA. |
| NM_001806.2 | CEBPG | 19 | Homo sapiens CCAAT/enhancer binding protein (C/EBP), gamma (CEBPG), mRNA. |
| NM_022652.2 | DUSP6 | 12 | Homo sapiens dual specificity phosphatase 6 (DUSP6), transcript variant 2, mRNA. |
| NM_006479.2 | RAD51AP1 | 12 | Homo sapiens RAD51 associated protein 1 (RAD51AP1), mRNA. |
| NM_005793.3 | NME6 | 3 | Homo sapiens non-metastatic cells 6, protein expressed in (nucleoside-diphosphate kinase) (NME6), mRNA. |
| XM_926881.1 | GNA12 | 7 | Homo sapiens guanine nucleotide binding protein (G protein) alpha 12 (GNA12), mRNA. |
| NM_152493.2 | ZNF362 | 1 | Homo sapiens zinc finger protein 362 (ZNF362), mRNA. |
| NM_004818.2 | DDX23 | 12 | Homo sapiens DEAD (Asp-Glu-Ala-Asp) box polypeptide 23 (DDX23), mRNA. |
| NM_018389.3 | SLC35C1 | 11 | Homo sapiens solute carrier family 35, member C1 (SLC35C1), mRNA. |
| NR_002207.1 | CSNK2A1P | 11 | Homo sapiens casein kinase 2, alpha 1 polypeptide pseudogene (CSNK2A1P), non-coding RNA. |
| NM_005862.2 | STAG1 | 3 | Homo sapiens stromal antigen 1 (STAG1), mRNA. |
| NR_024510.1 | LOC728855 | 1 | Homo sapiens hypothetical LOC728855 (LOC728855), non-coding RNA. |
| NM_199444.1 | COPE | 19 | Homo sapiens coatomer protein complex, subunit epsilon (COPE), transcript variant 2, mRNA. |
| NM_032117.2 | MND1 | 4 | Homo sapiens meiotic nuclear divisions 1 homolog (S. cerevisiae) (MND1), mRNA. |
| NM_001002257.1 | LCLAT1 | 2 | Homo sapiens lysocardiolipin acyltransferase 1 (LCLAT1), transcript variant 2, mRNA. |
| XM_001723978.1 | SPNS2 |  | PREDICTED: Homo sapiens spinster homolog 2 (Drosophila) (SPNS2), mRNA. |
| NM_006005.2 | WFS1 | 4 | Homo sapiens Wolfram syndrome 1 (wolframin) (WFS1), mRNA. |
| NM_002601.2 | PDE6D | 2 | Homo sapiens phosphodiesterase 6D, cGMP-specific, rod, delta (PDE6D), mRNA. |
| XR_038101.1 | LOC100129237 |  | PREDICTED: Homo sapiens misc_RNA (LOC100129237), miscRNA. |
| NM_033102.1 | SLC45A3 | 1 | Homo sapiens solute carrier family 45, member 3 (SLC45A3), mRNA. |
| NM_003789.2 | TRADD | 16 | Homo sapiens TNFRSF1A-associated via death domain (TRADD), mRNA. |
| NM_020909.2 | EPB41L5 | 2 | Homo sapiens erythrocyte membrane protein band 4.1 like 5 (EPB41L5), mRNA. |
| NM_006893.2 | LGTN | 1 | Homo sapiens ligatin (LGTN), mRNA. |
| XM_940079.1 | TUBB6 |  | PREDICTED: Homo sapiens tubulin, beta 6 (TUBB6), mRNA. |
| NM_145034.1 | TOR1AIP2 | 1 | Homo sapiens torsin A interacting protein 2 (TOR1AIP2), mRNA. |
| NM_024107.1 | TMUB2 | 17 | Homo sapiens transmembrane and ubiquitin-like domain containing 2 (TMUB2), transcript variant 3, mRNA. |
| NM_144988.2 | ALG14 | 1 | Homo sapiens asparagine-linked glycosylation 14 homolog (S. cerevisiae) (ALG14), mRNA. |
| NM_031458.1 | PARP9 | 3 | Homo sapiens poly (ADP-ribose) polymerase family, member 9 (PARP9), mRNA. |
| NM_032578.1 | MYPN | 10 | Homo sapiens myopalladin (MYPN), mRNA. |
| NM_017582.5 | UBE2Q1 | 1 | Homo sapiens ubiquitin-conjugating enzyme E2Q (putative) 1 (UBE2Q1), mRNA. |
| NM_007317.1 | KIF22 | 16 | Homo sapiens kinesin family member 22 (KIF22), mRNA. |
| NM_006706.3 | TCERG1 | 5 | Homo sapiens transcription elongation regulator 1 (TCERG1), transcript variant 1, mRNA. |
| NM_194325.1 | ZNF30 | 19 | Homo sapiens zinc finger protein 30 (ZNF30), transcript variant 2, mRNA. |
| NM_022163.2 | MRPL46 | 15 | Homo sapiens mitochondrial ribosomal protein L46 (MRPL46), nuclear gene encoding mitochondrial protein, mRNA. |
| NM_030583.2 | MATN2 | 8 | Homo sapiens matrilin 2 (MATN2), transcript variant 1, mRNA. |
| NM_003689.2 | AKR7A2 | 1 | Homo sapiens aldo-keto reductase family 7, member A2 (aflatoxin aldehyde reductase) (AKR7A2), mRNA. |
| NM_024815.2 | NUDT18 | 8 | Homo sapiens nudix (nucleoside diphosphate linked moiety X)-type motif 18 (NUDT18), mRNA. |
| NM_012067.2 | AKR7A3 | 1 | Homo sapiens aldo-keto reductase family 7, member A3 (aflatoxin aldehyde reductase) (AKR7A3), mRNA. |
| NM_182470.1 | PKM2 | 15 | Homo sapiens pyruvate kinase, muscle (PKM2), transcript variant 2, mRNA. |
| NM_024618.2 | NLRX1 | 11 | Homo sapiens NLR family member X1 (NLRX1), transcript variant 2, mRNA. |
| NM_152391.2 | PQLC3 | 2 | Homo sapiens PQ loop repeat containing 3 (PQLC3), mRNA. |
| NM_001003931.1 | PARP3 | 3 | Homo sapiens poly (ADP-ribose) polymerase family, member 3 (PARP3), transcript variant 1, mRNA. |
| NM_024710.1 | ISOC2 | 19 | Homo sapiens isochorismatase domain containing 2 (ISOC2), mRNA. |
| NM_001236.3 | CBR3 | 21 | Homo sapiens carbonyl reductase 3 (CBR3), mRNA. |
| NM_006984.3 | CLDN10 | 13 | Homo sapiens claudin 10 (CLDN10), transcript variant 2, mRNA. |
| NM_013390.1 | TMEM2 | 9 | Homo sapiens transmembrane protein 2 (TMEM2), mRNA. |
| NM_001024630.1 | RUNX2 | 6 | Homo sapiens runt-related transcription factor 2 (RUNX2), transcript variant 1, mRNA. |
| NM_001001870.1 | C17ORF91 | 17 | Homo sapiens chromosome 17 open reading frame 91 (C17orf91), transcript variant 2, mRNA. |
| NM_145305.1 | SLC25A43 | X | Homo sapiens solute carrier family 25, member 43 (SLC25A43), mRNA. |
| NM_001921.2 | DCTD | 4 | Homo sapiens dCMP deaminase (DCTD), transcript variant 2, mRNA. |
| NM_003541.2 | HIST1H4K | 6 | Homo sapiens histone cluster 1, H4k (HIST1H4K), mRNA. |
| NM_019008.4 | SMCR7L | 22 | Homo sapiens Smith-Magenis syndrome chromosome region, candidate 7-like (SMCR7L), mRNA. |
| NM_003768.2 | PEA15 | 1 | Homo sapiens phosphoprotein enriched in astrocytes 15 (PEA15), mRNA. |
| NM_178867.3 | SFXN4 | 10 | Homo sapiens sideroflexin 4 (SFXN4), mRNA. |
| NM_022343.2 | GLIPR2 | 9 | Homo sapiens GLI pathogenesis-related 2 (GLIPR2), mRNA. |
| NM_005564.2 | LCN2 | 9 | Homo sapiens lipocalin 2 (LCN2), mRNA. |
| NM_147173.1 | NUDT2 | 9 | Homo sapiens nudix (nucleoside diphosphate linked moiety X)-type motif 2 (NUDT2), transcript variant 3, mRNA. |
| NM_175744.4 | RHOC | 1 | Homo sapiens ras homolog gene family, member C (RHOC), transcript variant 1, mRNA. |
| NM_003243.2 | TGFBR3 | 1 | Homo sapiens transforming growth factor, beta receptor III (TGFBR3), mRNA. |
| NM_181785.1 | SLC46A3 | 13 | Homo sapiens solute carrier family 46, member 3 (SLC46A3), mRNA. |
| NM_000927.3 | ABCB1 | 7 | Homo sapiens ATP-binding cassette, sub-family B (MDR/TAP), member 1 (ABCB1), mRNA. |
| NM_152890.4 | COL24A1 | 1 | Homo sapiens collagen, type XXIV, alpha 1 (COL24A1), mRNA. |
| NR_002987.1 | SNORA61 | 1 | Homo sapiens small nucleolar RNA, H/ACA box 61 (SNORA61), small nucleolar RNA. |
| NM_024959.2 | SLC24A6 | 12 | Homo sapiens solute carrier family 24 (sodium/potassium/calcium exchanger), member 6 (SLC24A6), mRNA. |
| NM_021127.1 | PMAIP1 | 18 | Homo sapiens phorbol-12-myristate-13-acetate-induced protein 1 (PMAIP1), mRNA. |
| NM_001149.2 | ANK3 | 10 | Homo sapiens ankyrin 3, node of Ranvier (ankyrin G) (ANK3), transcript variant 2, mRNA. |
| XR_042330.1 | LOC729009 | 2 | PREDICTED: Homo sapiens misc_RNA (LOC729009), miscRNA. |
| NM_033089.6 | ZCCHC3 | 20 | Homo sapiens zinc finger, CCHC domain containing 3 (ZCCHC3), mRNA. |
| NM_017735.3 | TTC27 | 2 | Homo sapiens tetratricopeptide repeat domain 27 (TTC27), mRNA. |
| NM_207352.2 | CYP4V2 | 4 | Homo sapiens cytochrome P450, family 4, subfamily V, polypeptide 2 (CYP4V2), mRNA. |
| NM_144770.2 | RBM11 | 21 | Homo sapiens RNA binding motif protein 11 (RBM11), mRNA. |
| NM_001018837.1 | HAX1 | 1 | Homo sapiens HCLS1 associated protein X-1 (HAX1), transcript variant 1, mRNA. |
| NM_001031738.1 | TMEM150A | 2 | Homo sapiens transmembrane protein 150A (TMEM150A), transcript variant 1, mRNA. |
| Hs.13262 | LOC439949 |  | PREDICTED: Homo sapiens hypothetical gene supported by AY007155 (LOC439949), mRNA. |
| NM_181353.1 | ID1 | 20 | Homo sapiens inhibitor of DNA binding 1, dominant negative helix-loop-helix protein (ID1), transcript variant 2, mRNA. |
| NM_001031628.1 | SMAGP | 12 | Homo sapiens small cell adhesion glycoprotein (SMAGP), transcript variant 1, mRNA. |
| NM_012460.2 | TIMM9 | 14 | Homo sapiens translocase of inner mitochondrial membrane 9 homolog (yeast) (TIMM9), nuclear gene encoding mitochondrial protein, mRNA. |
| XM_936269.1 | WDR74 |  | PREDICTED: Homo sapiens WD repeat domain 74 (WDR74), mRNA. |
| NM_015958.1 | DPH5 | 1 | Homo sapiens DPH5 homolog (S. cerevisiae) (DPH5), transcript variant 1, mRNA. |
| NM_017988.4 | SCYL2 | 12 | Homo sapiens SCY1-like 2 (S. cerevisiae) (SCYL2), mRNA. |
| NM_024551.2 | ADIPOR2 | 12 | Homo sapiens adiponectin receptor 2 (ADIPOR2), mRNA. |
| NM_030800.1 | C15ORF44 | 15 | Homo sapiens chromosome 15 open reading frame 44 (C15orf44), transcript variant 2, mRNA. |
| Hs.150067 | HS.150067 | 1 | Homo sapiens cDNA FLJ43367 fis, clone NT2RP8000435 |
| NM_000155.2 | GALT | 9 | Homo sapiens galactose-1-phosphate uridylyltransferase (GALT), mRNA. |
| NM_020315.3 | PDXP | 22 | Homo sapiens pyridoxal (pyridoxine, vitamin B6) phosphatase (PDXP), mRNA. |
| NM_181800.1 | UBE2C | 20 | Homo sapiens ubiquitin-conjugating enzyme E2C (UBE2C), transcript variant 3, mRNA. |
| NM_002105.2 | LOC731314 |  | PREDICTED: Homo sapiens similar to H2A histone family, member X (LOC731314), mRNA. |
| NM_006401.1 | ANP32B | 9 | Homo sapiens acidic (leucine-rich) nuclear phosphoprotein 32 family, member B (ANP32B), mRNA. |
| NM_025128.3 | MUS81 | 11 | Homo sapiens MUS81 endonuclease homolog (S. cerevisiae) (MUS81), mRNA. |
| NM_001006610.1 | SIAH1 | 16 | Homo sapiens seven in absentia homolog 1 (Drosophila) (SIAH1), transcript variant 2, mRNA. |
| NM_003124.3 | SPR | 2 | Homo sapiens sepiapterin reductase (7,8-dihydrobiopterin:NADP+ oxidoreductase) (SPR), mRNA. |
| NM_015318.2 | ARHGEF18 | 19 | Homo sapiens rho/rac guanine nucleotide exchange factor (GEF) 18 (ARHGEF18), mRNA. |
| XM_926019.1 | LOC727848 | 14 | PREDICTED: Homo sapiens similar to actin-like protein (LOC727848), mRNA. |
| NM_014140.2 | SMARCAL1 | 2 | Homo sapiens SWI/SNF related, matrix associated, actin dependent regulator of chromatin, subfamily a-like 1 (SMARCAL1), mRNA. |
| NM_016114.3 | ASB1 | 2 | Homo sapiens ankyrin repeat and SOCS box-containing 1 (ASB1), mRNA. |
| NM_130898.2 | CREB3L4 | 1 | Homo sapiens cAMP responsive element binding protein 3-like 4 (CREB3L4), mRNA. |
| NM_138369.1 | FAM44B | 5 | Homo sapiens family with sequence similarity 44, member B (FAM44B), mRNA. |
| XM_929476.1 | ZNF630 | X | PREDICTED: Homo sapiens zinc finger protein 630, transcript variant 2 (ZNF630), mRNA. |
| NM_177543.1 | PPAP2C | 19 | Homo sapiens phosphatidic acid phosphatase type 2C (PPAP2C), transcript variant 2, mRNA. |
| NM_004075.2 | CRY1 | 12 | Homo sapiens cryptochrome 1 (photolyase-like) (CRY1), mRNA. |
| NM_003825.2 | SNAP23 | 15 | Homo sapiens synaptosomal-associated protein, 23kDa (SNAP23), transcript variant 1, mRNA. |
| NM_004645.2 | COIL | 17 | Homo sapiens coilin (COIL), mRNA. |
| NM_024319.2 | C1ORF35 | 1 | Homo sapiens chromosome 1 open reading frame 35 (C1orf35), mRNA. |
| NM_013442.1 | STOML2 | 9 | Homo sapiens stomatin (EPB72)-like 2 (STOML2), mRNA. |
| XM_940732.1 | LOC732007 |  | PREDICTED: Homo sapiens similar to Phosphoglycerate mutase 1 (Phosphoglycerate mutase isozyme B) (PGAM-B) (BPG-dependent PGAM 1) (LOC732007), mRNA. |
| NM_006366.2 | CAP2 | 6 | Homo sapiens CAP, adenylate cyclase-associated protein, 2 (yeast) (CAP2), mRNA. |
| NM_021831.5 | AGBL5 | 2 | Homo sapiens ATP/GTP binding protein-like 5 (AGBL5), transcript variant 1, mRNA. |
| NM_198401.2 | ANKRD46 | 8 | Homo sapiens ankyrin repeat domain 46 (ANKRD46), mRNA. |
| NM_172373.2 | ELF1 | 13 | Homo sapiens E74-like factor 1 (ets domain transcription factor) (ELF1), mRNA. |
| NM_021177.3 | LSM2 | 6 | Homo sapiens LSM2 homolog, U6 small nuclear RNA associated (S. cerevisiae) (LSM2), mRNA. |
| XM_928410.1 | LOC651849 |  | PREDICTED: Homo sapiens similar to MARCKS-related protein (MARCKS-like protein 1) (Macrophage myristoylated alanine-rich C kinase substrate) (Mac-MARCKS) (MacMARCKS) (LOC651849), mRNA. |
| NM_024866.3 | ADM2 | 22 | Homo sapiens adrenomedullin 2 (ADM2), mRNA. |
| XM_937928.1 | LOC347376 |  | PREDICTED: Homo sapiens similar to H3 histone, family 3B (LOC347376), mRNA. |
| NM_153219.2 | ZNF524 | 19 | Homo sapiens zinc finger protein 524 (ZNF524), mRNA. |
| NM_006591.1 | POLD3 | 11 | Homo sapiens polymerase (DNA-directed), delta 3, accessory subunit (POLD3), mRNA. |
| NM_020904.1 | PLEKHA4 | 19 | Homo sapiens pleckstrin homology domain containing, family A (phosphoinositide binding specific) member 4 (PLEKHA4), mRNA. |
| NM_016256.2 | NAGPA | 16 | Homo sapiens N-acetylglucosamine-1-phosphodiester alpha-N-acetylglucosaminidase (NAGPA), mRNA. |
| NM_003579.2 | RAD54L | 1 | Homo sapiens RAD54-like (S. cerevisiae) (RAD54L), mRNA. |
| XR_015564.1 | LOC729495 | 22 | PREDICTED: Homo sapiens similar to hCG1999863 (LOC729495), mRNA. |
| NM_018110.2 | DOK4 | 16 | Homo sapiens docking protein 4 (DOK4), mRNA. |
| NR_001444.1 | LYPLA2P1 |  | Homo sapiens lysophospholipase II pseudogene 1 (LYPLA2P1) on chromosome 6. |
| NM_144576.2 | COQ10A | 12 | Homo sapiens coenzyme Q10 homolog A (S. cerevisiae) (COQ10A), transcript variant 1, mRNA. |
| NM_080668.2 | CDCA5 | 11 | Homo sapiens cell division cycle associated 5 (CDCA5), mRNA. |
| NM_198566.1 | C5ORF34 | 5 | Homo sapiens chromosome 5 open reading frame 34 (C5orf34), mRNA. |
| NM_018367.5 | ACER3 | 11 | Homo sapiens alkaline ceramidase 3 (ACER3), mRNA. |
| NM_178502.2 | DTX3 | 12 | Homo sapiens deltex homolog 3 (Drosophila) (DTX3), mRNA. |
| NM_023930.2 | KCTD14 | 11 | Homo sapiens potassium channel tetramerisation domain containing 14 (KCTD14), mRNA. |
| NM_006399.2 | BATF | 14 | Homo sapiens basic leucine zipper transcription factor, ATF-like (BATF), mRNA. |
| NM_018410.2 | HJURP | 2 | Homo sapiens Holliday junction recognition protein (HJURP), mRNA. |
| NM_014177.1 | C18ORF55 | 18 | Homo sapiens chromosome 18 open reading frame 55 (C18orf55), mRNA. |
| NM_153715.1 | HOXA10 | 7 | Homo sapiens homeobox A10 (HOXA10), transcript variant 1, mRNA. |
| NM_198541.1 | IGFL1 | 19 | Homo sapiens IGF-like family member 1 (IGFL1), mRNA. |
| XM_941466.1 | METRNL |  | PREDICTED: Homo sapiens meteorin, glial cell differentiation regulator-like (METRNL), mRNA. |
| NM_006567.2 | FARS2 | 6 | Homo sapiens phenylalanyl-tRNA synthetase 2, mitochondrial (FARS2), nuclear gene encoding mitochondrial protein, mRNA. |
| NM_080651.1 | MED30 | 8 | Homo sapiens mediator complex subunit 30 (MED30), mRNA. |
| NM_194272.1 | RBPMS2 | 15 | Homo sapiens RNA binding protein with multiple splicing 2 (RBPMS2), mRNA. |
| NM_000418.2 | IL4R | 16 | Homo sapiens interleukin 4 receptor (IL4R), transcript variant 1, mRNA. |
| NM_020841.4 | OSBPL8 | 12 | Homo sapiens oxysterol binding protein-like 8 (OSBPL8), transcript variant 1, mRNA. |
| NM_020168.3 | PAK6 | 15 | Homo sapiens p21(CDKN1A)-activated kinase 6 (PAK6), mRNA. |
| NM_001018021.1 | MUC1 | 1 | Homo sapiens mucin 1, cell surface associated (MUC1), transcript variant 6, mRNA. |
| NM_014920.2 | ICK | 6 | Homo sapiens intestinal cell (MAK-like) kinase (ICK), transcript variant 2, mRNA. |
| NM_005646.2 | TARBP1 | 1 | Homo sapiens TAR (HIV-1) RNA binding protein 1 (TARBP1), mRNA. |
| NM_005516.4 | HLA-E | 6 | Homo sapiens major histocompatibility complex, class I, E (HLA-E), mRNA. |
| NM_145176.1 | SLC2A12 | 6 | Homo sapiens solute carrier family 2 (facilitated glucose transporter), member 12 (SLC2A12), mRNA. |
| NM_014071.2 | NCOA6 | 20 | Homo sapiens nuclear receptor coactivator 6 (NCOA6), mRNA. |
| NR_015380.1 | NCRNA00181 | 19 | Homo sapiens non-protein coding RNA 181 (NCRNA00181), non-coding RNA. |
| NM_001012479.1 | GRN | 17 | Homo sapiens granulin (GRN), mRNA. |
| NM_153705.2 | KDELC2 | 11 | Homo sapiens KDEL (Lys-Asp-Glu-Leu) containing 2 (KDELC2), mRNA. |
| NM_015537.3 | NELF | 9 | Homo sapiens nasal embryonic LHRH factor (NELF), mRNA. |
| NM_001003818.1 | TRIM6 | 11 | Homo sapiens tripartite motif-containing 6 (TRIM6), transcript variant 2, mRNA. |
| NM_001859.2 | SLC31A1 | 9 | Homo sapiens solute carrier family 31 (copper transporters), member 1 (SLC31A1), mRNA. |
| NM_001005742.1 | GBA | 1 | Homo sapiens glucosidase, beta; acid (includes glucosylceramidase) (GBA), transcript variant 3, mRNA. |
| NM_001753.3 | CAV1 | 7 | Homo sapiens caveolin 1, caveolae protein, 22kDa (CAV1), mRNA. |
| NM_004117.2 | FKBP5 | 6 | Homo sapiens FK506 binding protein 5 (FKBP5), mRNA. |
| NM_014254.1 | TMEM5 | 12 | Homo sapiens transmembrane protein 5 (TMEM5), mRNA. |
| NM_030808.3 | NDEL1 | 17 | Homo sapiens nudE nuclear distribution gene E homolog (A. nidulans)-like 1 (NDEL1), transcript variant 2, mRNA. |
| NM_002685.2 | EXOSC10 | 1 | Homo sapiens exosome component 10 (EXOSC10), transcript variant 1, mRNA. |
| NM_032346.1 | PDCD2L | 19 | Homo sapiens programmed cell death 2-like (PDCD2L), mRNA. |
| NM_014223.2 | NFYC | 1 | Homo sapiens nuclear transcription factor Y, gamma (NFYC), mRNA. |
| NM_005110.1 | GFPT2 | 5 | Homo sapiens glutamine-fructose-6-phosphate transaminase 2 (GFPT2), mRNA. |
| NM_003459.4 | SLC30A3 | 2 | Homo sapiens solute carrier family 30 (zinc transporter), member 3 (SLC30A3), mRNA. |
| NR_002315.1 | LOC440926 | 2 | Homo sapiens H3 histone, family 3A pseudogene (LOC440926), non-coding RNA. |
| NM_015161.1 | ARL6IP1 | 16 | Homo sapiens ADP-ribosylation factor-like 6 interacting protein 1 (ARL6IP1), mRNA. |
| NM_018047.1 | RBM22 | 5 | Homo sapiens RNA binding motif protein 22 (RBM22), mRNA. |
| NM_002275.2 | KRT15 | 17 | Homo sapiens keratin 15 (KRT15), mRNA. |
| NM_001693.2 | ATP6V1B2 | 8 | Homo sapiens ATPase, H+ transporting, lysosomal 56/58kDa, V1 subunit B2 (ATP6V1B2), mRNA. |
| NM_014829.2 | DDX46 | 5 | Homo sapiens DEAD (Asp-Glu-Ala-Asp) box polypeptide 46 (DDX46), mRNA. |
| NM_004808.2 | NMT2 | 10 | Homo sapiens N-myristoyltransferase 2 (NMT2), mRNA. |
| NM_139242.2 | MTFMT | 15 | Homo sapiens mitochondrial methionyl-tRNA formyltransferase (MTFMT), mRNA. |
| NM_003915.2 | CPNE1 | 20 | Homo sapiens copine I (CPNE1), transcript variant 3, mRNA. |
| NM_031902.3 | MRPS5 | 2 | Homo sapiens mitochondrial ribosomal protein S5 (MRPS5), nuclear gene encoding mitochondrial protein, mRNA. |
| NM_016338.3 | IPO11 | 5 | Homo sapiens importin 11 (IPO11), mRNA. |
| NM_004035.4 | ACOX1 | 17 | Homo sapiens acyl-Coenzyme A oxidase 1, palmitoyl (ACOX1), transcript variant 2, mRNA. |
| NM_004865.2 | TBPL1 | 6 | Homo sapiens TBP-like 1 (TBPL1), mRNA. |
| NM_032813.1 | TMTC4 | 13 | Homo sapiens transmembrane and tetratricopeptide repeat containing 4 (TMTC4), transcript variant 2, mRNA. |
| NR_024618.1 | LOC100129550 | 3 | Homo sapiens hypothetical LOC100129550 (LOC100129550), non-coding RNA. |
| NM_002960.1 | S100A3 | 1 | Homo sapiens S100 calcium binding protein A3 (S100A3), mRNA. |
| NM_213650.1 | SFXN4 | 10 | Homo sapiens sideroflexin 4 (SFXN4), transcript variant 3, mRNA. |
| NM_018449.2 | UBAP2 | 9 | Homo sapiens ubiquitin associated protein 2 (UBAP2), mRNA. |
| NM_014943.3 | ZHX2 | 8 | Homo sapiens zinc fingers and homeoboxes 2 (ZHX2), mRNA. |
| NM_001660.2 | ARF4 | 3 | Homo sapiens ADP-ribosylation factor 4 (ARF4), mRNA. |
| NM_174930.2 | PMS2L5 | 7 | Homo sapiens postmeiotic segregation increased 2-like 5 (PMS2L5), mRNA. |
| NM_014881.2 | DCLRE1A | 10 | Homo sapiens DNA cross-link repair 1A (PSO2 homolog, S. cerevisiae) (DCLRE1A), mRNA. |
| NM_003971.3 | SPAG9 | 17 | Homo sapiens sperm associated antigen 9 (SPAG9), mRNA. |
| Hs.368255 | KIAA0368 | 9 | Homo sapiens KIAA0368 (KIAA0368), mRNA. |
| NM_018700.3 | TRIM36 | 5 | Homo sapiens tripartite motif-containing 36 (TRIM36), transcript variant 1, mRNA. |
| XM_001713901.1 | LOC391769 |  | PREDICTED: Homo sapiens similar to HIStone family member (his-72) (LOC391769), mRNA. |
| XM_001723141.1 | LOC100131261 |  | PREDICTED: Homo sapiens similar to hCG1728885 (LOC100131261), mRNA. |
| NM_012197.2 | RABGAP1 | 9 | Homo sapiens RAB GTPase activating protein 1 (RABGAP1), mRNA. |
| NM_001950.3 | E2F4 | 16 | Homo sapiens E2F transcription factor 4, p107/p130-binding (E2F4), mRNA. |
| NM_033168.2 | B3GALNT1 | 3 | Homo sapiens beta-1,3-N-acetylgalactosaminyltransferase 1 (globoside blood group) (B3GALNT1), transcript variant 3, mRNA. |
| NM_001554.3 | CYR61 | 1 | Homo sapiens cysteine-rich, angiogenic inducer, 61 (CYR61), mRNA. |
| NM_030919.1 | FAM83D | 20 | Homo sapiens family with sequence similarity 83, member D (FAM83D), mRNA. |
| NR_003098.1 | SNHG1 | 11 | Homo sapiens small nucleolar RNA host gene 1 (non-protein coding) (SNHG1), non-coding RNA. |
| NM_133436.1 | ASNS | 7 | Homo sapiens asparagine synthetase (ASNS), transcript variant 1, mRNA. |
| NM_173552.2 | C3ORF58 | 3 | Homo sapiens chromosome 3 open reading frame 58 (C3orf58), mRNA. |
| NM_005368.2 | MB | 22 | Homo sapiens myoglobin (MB), transcript variant 1, mRNA. |
| NM_015062.3 | PPRC1 | 10 | Homo sapiens peroxisome proliferator-activated receptor gamma, coactivator-related 1 (PPRC1), mRNA. |
| NM_012288.3 | TRAM2 | 6 | Homo sapiens translocation associated membrane protein 2 (TRAM2), mRNA. |
| NM_206930.1 | SYTL2 | 11 | Homo sapiens synaptotagmin-like 2 (SYTL2), transcript variant d, mRNA. |
| NM_033631.2 | LUZP1 | 1 | Homo sapiens leucine zipper protein 1 (LUZP1), mRNA. |
| NM_005895.2 | GOLGA3 | 12 | Homo sapiens golgi autoantigen, golgin subfamily a, 3 (GOLGA3), mRNA. |
| NM_003730.3 | RNASET2 | 6 | Homo sapiens ribonuclease T2 (RNASET2), mRNA. |
| NM_023937.2 | MRPL34 | 19 | Homo sapiens mitochondrial ribosomal protein L34 (MRPL34), nuclear gene encoding mitochondrial protein, mRNA. |
| NM_016048.1 | ISOC1 | 5 | Homo sapiens isochorismatase domain containing 1 (ISOC1), mRNA. |
| NR_023343.1 | RNU4ATAC | 2 | Homo sapiens RNA, U4atac small nuclear (U12-dependent splicing) (RNU4ATAC), small nuclear RNA. |
| NM_024520.1 | C2ORF47 | 2 | Homo sapiens chromosome 2 open reading frame 47 (C2orf47), mRNA. |
| NM_032376.2 | TMEM101 | 17 | Homo sapiens transmembrane protein 101 (TMEM101), mRNA. |
| NM_001101417.2 | LOC729920 |  | Homo sapiens notch1-induced protein (LOC729920), transcript variant 2, mRNA. |
| NM_006750.2 | SNTB2 | 16 | Homo sapiens syntrophin, beta 2 (dystrophin-associated protein A1, 59kDa, basic component 2) (SNTB2), transcript variant 1, mRNA. |
| NM_000216.1 | KAL1 | X | Homo sapiens Kallmann syndrome 1 sequence (KAL1), mRNA. |
| NM_003620.2 | PPM1D | 17 | Homo sapiens protein phosphatase 1D magnesium-dependent, delta isoform (PPM1D), mRNA. |
| NM_000402.3 | G6PD | X | Homo sapiens glucose-6-phosphate dehydrogenase (G6PD), transcript variant 1, mRNA. |
| XM_001126659.2 | LOC728026 | 9 | PREDICTED: Homo sapiens hypothetical LOC728026 (LOC728026), mRNA. |
| NM_019891.2 | ERO1LB | 1 | Homo sapiens ERO1-like beta (S. cerevisiae) (ERO1LB), mRNA. |
| NM_022463.3 | NXN | 17 | Homo sapiens nucleoredoxin (NXN), mRNA. |
| NM_181519.2 | SYT15 | 10 | Homo sapiens synaptotagmin XV (SYT15), transcript variant b, mRNA. |
| NM_014324.4 | AMACR | 5 | Homo sapiens alpha-methylacyl-CoA racemase (AMACR), transcript variant 1, mRNA. |
| NM_032581.2 | FAM126A | 7 | Homo sapiens family with sequence similarity 126, member A (FAM126A), mRNA. |
| NM_138463.2 | TLCD1 | 17 | Homo sapiens TLC domain containing 1 (TLCD1), mRNA. |
| NM_006096.2 | NDRG1 | 8 | Homo sapiens N-myc downstream regulated gene 1 (NDRG1), mRNA. |
| NM_005557.2 | KRT16 | 17 | Homo sapiens keratin 16 (focal non-epidermolytic palmoplantar keratoderma) (KRT16), mRNA. |
| NM_033657.1 | LOC645676 | 1 | PREDICTED: Homo sapiens hypothetical protein LOC645676, transcript variant 1 (LOC645676), mRNA. |
| XM_932071.1 | LOC120376 |  | PREDICTED: Homo sapiens hypothetical protein LOC120376 (LOC120376), mRNA. |
| NM_001521.1 | GTF3C2 | 2 | Homo sapiens general transcription factor IIIC, polypeptide 2, beta 110kDa (GTF3C2), transcript variant 1, mRNA. |
| NM_032319.1 | C2ORF7 | 2 | Homo sapiens chromosome 2 open reading frame 7 (C2orf7), mRNA. |
| NM_002355.2 | M6PR | 12 | Homo sapiens mannose-6-phosphate receptor (cation dependent) (M6PR), mRNA. |
| NM_052932.1 | TMEM123 | 11 | Homo sapiens transmembrane protein 123 (TMEM123), mRNA. |
| NR_023921.1 | C14ORF167 | 14 | Homo sapiens chromosome 14 open reading frame 167 (C14orf167), transcript variant 1, non-coding RNA. |
| NM_020401.2 | NUP107 | 12 | Homo sapiens nucleoporin 107kDa (NUP107), mRNA. |
| NM_001040021.1 | CD14 | 5 | Homo sapiens CD14 molecule (CD14), transcript variant 2, mRNA. |
| NM_000779.2 | CYP4B1 | 1 | Homo sapiens cytochrome P450, family 4, subfamily B, polypeptide 1 (CYP4B1), mRNA. |
| NM_173039.1 | AQP11 | 11 | Homo sapiens aquaporin 11 (AQP11), mRNA. |
| NM_000430.2 | PAFAH1B1 | 17 | Homo sapiens platelet-activating factor acetylhydrolase, isoform Ib, alpha subunit 45kDa (PAFAH1B1), mRNA. |
| NM_018109.2 | PAPD1 | 10 | Homo sapiens PAP associated domain containing 1 (PAPD1), mRNA. |
| NM_030805.1 | LMAN2L | 2 | Homo sapiens lectin, mannose-binding 2-like (LMAN2L), mRNA. |
| NM_000819.3 | GART | 21 | Homo sapiens phosphoribosylglycinamide formyltransferase, phosphoribosylglycinamide synthetase, phosphoribosylaminoimidazole synthetase (GART), transcript variant 1, mRNA. |
| XM_945505.1 | ATP1B3 |  | PREDICTED: Homo sapiens ATPase, Na+/K+ transporting, beta 3 polypeptide, transcript variant 2 (ATP1B3), mRNA. |
| NM_019083.1 | CCDC76 | 1 | Homo sapiens coiled-coil domain containing 76 (CCDC76), mRNA. |
| NM_004489.4 | GPS2 | 17 | Homo sapiens G protein pathway suppressor 2 (GPS2), mRNA. |
| NM_004055.3 | CAPN5 | 11 | Homo sapiens calpain 5 (CAPN5), mRNA. |
| NM_032818.1 | C9ORF100 | 9 | Homo sapiens chromosome 9 open reading frame 100 (C9orf100), transcript variant 2, mRNA. |
| NM_001993.2 | F3 | 1 | Homo sapiens coagulation factor III (thromboplastin, tissue factor) (F3), mRNA. |
| NM_004356.3 | CD81 | 11 | Homo sapiens CD81 molecule (CD81), mRNA. |
| NM_032305.1 | POLR3GL | 1 | Homo sapiens polymerase (RNA) III (DNA directed) polypeptide G (32kD)-like (POLR3GL), mRNA. |
| NM_024775.9 | GEMIN6 | 2 | Homo sapiens gem (nuclear organelle) associated protein 6 (GEMIN6), mRNA. |
| NM_016538.1 | SIRT7 | 17 | Homo sapiens sirtuin (silent mating type information regulation 2 homolog) 7 (S. cerevisiae) (SIRT7), mRNA. |
| NM_016397.2 | TH1L | 20 | Homo sapiens TH1-like (Drosophila) (TH1L), transcript variant 2, mRNA. |
| NM_001358.2 | DHX15 | 4 | Homo sapiens DEAH (Asp-Glu-Ala-His) box polypeptide 15 (DHX15), mRNA. |
| NM_020747.1 | ZNF608 | 5 | Homo sapiens zinc finger protein 608 (ZNF608), mRNA. |
| NM_003680.2 | YARS | 1 | Homo sapiens tyrosyl-tRNA synthetase (YARS), mRNA. |
| NM_005675.2 | DGCR6 | 22 | Homo sapiens DiGeorge syndrome critical region gene 6 (DGCR6), mRNA. |
| NM_017996.2 | DET1 | 15 | Homo sapiens de-etiolated homolog 1 (Arabidopsis) (DET1), mRNA. |
| NM_177964.3 | LYPD6B | 2 | Homo sapiens LY6/PLAUR domain containing 6B (LYPD6B), mRNA. |
| NM_006724.2 | MAP3K4 | 6 | Homo sapiens mitogen-activated protein kinase kinase kinase 4 (MAP3K4), transcript variant 1, mRNA. |
| NM_015936.1 | YARS2 | 12 | Homo sapiens tyrosyl-tRNA synthetase 2, mitochondrial (YARS2), nuclear gene encoding mitochondrial protein, mRNA. |
| Hs.556018 | HS.556018 | 6 | Homo sapiens mRNA; cDNA DKFZp779F0411 (from clone DKFZp779F0411) |
| NM_021101.3 | CLDN1 | 3 | Homo sapiens claudin 1 (CLDN1), mRNA. |
| NM_024101.4 | MLPH | 2 | Homo sapiens melanophilin (MLPH), transcript variant 2, mRNA. |
| NM_016823.2 | CRK | 17 | Homo sapiens v-crk sarcoma virus CT10 oncogene homolog (avian) (CRK), transcript variant II, mRNA. |
| NR_002912.1 | SNORA67 | 17 | Homo sapiens small nucleolar RNA, H/ACA box 67 (SNORA67), small nucleolar RNA. |
| NM_013237.2 | PRELID1 | 5 | Homo sapiens PRELI domain containing 1 (PRELID1), mRNA. |
| Hs.311428 | HS.311428 | 2 | Homo sapiens cDNA FLJ14199 fis, clone NT2RP3002713 |
| XM_001725603.1 | LOC100128252 | 19 | PREDICTED: Homo sapiens similar to MGC9913 protein (LOC100128252), mRNA. |
| XM_379668.3 | LOC286208 | 9 | PREDICTED: Homo sapiens hypothetical protein LOC286208, transcript variant 1 (LOC286208), mRNA. |
| NM_144603.2 | NOXO1 | 16 | Homo sapiens NADPH oxidase organizer 1 (NOXO1), transcript variant b, mRNA. |
| NM_177925.1 | H2AFJ | 12 | Homo sapiens H2A histone family, member J (H2AFJ), transcript variant 1, mRNA. |
| NM_207362.2 | C2ORF55 | 2 | Homo sapiens chromosome 2 open reading frame 55 (C2orf55), mRNA. |
| NM_003223.1 | TFAP4 | 16 | Homo sapiens transcription factor AP-4 (activating enhancer binding protein 4) (TFAP4), mRNA. |
| XM_928117.1 | LOC729252 | 17 | PREDICTED: Homo sapiens similar to Keratin, type I cytoskeletal 14 (Cytokeratin-14) (CK-14) (Keratin-14) (K14) (LOC729252), mRNA. |
| NM_014056.1 | HIGD1A | 3 | Homo sapiens HIG1 domain family, member 1A (HIGD1A), mRNA. |
| NM_024661.2 | CCDC51 | 3 | Homo sapiens coiled-coil domain containing 51 (CCDC51), mRNA. |
| NM_003597.4 | KLF11 |  | PREDICTED: Homo sapiens Kruppel-like factor 11 (KLF11), mRNA. |
| NM_015690.2 | STK36 | 2 | Homo sapiens serine/threonine kinase 36, fused homolog (Drosophila) (STK36), mRNA. |
| NM_024844.2 | NUP85 | 17 | Homo sapiens nucleoporin 85kDa (NUP85), mRNA. |
| NM_000169.1 | GLA | X | Homo sapiens galactosidase, alpha (GLA), mRNA. |
| NM_007144.2 | PCGF2 | 17 | Homo sapiens polycomb group ring finger 2 (PCGF2), mRNA. |
| NM_015535.1 | SPATS2L | 2 | Homo sapiens spermatogenesis associated, serine-rich 2-like (SPATS2L), transcript variant 2, mRNA. |
| NM_018293.1 | ZNF654 | 3 | Homo sapiens zinc finger protein 654 (ZNF654), mRNA. |
| NM_172037.2 | RDH10 | 8 | Homo sapiens retinol dehydrogenase 10 (all-trans) (RDH10), mRNA. |
| NM_012479.2 | YWHAG | 7 | Homo sapiens tyrosine 3-monooxygenase/tryptophan 5-monooxygenase activation protein, gamma polypeptide (YWHAG), mRNA. |
| XM_942503.1 | FAM69B |  | PREDICTED: Homo sapiens family with sequence similarity 69, member B (FAM69B), mRNA. |
| NM_015057.2 | MYCBP2 | 13 | Homo sapiens MYC binding protein 2 (MYCBP2), mRNA. |
| Hs.381058 | KIAA0146 | 8 | Homo sapiens KIAA0146 (KIAA0146), mRNA. |
| NM_018353.3 | C14ORF106 | 14 | Homo sapiens chromosome 14 open reading frame 106 (C14orf106), mRNA. |
| NM_024952.4 | C14ORF159 |  | Homo sapiens chromosome 14 open reading frame 159 (C14orf159), mRNA. |
| NM_006527.2 | SLBP | 4 | Homo sapiens stem-loop binding protein (SLBP), mRNA. |
| NM_017946.2 | FKBP14 | 7 | Homo sapiens FK506 binding protein 14, 22 kDa (FKBP14), mRNA. |
| NM_012393.1 | PFAS | 17 | Homo sapiens phosphoribosylformylglycinamidine synthase (FGAR amidotransferase) (PFAS), mRNA. |
| NM_016391.2 | HSPC111 | 5 | Homo sapiens hypothetical protein HSPC111 (HSPC111), mRNA. |
| NM_145810.1 | CDCA7 | 2 | Homo sapiens cell division cycle associated 7 (CDCA7), transcript variant 1, mRNA. |
| NM_079837.1 | BANP | 16 | Homo sapiens BTG3 associated nuclear protein (BANP), transcript variant 2, mRNA. |
| NM_152373.2 | ZNF684 | 1 | Homo sapiens zinc finger protein 684 (ZNF684), mRNA. |
| NM_206914.1 | FAM119B | 12 | Homo sapiens family with sequence similarity 119, member B (FAM119B), transcript variant 1, mRNA. |
| NM_012214.1 | MGAT4A | 2 | Homo sapiens mannosyl (alpha-1,3-)-glycoprotein beta-1,4-N-acetylglucosaminyltransferase, isozyme A (MGAT4A), transcript variant 1, mRNA. |
| NM_001003786.1 | STRADA | 17 | Homo sapiens STE20-related kinase adaptor alpha (STRADA), transcript variant 2, mRNA. |
| NM_181558.1 | RFC3 | 13 | Homo sapiens replication factor C (activator 1) 3, 38kDa (RFC3), transcript variant 1, mRNA. |
| NM_201525.1 | GPR56 | 16 | Homo sapiens G protein-coupled receptor 56 (GPR56), transcript variant 3, mRNA. |
| NM_003152.2 | STAT5A | 17 | Homo sapiens signal transducer and activator of transcription 5A (STAT5A), mRNA. |
| NM_024792.1 | FAM57A | 17 | Homo sapiens family with sequence similarity 57, member A (FAM57A), mRNA. |
| NM_144590.1 | ANKRD22 | 10 | Homo sapiens ankyrin repeat domain 22 (ANKRD22), mRNA. |
| NM_025265.2 | TSEN2 | 3 | Homo sapiens tRNA splicing endonuclease 2 homolog (S. cerevisiae) (TSEN2), mRNA. |
| NM_004139.2 | LBP | 20 | Homo sapiens lipopolysaccharide binding protein (LBP), mRNA. |
| XM_001717411.1 | LOC100133430 |  | PREDICTED: Homo sapiens similar to hCG1640620 (LOC100133430), mRNA. |
| NM_014384.2 | ACAD8 | 11 | Homo sapiens acyl-Coenzyme A dehydrogenase family, member 8 (ACAD8), nuclear gene encoding mitochondrial protein, mRNA. |
| NM_020375.1 | C12ORF5 | 12 | Homo sapiens chromosome 12 open reading frame 5 (C12orf5), mRNA. |
| NM_000159.2 | GCDH | 19 | Homo sapiens glutaryl-Coenzyme A dehydrogenase (GCDH), nuclear gene encoding mitochondrial protein, transcript variant 1, mRNA. |
| NM_203417.1 | RCAN1 | 21 | Homo sapiens regulator of calcineurin 1 (RCAN1), transcript variant 2, mRNA. |
| NM_004083.4 | DDIT3 | 12 | Homo sapiens DNA-damage-inducible transcript 3 (DDIT3), mRNA. |
| NR_023920.1 | WIT1 | 11 | Homo sapiens Wilms tumor upstream neighbor 1 (WIT1), non-coding RNA. |
| NM_003400.3 | XPO1 | 2 | Homo sapiens exportin 1 (CRM1 homolog, yeast) (XPO1), mRNA. |
| NM_139353.1 | TAF1C | 16 | Homo sapiens TATA box binding protein (TBP)-associated factor, RNA polymerase I, C, 110kDa (TAF1C), transcript variant 2, mRNA. |
| NM_024638.2 | QTRTD1 | 3 | Homo sapiens queuine tRNA-ribosyltransferase domain containing 1 (QTRTD1), mRNA. |
| NM_003434.3 | ZNF133 | 20 | Homo sapiens zinc finger protein 133 (ZNF133), mRNA. |
| NM_016257.2 | HPCAL4 | 1 | Homo sapiens hippocalcin like 4 (HPCAL4), mRNA. |
| NM_014766.2 | SCRN1 | 7 | Homo sapiens secernin 1 (SCRN1), mRNA. |
| NM_173691.1 | C9ORF75 | 9 | Homo sapiens chromosome 9 open reading frame 75 (C9orf75), mRNA. |
| NM_005836.2 | HRSP12 | 8 | Homo sapiens heat-responsive protein 12 (HRSP12), mRNA. |
| NM_016821.1 | OGG1 | 3 | Homo sapiens 8-oxoguanine DNA glycosylase (OGG1), nuclear gene encoding mitochondrial protein, transcript variant 2a, mRNA. |
| NM_002830.2 | PTPN4 | 2 | Homo sapiens protein tyrosine phosphatase, non-receptor type 4 (megakaryocyte) (PTPN4), mRNA. |
| NM_152379.2 | C1ORF131 | 1 | Homo sapiens chromosome 1 open reading frame 131 (C1orf131), mRNA. |
| NM_015855.2 | WIT-1 | 11 | Homo sapiens Wilms tumor associated protein (WIT-1), mRNA. |
| NM_015002.2 | FBXO21 | 12 | Homo sapiens F-box protein 21 (FBXO21), transcript variant 2, mRNA. |
| NM_001005741.1 | GBA | 1 | Homo sapiens glucosidase, beta; acid (includes glucosylceramidase) (GBA), transcript variant 3, mRNA. |
| NM_199420.2 | POLQ | 3 | Homo sapiens polymerase (DNA directed), theta (POLQ), mRNA. |
| NM_013238.2 | DNAJC15 | 13 | Homo sapiens DnaJ (Hsp40) homolog, subfamily C, member 15 (DNAJC15), mRNA. |
| NM_002659.2 | PLAUR | 19 | Homo sapiens plasminogen activator, urokinase receptor (PLAUR), transcript variant 1, mRNA. |
| Hs.576633 | HS.576633 | 7 | BX447862 Homo sapiens T CELLS (JURKAT CELL LINE) Homo sapiens cDNA clone CS0DH002YG04 3-PRIME, mRNA sequence |
| NM_018944.2 | C21ORF45 | 21 | Homo sapiens chromosome 21 open reading frame 45 (C21orf45), mRNA. |
| NM_005861.2 | STUB1 | 16 | Homo sapiens STIP1 homology and U-box containing protein 1 (STUB1), mRNA. |
| NM_015161.1 | ARL6IP1 | 16 | Homo sapiens ADP-ribosylation factor-like 6 interacting protein 1 (ARL6IP1), mRNA. |
| NM_001630.2 | ANXA8L2 | 10 | Homo sapiens annexin A8-like 2 (ANXA8L2), mRNA. |
| NM_181504.2 | PIK3R1 | 5 | Homo sapiens phosphoinositide-3-kinase, regulatory subunit 1 (alpha) (PIK3R1), transcript variant 2, mRNA. |
| NM_007170.1 | TESK2 | 1 | Homo sapiens testis-specific kinase 2 (TESK2), mRNA. |
| NM_033426.2 | KIAA1737 | 14 | Homo sapiens KIAA1737 (KIAA1737), mRNA. |
| XM_938340.1 | STX5 | 11 | Homo sapiens syntaxin 5 (STX5), mRNA. |
| NM_001031718.1 | GDPD3 | 16 | Homo sapiens glycerophosphodiester phosphodiesterase domain containing 3 (GDPD3), mRNA. |
| NM_002836.2 | PTPRA | 20 | Homo sapiens protein tyrosine phosphatase, receptor type, A (PTPRA), transcript variant 1, mRNA. |
| NM_018186.2 | C1ORF112 | 1 | Homo sapiens chromosome 1 open reading frame 112 (C1orf112), mRNA. |
| NM_018957.2 | SH3BP1 | 22 | Homo sapiens SH3-domain binding protein 1 (SH3BP1), mRNA. |
| NM_213646.1 | WARS | 14 | Homo sapiens tryptophanyl-tRNA synthetase (WARS), transcript variant 2, mRNA. |
| NM_006101.1 | NDC80 | 18 | Homo sapiens NDC80 homolog, kinetochore complex component (S. cerevisiae) (NDC80), mRNA. |
| NM_007266.1 | GPN1 | 2 | Homo sapiens GPN-loop GTPase 1 (GPN1), mRNA. |
| NM_001172.3 | ARG2 | 14 | Homo sapiens arginase, type II (ARG2), nuclear gene encoding mitochondrial protein, mRNA. |
| NM_032345.1 | WIBG | 12 | Homo sapiens within bgcn homolog (Drosophila) (WIBG), mRNA. |
| NM_001789.2 | CDC25A | 3 | Homo sapiens cell division cycle 25 homolog A (S. pombe) (CDC25A), transcript variant 1, mRNA. |
| NM_006067.3 | COX4NB | 16 | Homo sapiens COX4 neighbor (COX4NB), mRNA. |
| NM_017754.3 | UHRF1BP1 | 6 | Homo sapiens UHRF1 binding protein 1 (UHRF1BP1), mRNA. |
| NM_015913.2 | TXNDC12 | 1 | Homo sapiens thioredoxin domain containing 12 (endoplasmic reticulum) (TXNDC12), mRNA. |
| NM_002915.2 | RFC3 | 13 | Homo sapiens replication factor C (activator 1) 3, 38kDa (RFC3), transcript variant 1, mRNA. |
| NM_005744.2 | ARIH1 | 15 | Homo sapiens ariadne homolog, ubiquitin-conjugating enzyme E2 binding protein, 1 (Drosophila) (ARIH1), mRNA. |
| NM_030665.3 | RAI1 | 17 | Homo sapiens retinoic acid induced 1 (RAI1), mRNA. |
| NM_018234.2 | STEAP3 | 2 | Homo sapiens STEAP family member 3 (STEAP3), transcript variant 2, mRNA. |
| NM_022447.1 | PAPD5 | 16 | Homo sapiens PAP associated domain containing 5 (PAPD5), transcript variant 2, mRNA. |
| NM_044472.1 | CDC42 | 1 | Homo sapiens cell division cycle 42 (GTP binding protein, 25kDa) (CDC42), transcript variant 3, mRNA. |
| NM_005134.2 | PPP4R1 | 18 | Homo sapiens protein phosphatase 4, regulatory subunit 1 (PPP4R1), transcript variant 2, mRNA. |
| NM_177538.1 | CYP20A1 | 2 | Homo sapiens cytochrome P450, family 20, subfamily A, polypeptide 1 (CYP20A1), mRNA. |
| NM_018845.1 | RAG1AP1 | 1 | Homo sapiens recombination activating gene 1 activating protein 1 (RAG1AP1), mRNA. |
| NM_207170.1 | SYF2 | 1 | Homo sapiens SYF2 homolog, RNA splicing factor (S. cerevisiae) (SYF2), transcript variant 1, mRNA. |
| NM_032772.3 | ZNF503 | 10 | Homo sapiens zinc finger protein 503 (ZNF503), mRNA. |
| NM_002294.1 | LAMP2 | X | Homo sapiens lysosomal-associated membrane protein 2 (LAMP2), transcript variant LAMP2A, mRNA. |
| NM_006848.2 | CCDC85B | 11 | Homo sapiens coiled-coil domain containing 85B (CCDC85B), mRNA. |
| NM_005557.2 | KRT16 | 17 | Homo sapiens keratin 16 (focal non-epidermolytic palmoplantar keratoderma) (KRT16), mRNA. |
| NM_016463.7 | CXXC5 | 5 | Homo sapiens CXXC finger 5 (CXXC5), mRNA. |
| NM_017968.2 | SLTM | 15 | Homo sapiens SAFB-like, transcription modulator (SLTM), transcript variant 1, mRNA. |
| XM_001726158.1 | LOC730167 |  | PREDICTED: Homo sapiens similar to protein tyrosine phosphatase 4a1, transcript variant 1 (LOC730167), mRNA. |
| XM_001721047.1 | LOC100132139 | 9 | PREDICTED: Homo sapiens similar to methylenetetrahydrofolate dehydrogenase (NADP+ dependent) 1-like (LOC100132139), mRNA. |
| NM_144583.2 | ATP6V1C2 | 2 | Homo sapiens ATPase, H+ transporting, lysosomal 42kDa, V1 subunit C2 (ATP6V1C2), transcript variant 2, mRNA. |
| NM_015496.3 | KIAA1429 | 8 | Homo sapiens KIAA1429 (KIAA1429), transcript variant 1, mRNA. |
| NM_001018115.1 | FANCD2 | 3 | Homo sapiens Fanconi anemia, complementation group D2 (FANCD2), transcript variant 2, mRNA. |
| NM_002201.4 | ISG20 | 15 | Homo sapiens interferon stimulated exonuclease gene 20kDa (ISG20), mRNA. |
| NM_023077.1 | C1ORF163 | 1 | Homo sapiens chromosome 1 open reading frame 163 (C1orf163), mRNA. |
| NM_078487.2 | CDKN2B | 9 | Homo sapiens cyclin-dependent kinase inhibitor 2B (p15, inhibits CDK4) (CDKN2B), transcript variant 1, mRNA. |
| NM_004897.2 | MINPP1 | 10 | Homo sapiens multiple inositol polyphosphate histidine phosphatase, 1 (MINPP1), mRNA. |
| NM_017592.1 | MED29 | 19 | Homo sapiens mediator complex subunit 29 (MED29), mRNA. |
| NM_019886.2 | CHST7 | X | Homo sapiens carbohydrate (N-acetylglucosamine 6-O) sulfotransferase 7 (CHST7), mRNA. |
| NM_016575.1 | NT5DC3 | 12 | Homo sapiens 5'-nucleotidase domain containing 3 (NT5DC3), transcript variant 2, mRNA. |
| NM_182557.1 | BCL9L | 11 | Homo sapiens B-cell CLL/lymphoma 9-like (BCL9L), mRNA. |
| NM_032857.2 | LACTB | 15 | Homo sapiens lactamase, beta (LACTB), nuclear gene encoding mitochondrial protein, transcript variant 1, mRNA. |
| NM_013242.2 | C16ORF80 | 16 | Homo sapiens chromosome 16 open reading frame 80 (C16orf80), mRNA. |
| NM_007187.3 | WBP4 | 13 | Homo sapiens WW domain binding protein 4 (formin binding protein 21) (WBP4), mRNA. |
| NM_145117.3 | NAV2 | 11 | Homo sapiens neuron navigator 2 (NAV2), transcript variant 2, mRNA. |
| NM_005858.2 | AKAP8 | 19 | Homo sapiens A kinase (PRKA) anchor protein 8 (AKAP8), mRNA. |
| NM_001033030.1 | FAIM | 3 | Homo sapiens Fas apoptotic inhibitory molecule (FAIM), transcript variant 1, mRNA. |
| NM_014247.2 | RAPGEF2 | 4 | Homo sapiens Rap guanine nucleotide exchange factor (GEF) 2 (RAPGEF2), mRNA. XM_944403 XM_944410 XM_944412 |
| NM_006759.3 | UGP2 | 2 | Homo sapiens UDP-glucose pyrophosphorylase 2 (UGP2), transcript variant 1, mRNA. |
| NM_152387.2 | KCTD18 | 2 | Homo sapiens potassium channel tetramerisation domain containing 18 (KCTD18), mRNA. |
| NM_025083.2 | EDC3 | 15 | Homo sapiens enhancer of mRNA decapping 3 homolog (S. cerevisiae) (EDC3), mRNA. |
| NM_030665.3 | RAI1 | 17 | Homo sapiens retinoic acid induced 1 (RAI1), mRNA. |
| NR_002756.1 | RNU5A | 15 | Homo sapiens RNA, U5A small nuclear (RNU5A), small nuclear RNA. |
| NM_001954.3 | DDR1 | 6 | Homo sapiens discoidin domain receptor tyrosine kinase 1 (DDR1), transcript variant 1, mRNA. |
| NM_022817.1 | PER2 | 2 | Homo sapiens period homolog 2 (Drosophila) (PER2), mRNA. |
| NM_015534.3 | ZZZ3 | 1 | Homo sapiens zinc finger, ZZ-type containing 3 (ZZZ3), mRNA. |
| NM_018518.3 | MCM10 | 10 | Homo sapiens minichromosome maintenance complex component 10 (MCM10), transcript variant 2, mRNA. |
| NM_003621.1 | PPFIBP2 | 11 | Homo sapiens PTPRF interacting protein, binding protein 2 (liprin beta 2) (PPFIBP2), mRNA. |
| NM_024646.1 | ZYG11B | 1 | Homo sapiens zyg-11 homolog B (C. elegans) (ZYG11B), mRNA. |
| NM_016014.2 | FAM108B1 | 9 | Homo sapiens family with sequence similarity 108, member B1 (FAM108B1), transcript variant 1, mRNA. |
| NM_001007157.1 | PHF14 | 7 | Homo sapiens PHD finger protein 14 (PHF14), transcript variant 1, mRNA. |
| NM_004855.3 | PIGB | 15 | Homo sapiens phosphatidylinositol glycan anchor biosynthesis, class B (PIGB), mRNA. |
| NM_001077188.1 | HS6ST2 | X | Homo sapiens heparan sulfate 6-O-sulfotransferase 2 (HS6ST2), transcript variant L, mRNA. |
| NM_145183.1 | PYCARD | 16 | Homo sapiens PYD and CARD domain containing (PYCARD), transcript variant 1, mRNA. |
| NM_001012756.1 | ZNF260 | 19 | Homo sapiens zinc finger protein 260 (ZNF260), mRNA. |
| NM_018719.2 | CDCA7L | 7 | Homo sapiens cell division cycle associated 7-like (CDCA7L), mRNA. |
| NM_007027.2 | TOPBP1 | 3 | Homo sapiens topoisomerase (DNA) II binding protein 1 (TOPBP1), mRNA. |
| NM_006612.3 | KIF1C | 17 | Homo sapiens kinesin family member 1C (KIF1C), mRNA. |
| NM_003846.1 | PEX11B | 1 | Homo sapiens peroxisomal biogenesis factor 11 beta (PEX11B), mRNA. |
| NM_153742.3 | CTH | 1 | Homo sapiens cystathionase (cystathionine gamma-lyase) (CTH), transcript variant 2, mRNA. |
| NM_004111.4 | FEN1 | 11 | Homo sapiens flap structure-specific endonuclease 1 (FEN1), mRNA. |
| NM_153026.1 | PRICKLE1 | 12 | Homo sapiens prickle homolog 1 (Drosophila) (PRICKLE1), mRNA. |
| NM_018715.1 | RCC2 | 1 | Homo sapiens regulator of chromosome condensation 2 (RCC2), mRNA. |
| NM_014226.1 | RAGE | 14 | Homo sapiens renal tumor antigen (RAGE), mRNA. |
| XM_938887.1 | KLF11 |  | PREDICTED: Homo sapiens Kruppel-like factor 11 (KLF11), mRNA. |
| NM_001017917.1 | CYB561 | 17 | Homo sapiens cytochrome b-561 (CYB561), transcript variant 3, mRNA. |
| NM_004569.3 | PIGH | 14 | Homo sapiens phosphatidylinositol glycan anchor biosynthesis, class H (PIGH), mRNA. |
| NM_014316.1 | CARHSP1 | 16 | Homo sapiens calcium regulated heat stable protein 1, 24kDa (CARHSP1), transcript variant 2, mRNA. |
| XM_929387.1 | LOC646463 |  | PREDICTED: Homo sapiens similar to Ubiquitin-conjugating enzyme E2 H (Ubiquitin-protein ligase H) (Ubiquitin carrier protein H) (UBCH2) (E2-20K) (LOC646463), mRNA. |
| NM_001101426.2 | LOC729920 |  | Homo sapiens notch1-induced protein (LOC729920), transcript variant 1, mRNA. |
| NM_203446.1 | SYNJ1 | 21 | Homo sapiens synaptojanin 1 (SYNJ1), transcript variant 2, mRNA. |
| NM_004289.5 | NFE2L3 | 7 | Homo sapiens nuclear factor (erythroid-derived 2)-like 3 (NFE2L3), mRNA. |
| NM_003908.3 | EIF2S2 | 20 | Homo sapiens eukaryotic translation initiation factor 2, subunit 2 beta, 38kDa (EIF2S2), mRNA. |
| NM_000422.1 | KRT17 | 17 | Homo sapiens keratin 17 (KRT17), mRNA. |
| NM_005128.2 | DOPEY2 | 21 | Homo sapiens dopey family member 2 (DOPEY2), mRNA. |
| NM_031916.2 | ROPN1L | 5 | Homo sapiens ropporin 1-like (ROPN1L), mRNA. |
| NM_144504.1 | F11R | 1 | Homo sapiens F11 receptor (F11R), transcript variant 5, mRNA. |
| NM_003045.3 | SLC7A1 | 13 | Homo sapiens solute carrier family 7 (cationic amino acid transporter, y+ system), member 1 (SLC7A1), mRNA. |
| NM_031910.3 | C1QTNF6 | 22 | Homo sapiens C1q and tumor necrosis factor related protein 6 (C1QTNF6), transcript variant 1, mRNA. |
| NM_017633.1 | FAM46A | 6 | Homo sapiens family with sequence similarity 46, member A (FAM46A), mRNA. |
| NM_003115.3 | UAP1 | 1 | Homo sapiens UDP-N-acteylglucosamine pyrophosphorylase 1 (UAP1), mRNA. |
| NM_002149.2 | HPCAL1 | 2 | Homo sapiens hippocalcin-like 1 (HPCAL1), transcript variant 1, mRNA. |
| NM_003507.1 | FZD7 | 2 | Homo sapiens frizzled homolog 7 (Drosophila) (FZD7), mRNA. |
| NM_020390.5 | EIF5A2 | 3 | Homo sapiens eukaryotic translation initiation factor 5A2 (EIF5A2), mRNA. |
| NM_020337.1 | ANKRD50 | 4 | Homo sapiens ankyrin repeat domain 50 (ANKRD50), mRNA. |
| NM_144567.3 | ANGEL2 | 1 | Homo sapiens angel homolog 2 (Drosophila) (ANGEL2), mRNA. |
| NM_001175.4 | ARHGDIB | 12 | Homo sapiens Rho GDP dissociation inhibitor (GDI) beta (ARHGDIB), mRNA. |
| NM_013373.2 | ZDHHC8 | 22 | Homo sapiens zinc finger, DHHC-type containing 8 (ZDHHC8), mRNA. |
| NM_173217.1 | ST6GAL1 | 3 | Homo sapiens ST6 beta-galactosamide alpha-2,6-sialyltranferase 1 (ST6GAL1), transcript variant 2, mRNA. |
| XM_498969.2 | LOC441019 | 4 | PREDICTED: Homo sapiens hypothetical LOC441019 (LOC441019), mRNA. |
| NR_002187.1 | LOC286016 | 7 | Homo sapiens triosephosphate isomerase 1 pseudogene (LOC286016), non-coding RNA. |
| NM_021194.2 | SLC30A1 | 1 | Homo sapiens solute carrier family 30 (zinc transporter), member 1 (SLC30A1), mRNA. |
| NM_020651.2 | PELI1 | 2 | Homo sapiens pellino homolog 1 (Drosophila) (PELI1), mRNA. |
| NM_025204.2 | TRABD | 22 | Homo sapiens TraB domain containing (TRABD), mRNA. |
| NM_003683.5 | RRP1 | 21 | Homo sapiens ribosomal RNA processing 1 homolog (S. cerevisiae) (RRP1), mRNA. |
| NM_015995.2 | KLF13 | 15 | Homo sapiens Kruppel-like factor 13 (KLF13), mRNA. |
| NM_152400.1 | C4ORF32 | 4 | Homo sapiens chromosome 4 open reading frame 32 (C4orf32), mRNA. |
| XM_927280.1 | LOC644033 | 8 | PREDICTED: Homo sapiens similar to similar to RPL23AP7 protein (LOC644033), mRNA. |
| NM_004512.3 | IL11RA | 9 | Homo sapiens interleukin 11 receptor, alpha (IL11RA), transcript variant 1, mRNA. |
| NM_134426.1 | SLC26A6 | 3 | Homo sapiens solute carrier family 26, member 6 (SLC26A6), transcript variant 3, mRNA. |
| NM_003784.1 | SERPINB7 | 18 | Homo sapiens serpin peptidase inhibitor, clade B (ovalbumin), member 7 (SERPINB7), transcript variant 1, mRNA. |
| NM_002271.4 | IPO5 | 13 | Homo sapiens importin 5 (IPO5), mRNA. |
| NM_014889.2 | PITRM1 | 10 | Homo sapiens pitrilysin metallopeptidase 1 (PITRM1), mRNA. |
| NM_032656.2 | DHX37 | 12 | Homo sapiens DEAH (Asp-Glu-Ala-His) box polypeptide 37 (DHX37), mRNA. |
| NM_016034.2 | MRPS2 | 9 | Homo sapiens mitochondrial ribosomal protein S2 (MRPS2), nuclear gene encoding mitochondrial protein, mRNA. |
| NM_033285.2 | TP53INP1 | 8 | Homo sapiens tumor protein p53 inducible nuclear protein 1 (TP53INP1), mRNA. |
| NM_002141.2 | HOXA4 | 7 | Homo sapiens homeobox A4 (HOXA4), mRNA. |
| NM_032318.1 | HIATL1 | 9 | Homo sapiens hippocampus abundant transcript-like 1 (HIATL1), mRNA. |
| NR_002174.1 | CMAH | 6 | Homo sapiens cytidine monophosphate-N-acetylneuraminic acid hydroxylase (CMP-N-acetylneuraminate monooxygenase) pseudogene (CMAH), transcript variant 1, non-coding RNA. |
| XM_946070.1 | USP24 | 1 | Homo sapiens ubiquitin specific peptidase 24 (USP24), mRNA. |
| NM_145056.1 | DACT3 | 19 | Homo sapiens dapper, antagonist of beta-catenin, homolog 3 (Xenopus laevis) (DACT3), mRNA. |
| NM_031948.3 | PRSS27 | 16 | Homo sapiens protease, serine 27 (PRSS27), mRNA. |
| NM_005953.2 | MT2A | 16 | Homo sapiens metallothionein 2A (MT2A), mRNA. |
| NM_001481.1 | GAS8 | 16 | Homo sapiens growth arrest-specific 8 (GAS8), mRNA. |
| NM_002528.4 | NTHL1 | 16 | Homo sapiens nth endonuclease III-like 1 (E. coli) (NTHL1), mRNA. |
| NM_015360.2 | SKIV2L2 | 5 | Homo sapiens superkiller viralicidic activity 2-like 2 (S. cerevisiae) (SKIV2L2), mRNA. |
| NM_002408.3 | MGAT2 | 14 | Homo sapiens mannosyl (alpha-1,6-)-glycoprotein beta-1,2-N-acetylglucosaminyltransferase (MGAT2), mRNA. |
| NM_030915.1 | LBH | 2 | Homo sapiens limb bud and heart development homolog (mouse) (LBH), mRNA. |
