# Supplementary material for: The bromodomain inhibitor OTX015 (MK-8628) exerts anti-tumor activity in triple-negative breast cancer models as single agent and in combination with everolimus
Source: Oncotarget. 2016 Dec 7;8(5):7598–613. doi: 10.18632/oncotarget.13814 (PMC5352346; doi:10.18632/oncotarget.13814)
Supplement: Supplementary file 3 [file oncotarget-08-7598-s003.docx]

| Symbol | Definition | | Synonyms |
| --- | --- | --- | --- |
| HIST1H2BD | Homo sapiens histone cluster 1, H2bd (HIST1H2BD), transcript variant 2, mRNA. | H2B.1B; HIRIP2; MGC90432; dJ221C16.6; H2B/b; H2BFB | |
| HIST1H2BD | Homo sapiens histone cluster 1, H2bd (HIST1H2BD), transcript variant 2, mRNA. | H2B.1B; HIRIP2; MGC90432; dJ221C16.6; H2B/b; H2BFB | |
| HIST1H2AC | Homo sapiens histone cluster 1, H2ac (HIST1H2AC), mRNA. | dJ221C16.4; H2AFL; H2A/l; MGC99519 | |
| CDKN1A | Homo sapiens cyclin-dependent kinase inhibitor 1A (p21, Cip1) (CDKN1A), transcript variant 1, mRNA. | P21; MDA-6; SDI1; p21CIP1; CIP1; CDKN1; WAF1; CAP20 | |
| FGFR3 | Homo sapiens fibroblast growth factor receptor 3 (achondroplasia, thanatophoric dwarfism) (FGFR3), transcript variant 2, mRNA. | HSFGFR3EX; JTK4; CD333; ACH; CEK2 | |
| TUBB3 | Homo sapiens tubulin, beta 3 (TUBB3), mRNA. | TUBB4; MC1R; beta-4 | |
| HIST1H2BK | Homo sapiens histone cluster 1, H2bk (HIST1H2BK), mRNA. | MGC131989; H2BFT; H2BFAiii; H2B/S | |
| HIST2H2AA3 | Homo sapiens histone cluster 2, H2aa3 (HIST2H2AA3), mRNA. | H2a-615; H2A; H2AFO; HIST2H2AA; H2A/q; H2A/O; H2A.2 | |
|  | Homo sapiens clone 24841 mRNA sequence |  | |
| MT1X | Homo sapiens metallothionein 1X (MT1X), mRNA. | MT1; MT-1l | |
| H1F0 | Homo sapiens H1 histone family, member 0 (H1F0), mRNA. | H1FV; MGC5241; H10 | |
| HIST1H4H | Homo sapiens histone cluster 1, H4h (HIST1H4H), mRNA. | H4FH; H4/h | |
| LOC284023 | PREDICTED: Homo sapiens hypothetical protein LOC284023, transcript variant 3 (LOC284023), mRNA. | | |
| HIST2H2BE | Homo sapiens histone cluster 2, H2be (HIST2H2BE), mRNA. | H2BFQ; MGC119804; MGC129733; H2B/q; GL105; MGC129734; H2B; MGC119802; H2B.1 | |
| HIST2H2AA4 | Homo sapiens histone cluster 2, H2aa4 (HIST2H2AA4), mRNA. | H2A/R | |
| HIST1H2BK | Homo sapiens histone cluster 1, H2bk (HIST1H2BK), mRNA. | MGC131989; H2BFT; H2BFAiii; H2B/S | |
| IRF7 | Homo sapiens interferon regulatory factor 7 (IRF7), transcript variant b, mRNA. | IRF7A; IRF-7H | |
| TUFT1 | Homo sapiens tuftelin 1 (TUFT1), mRNA. |  | |
| NXF1 | Homo sapiens nuclear RNA export factor 1 (NXF1), transcript variant 1, mRNA. | MEX67; TAP; DKFZp667O0311 | |
| VASN | Homo sapiens vasorin (VASN), mRNA. | SLITL2 | |
|  | Homo sapiens cDNA clone IMAGE:4811759 |  | |
| DCXR | Homo sapiens dicarbonyl/L-xylulose reductase (DCXR), mRNA. | P34H; HCRII; HCR2; KIDCR; DCR | |
| BRD2 | Homo sapiens bromodomain containing 2 (BRD2), mRNA. | FLJ31942; RNF3; D6S113E; RING3; DKFZp686N0336; NAT; FSRG1; KIAA9001 | |
| HES6 | Homo sapiens hairy and enhancer of split 6 (Drosophila) (HES6), mRNA. | | |
| LOC653506 | PREDICTED: Homo sapiens similar to meteorin, glial cell differentiation regulator-like (LOC653506), mRNA. | | |
| SERTAD1 | Homo sapiens SERTA domain containing 1 (SERTAD1), mRNA. | TRIP-Br1; SEI1 | |
| HIST1H2BJ | Homo sapiens histone cluster 1, H2bj (HIST1H2BJ), mRNA. | H2B/r; H2BFR | |
| SERPINI1 | Homo sapiens serpin peptidase inhibitor, clade I (neuroserpin), member 1 (SERPINI1), mRNA. | neuroserpin; PI12; DKFZp781N13156 | |
| HIST1H1C | Homo sapiens histone cluster 1, H1c (HIST1H1C), mRNA. | MGC3992; H1F2; H1.2 | |
| CHCHD7 | Homo sapiens coiled-coil-helix-coiled-coil-helix domain containing 7 (CHCHD7), transcript variant 6, mRNA. | FLJ40966; MGC2217 | |
| TUBB4Q | Homo sapiens tubulin, beta polypeptide 4, member Q (TUBB4Q), mRNA. | | |
| HIST2H2AC | Homo sapiens histone cluster 2, H2ac (HIST2H2AC), mRNA. | H2AFQ; MGC74460; H2A; H2A/q; H2A-GL101 | |
| EPB41L5 | Homo sapiens erythrocyte membrane protein band 4.1 like 5 (EPB41L5), mRNA. | FLJ12957; KIAA1548; BE37 | |
| NDRG1 | Homo sapiens N-myc downstream regulated gene 1 (NDRG1), mRNA. | CMT4D; GC4; RTP; RIT42; PROXY1; NMSL; NDR1; TDD5; TARG1; CAP43; HMSNL; DRG1 | |
| LOC441019 | PREDICTED: Homo sapiens hypothetical LOC441019 (LOC441019), mRNA. | | |
| NXF1 | Homo sapiens nuclear RNA export factor 1 (NXF1), transcript variant 2, mRNA. | MEX67; TAP; DKFZp667O0311 | |
| HIST2H2AA3 | Homo sapiens histone cluster 2, H2aa3 (HIST2H2AA3), mRNA. | H2a-615; H2A; H2AFO; HIST2H2AA; H2A/q; H2A/O; H2A.2 | |
| SIPA1L2 | Homo sapiens signal-induced proliferation-associated 1 like 2 (SIPA1L2), mRNA. | SPAL2; FLJ23126; KIAA1389; FLJ23632 | |

**Supplementary Table S3.** Common up-regulated genes in MDA-MB-231 and MDA-MB-468 cell lines.
