# Supplementary material for: The bromodomain inhibitor OTX015 (MK-8628) exerts anti-tumor activity in triple-negative breast cancer models as single agent and in combination with everolimus
Source: Oncotarget. 2016 Dec 7;8(5):7598–613. doi: 10.18632/oncotarget.13814 (PMC5352346; doi:10.18632/oncotarget.13814)
Supplement: Supplementary file 4 [file oncotarget-08-7598-s004.docx]

**Supplementary Table S5** MSIGDB GSEA report.

|  | NAME | SIZE | ES | NES | NOM p-val | FDR q-val | FWER p-val | RANK AT MAX | LEADING EDGE | |
| --- | --- | --- | --- | --- | --- | --- | --- | --- | --- | --- |
| Down-regulated gene sets | MANALO_HYPOXIA_DN | 271 | -0.5728669 | -27.352.948 | 0.0 | 0.0 | 0.0 | 4373 | tags=49%, list=13%, signal=56% | |
|  | MORF_BUB1B | 64 | -0.7006893 | -2.664.175 | 0.0 | 0.0 | 0.0 | 5779 | tags=69%, list=17%, signal=82% | |
|  | GNF2_CCNA2 | 67 | -0.6633927 | -2.602.649 | 0.0 | 0.0 | 0.0 | 4315 | tags=64%, list=12%, signal=73% | |
|  | WONG_EMBRYONIC_STEM_CELL_CORE | 325 | -0.5302715 | -25.871.398 | 0.0 | 0.0 | 0.0 | 3889 | tags=43%, list=11%, signal=48% | |
|  | GNF2_CKS1B | 37 | -0.7478525 | -25.807.495 | 0.0 | 0.0 | 0.0 | 5011 | tags=73%, list=14%, signal=85% | |
|  | GNF2_PA2G4 | 78 | -0.6311813 | -25.474.026 | 0.0 | 0.0 | 0.0 | 3723 | tags=54%, list=11%, signal=60% | |
|  | MORF_BUB1 | 50 | -0.6959255 | -25.324.283 | 0.0 | 0.0 | 0.0 | 5779 | tags=68%, list=17%, signal=82% | |
|  | GNF2_RAN | 84 | -0.6285962 | -25.241.303 | 0.0 | 0.0 | 0.0 | 3550 | tags=51%, list=10%, signal=57% | |
|  | GNF2_HMMR | 46 | -0.6970267 | -25.226.805 | 0.0 | 0.0 | 0.0 | 4315 | tags=70%, list=12%, signal=79% | |
|  | GNF2_SMC4L1 | 82 | -0.6309158 | -25.196.364 | 0.0 | 0.0 | 0.0 | 3889 | tags=57%, list=11%, signal=64% | |
|  | GNF2_RFC4 | 59 | -0.6570999 | -25.195.937 | 0.0 | 0.0 | 0.0 | 4214 | tags=64%, list=12%, signal=73% | |
|  | HALLMARK_MYC_TARGETS_V2 | 57 | -0.6702081 | -2.516.878 | 0.0 | 0.0 | 0.0 | 4065 | tags=58%, list=12%, signal=65% | |
|  | ZHANG_TLX_TARGETS_60HR_DN | 259 | -0.5276511 | -24.879.763 | 0.0 | 0.0 | 0.0 | 5075 | tags=50%, list=15%, signal=58% | |
|  | GNF2_PCNA | 67 | -0.6388856 | -24.763.825 | 0.0 | 0.0 | 0.0 | 4315 | tags=64%, list=12%, signal=73% | |
|  | GNF2_CDC20 | 55 | -0.6575663 | -2.462.655 | 0.0 | 0.0 | 0.0 | 4315 | tags=62%, list=12%, signal=71% | |
|  | GNF2_RRM1 | 87 | -0.6115587 | -24.613.938 | 0.0 | 0.0 | 0.0 | 4315 | tags=55%, list=12%, signal=63% | |
|  | GSE22886_UNSTIM_VS_IL2_STIM_NKCELL_DN | 188 | -0.5436639 | -2.453.335 | 0.0 | 0.0 | 0.0 | 3715 | tags=44%, list=11%, signal=49% | |
|  | GNF2_FEN1 | 55 | -0.654896 | -24.422.567 | 0.0 | 0.0 | 0.0 | 3889 | tags=60%, list=11%, signal=67% | |
|  | TOYOTA_TARGETS_OF_MIR34B_AND_MIR34C | 414 | -0.4866399 | -24.379.604 | 0.0 | 0.0 | 0.0 | 5271 | tags=44%, list=15%, signal=51% | |
|  |  |  |  |  |  |  |  |  |  |  |
| Up-regulated gene sets | MANALO_HYPOXIA_DN | 443 | 0.6004877 | 30.465.183 | 0.0 | 0.0 | 0.0 | 4250 | tags=45%, list=12%, signal=51% | |
|  | HELLER_HDAC_TARGETS_UP | 303 | 0.61179817 | 29.996.257 | 0.0 | 0.0 | 0.0 | 4940 | tags=50%, list=14%, signal=58% | |
|  | REACTOME_AMYLOIDS | 80 | 0.697706 | 28.086.095 | 0.0 | 0.0 | 0.0 | 3260 | tags=46%, list=9%, signal=51% | |
|  | REACTOME_PACKAGING_OF_TELOMERE_ENDS | 48 | 0.7472523 | 28.084.164 | 0.0 | 0.0 | 0.0 | 3260 | tags=56%, list=9%, signal=62% | |
|  | REACTOME_RNA_POL_I_PROMOTER_OPENING | 59 | 0.7365531 | 27.897.666 | 0.0 | 0.0 | 0.0 | 3260 | tags=58%, list=9%, signal=64% | |
|  | ZHONG_RESPONSE_TO_AZACITIDINE_AND_TSA_UP | 175 | 0.58826226 | 27.099.695 | 0.0 | 0.0 | 0.0 | 3915 | tags=44%, list=11%, signal=49% | |
|  | REACTOME_RNA_POL_I_TRANSCRIPTION | 86 | 0.6549667 | 26.962.914 | 0.0 | 0.0 | 0.0 | 3260 | tags=48%, list=9%, signal=53% | |
|  | KRCTCNNNNMANAGC_UNKNOWN | 63 | 0.65409654 | 26.090.024 | 0.0 | 0.0 | 0.0 | 3653 | tags=43%, list=11%, signal=48% | |
|  |  |  |  |  |  |  |  |  |  | |
|  |  |  |  |  |  |  |  |  |  | |
| Up-regulated gene sets | MANALO_HYPOXIA_DN | 124 | 0.58474314 | 25.846.405 | 0.0 | 0.0 | 0.0 | 4956 | tags=49%, list=14%, signal=57% | |
|  | CHIBA_RESPONSE_TO_TSA | 45 | 0.71000475 | 25.747.643 | 0.0 | 0.0 | 0.0 | 2717 | tags=56%, list=8%, signal=60% | |
|  | KEGG_SYSTEMIC_LUPUS_ERYTHEMATOSUS | 136 | 0.5825021 | 25.687.387 | 0.0 | 0.0 | 0.0 | 4805 | tags=41%, list=14%, signal=48% | |
|  | NAGASHIMA_EGF_SIGNALING_UP | 56 | 0.6683864 | 25.675.795 | 0.0 | 0.0 | 0.0 | 3689 | tags=63%, list=11%, signal=70% | |
|  | REACTOME_MEIOTIC_RECOMBINATION | 83 | 0.6051296 | 24.848.912 | 0.0 | 0.0 | 0.0 | 2903 | tags=41%, list=8%, signal=45% | |
|  | MITSIADES_RESPONSE_TO_APLIDIN_UP | 408 | 0.4886413 | 24.816.575 | 0.0 | 0.0 | 0.0 | 4948 | tags=40%, list=14%, signal=46% | |
|  | NAGASHIMA_NRG1_SIGNALING_UP | 169 | 0.5455657 | 24.740.455 | 0.0 | 0.0 | 0.0 | 3797 | tags=46%, list=11%, signal=52% | |
|  | DACOSTA_UV_RESPONSE_VIA_ERCC3_COMMON_UP | 73 | 0.60783666 | 24.632.463 | 0.0 | 0.0 | 0.0 | 4779 | tags=58%, list=14%, signal=67% | |
|  | MENSE_HYPOXIA_UP | 87 | 0.5858829 | 24.591.274 | 0.0 | 0.0 | 0.0 | 5015 | tags=52%, list=14%, signal=60% | |
|  | WELCSH_BRCA1_TARGETS_UP | 191 | 0.5208002 | 24.105.403 | 0.0 | 0.0 | 0.0 | 4323 | tags=42%, list=12%, signal=48% | |
|  | REACTOME_MEIOTIC_SYNAPSIS | 72 | 0.6013362 | 2.401.278 | 0.0 | 0.0 | 0.0 | 3346 | tags=40%, list=10%, signal=44% | |

**Supplementary Table S6.** CMAP GSEA report.

|  | NAME | SIZE | ES | NES | NOM p-val | FDR q-val | FWER p-val | RANK AT MAX | LEADING EDGE |
| --- | --- | --- | --- | --- | --- | --- | --- | --- | --- |
| Down-regulated gene sets | SCRIPTAID_DN | 91 | -0.8069037 | -32.952.507 | 0.0 | 0.0 | 0.0 | 4286 | tags=77%, list=12%, signal=88% |
|  | TRICHOSTATINA_DN | 88 | -0.73390794 | -29.551.344 | 0.0 | 0.0 | 0.0 | 5289 | tags=68%, list=15%, signal=80% |
|  | VORINOSTAT_DN | 89 | -0.71259314 | -29.296.067 | 0.0 | 0.0 | 0.0 | 4532 | tags=66%, list=13%, signal=76% |
|  | CP69033401_DN | 93 | -0.70641 | -28.915.854 | 0.0 | 0.0 | 0.0 | 3942 | tags=62%, list=11%, signal=70% |
|  | LY294002_DN | 90 | -0.6774153 | -27.630.875 | 0.0 | 0.0 | 0.0 | 5522 | tags=61%, list=16%, signal=73% |
|  | HCTOXIN_DN | 87 | -0.6797684 | -27.596.562 | 0.0 | 0.0 | 0.0 | 5201 | tags=62%, list=15%, signal=73% |
|  | DILAZEP_DN | 89 | -0.6502421 | -26.339.602 | 0.0 | 0.0 | 0.0 | 4562 | tags=54%, list=13%, signal=62% |
|  | THIORIDAZINE_DN | 95 | -0.6133496 | -25.251.255 | 0.0 | 0.0 | 0.0 | 4058 | tags=46%, list=12%, signal=52% |
|  | RIFABUTIN_DN | 87 | -0.62427664 | -25.133.743 | 0.0 | 0.0 | 0.0 | 2688 | tags=47%, list=8%, signal=51% |
|  | RESVERATROL_DN | 94 | -0.5938521 | -2.442.915 | 0.0 | 0.0 | 0.0 | 5964 | tags=66%, list=17%, signal=79% |
|  | DAUNORUBICIN_DN | 91 | -0.5960902 | -2.427.633 | 0.0 | 0.0 | 0.0 | 4315 | tags=52%, list=12%, signal=59% |
|  | ALPHAERGOCRYPTINE_DN | 91 | -0.58283246 | -23.998.356 | 0.0 | 0.0 | 0.0 | 4206 | tags=49%, list=12%, signal=56% |
|  | FLUPHENAZINE_DN | 94 | -0.5848217 | -23.910.494 | 0.0 | 0.0 | 0.0 | 4371 | tags=46%, list=13%, signal=52% |
|  | WITHAFERINA_DN | 96 | -0.5702705 | -23.609.002 | 0.0 | 0.0 | 0.0 | 4237 | tags=51%, list=12%, signal=58% |
|  | ETOPOSIDE_DN | 94 | -0.5664383 | -23.378.172 | 0.0 | 0.0 | 0.0 | 5964 | tags=60%, list=17%, signal=72% |
|  | MG262_DN | 91 | -0.56221247 | -23.222.892 | 0.0 | 0.0 | 0.0 | 4023 | tags=53%, list=12%, signal=60% |
|  | LOPERAMIDE_DN | 89 | -0.568344 | -2.313.042 | 0.0 | 0.0 | 0.0 | 6396 | tags=54%, list=18%, signal=66% |
|  | PROCHLORPERAZINE_DN | 90 | -0.56227297 | -2.310.962 | 0.0 | 0.0 | 0.0 | 3538 | tags=44%, list=10%, signal=49% |
|  | CICLOPIROX_DN | 93 | -0.5619782 | -23.047.595 | 0.0 | 0.0 | 0.0 | 5804 | tags=58%, list=17%, signal=70% |
|  | RALOXIFENE_DN | 93 | -0.5574779 | -2.277.812 | 0.0 | 0.0 | 0.0 | 4132 | tags=46%, list=12%, signal=52% |
|  |  |  |  |  |  |  |  |  |  |
| Up-regulated gene sets | TRICHOSTATINA_UP | 97 | 0.78676033 | 32.929.974 | 0.0 | 0.0 | 0.0 | 4856 | tags=69%, list=14%, signal=80% |
|  | VORINOSTAT_UP | 96 | 0.7783864 | 32.682.884 | 0.0 | 0.0 | 0.0 | 4956 | tags=73%, list=14%, signal=85% |
|  | SCRIPTAID_UP | 92 | 0.74693954 | 31.263.273 | 0.0 | 0.0 | 0.0 | 4730 | tags=66%, list=14%, signal=77% |
|  | CP69033401_UP | 90 | 0.72594976 | 30.899.427 | 0.0 | 0.0 | 0.0 | 3858 | tags=56%, list=11%, signal=62% |
|  | LY294002_UP | 94 | 0.70456916 | 2.979.637 | 0.0 | 0.0 | 0.0 | 4515 | tags=60%, list=13%, signal=68% |
|  | DISULFIRAM_UP | 94 | 0.6932673 | 2.957.021 | 0.0 | 0.0 | 0.0 | 4956 | tags=61%, list=14%, signal=71% |
|  | PIPERLONGUMINE_UP | 93 | 0.6977474 | 29.325.442 | 0.0 | 0.0 | 0.0 | 2820 | tags=51%, list=8%, signal=55% |
|  | LANATOSIDEC_UP | 95 | 0.69298136 | 2.907.087 | 0.0 | 0.0 | 0.0 | 4956 | tags=64%, list=14%, signal=75% |
|  | HCTOXIN_UP | 92 | 0.6862044 | 28.456.874 | 0.0 | 0.0 | 0.0 | 4856 | tags=61%, list=14%, signal=71% |
|  | CAMPTOTHECIN_UP | 92 | 0.6679622 | 28.236.914 | 0.0 | 0.0 | 0.0 | 3689 | tags=50%, list=11%, signal=56% |
|  | HELVETICOSIDE_UP | 96 | 0.66971654 | 28.194.416 | 0.0 | 0.0 | 0.0 | 5164 | tags=60%, list=15%, signal=71% |
|  | DIGITOXIGENIN_UP | 95 | 0.6723771 | 28.166.614 | 0.0 | 0.0 | 0.0 | 2886 | tags=52%, list=8%, signal=56% |
|  | TRIFLUOPERAZINE_UP | 99 | 0.6513055 | 2.795.079 | 0.0 | 0.0 | 0.0 | 3934 | tags=60%, list=11%, signal=67% |
| Up-regulated gene sets | ANISOMYCIN_UP | 95 | 0.65949863 | 27.576.478 | 0.0 | 0.0 | 0.0 | 2961 | tags=49%, list=9%, signal=54% |
|  | RESVERATROL_UP | 95 | 0.657003 | 27.393.298 | 0.0 | 0.0 | 0.0 | 3224 | tags=49%, list=9%, signal=54% |
|  | THIORIDAZINE_UP | 99 | 0.6454051 | 27.328.987 | 0.0 | 0.0 | 0.0 | 4956 | tags=63%, list=14%, signal=73% |
|  | DILAZEP_UP | 91 | 0.64904124 | 27.055.762 | 0.0 | 0.0 | 0.0 | 4506 | tags=54%, list=13%, signal=62% |
|  | PHENOXYBENZAMINE_UP | 90 | 0.64364564 | 26.914.275 | 0.0 | 0.0 | 0.0 | 3442 | tags=54%, list=10%, signal=60% |
|  | CELASTROL_UP | 94 | 0.63733995 | 2.684.086 | 0.0 | 0.0 | 0.0 | 2820 | tags=50%, list=8%, signal=54% |
|  | MEFLOQUINE_UP | 95 | 0.6402048 | 2.676.927 | 0.0 | 0.0 | 0.0 | 4162 | tags=55%, list=12%, signal=62% |
